# Whole genome re-sequencing of date palms yields insights into diversification of a fruit tree crop

**DOI:** 10.1038/ncomms9824

**Published:** 2015-11-09

**Authors:** Khaled M. Hazzouri, Jonathan M. Flowers, Hendrik J. Visser, Hussam S. M. Khierallah, Ulises Rosas, Gina M. Pham, Rachel S. Meyer, Caryn K. Johansen, Zoë A. Fresquez, Khaled Masmoudi, Nadia Haider, Nabila El Kadri, Youssef Idaghdour, Joel A. Malek, Deborah Thirkhill, Ghulam S. Markhand, Robert R. Krueger, Abdelouahhab Zaid, Michael D. Purugganan

**Affiliations:** 1 Center for Genomics and Systems Biology, New York University Abu Dhabi, Saadiyat Island, PO Box 129188, Abu Dhabi, United Arab Emirates; 2 Department of Biology, Center for Genomics and Systems Biology, 12 Waverly Place, New York University, New York, New York 10003, USA; 3 Date Palm Research and Development Unit, United Arab Emirates University, Al-Ain, PO Box 15551, Abu Dhabi, United Arab Emirates; 4 Date Palm Research Unit, College of Agriculture, PO Box 47054, University of Baghdad, Baghdad, Iraq; 5 International Center for Biosaline Agriculture, Academic City, Al Ruwayyah 2, PO Box 14660, Dubai, United Arab Emirates; 6 Department of Molecular Biology and Biotechnology, Atomic Energy Commission of Syria, PO Box 6091, Damascus, Syria; 7 Technical Center of Dates, Ministry of Agriculture, Kebili, Tunisia; 8 Division of Science and Mathematics, New York University Abu Dhabi, Saadiyat Island, PO Box 129188, Abu Dhabi, United Arab Emirates; 9 Genomics Core Laboratory, Weill Cornell Medical College in Qatar, Doha 24144, Qatar; 10 Arizona State University Date Palm Collection, Arizona State University Tempe, Arizona, Arizona 85281, USA; 11 Date Palm Research Institute (DPRI), Shah Abdul Latif University, Khairpur, Sindh, Pakistan; 12 United States Department of Agriculture, Riverside, California 92507, USA

## Abstract

Date palms (*Phoenix dactylifera*) are the most significant perennial crop in arid regions of the Middle East and North Africa. Here, we present a comprehensive catalogue of approximately seven million single nucleotide polymorphisms in date palms based on whole genome re-sequencing of a collection of 62 cultivars. Population structure analysis indicates a major genetic divide between North Africa and the Middle East/South Asian date palms, with evidence of admixture in cultivars from Egypt and Sudan. Genome-wide scans for selection suggest at least 56 genomic regions associated with selective sweeps that may underlie geographic adaptation. We report candidate mutations for trait variation, including nonsense polymorphisms and presence/absence variation in gene content in pathways for key agronomic traits. We also identify a *copia*-like retrotransposon insertion polymorphism in the R2R3 myb-like orthologue of the oil palm *virescens* gene associated with fruit colour variation. This analysis documents patterns of post-domestication diversification and provides a genomic resource for this economically important perennial tree crop.

## Abstract (web summary)

Date palm is a perennial fruit tree crop that thrives in arid environments of North Africa and the Middle East. Here Hazzouri *et al*. analyze the date palm germplasm by re-sequencing 62 different varieties and uncover selection signatures and candidate genes associated with important agronomic traits.

## Graphical abstract

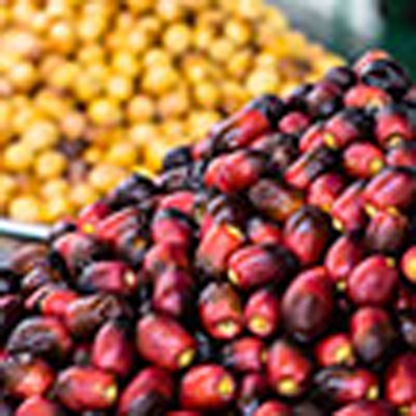

The last decade has been an intensive time for studying the genetic basis of crop domestication and diversification^1^,^2^,^3^. Much of the work has focused on annual crop species^1^,^3^, particularly the cereal crops where patterns of genome diversity have been characterized. Genomic resources in annual crops, such as development of single nucleotide polymorphism (SNP) maps of genomes, have led to the identification of numerous loci underlying phenotypic diversity^4^,^5^. In contrast, there has been relatively little attention paid to perennial crops, including fruit tree species. Perennial crops, given their diverse life histories and breeding systems, are likely to evolve in distinct ways from annual crop species^6^. Moreover, the availability of genomic resources in these long-lived crop taxa will be necessary if we are to fully exploit phenotypic diversity and accelerate breeding to improve global food security, particularly in tree species^7^.

The date palm (*Phoenix dactylifera* L.) is a dioecious, perennial diploid (2*n*=36) tree in the Arecaceae family and is the most important fruit-bearing crop in arid regions of the Middle East and North Africa^8^,^9^,^10^. Date palms grow primarily in hot, arid habitats including desert oases, river valleys and well-irrigated farms or plantations. Individual varieties are valued primarily for fruit-related traits including moisture and sugar content, and as many as 3,000 varieties are recognized worldwide^10^. The recent completion of two assemblies of the 690 Mb genome of the Khalas variety by two independent groups^11^,^12^ has enabled new discoveries, including identification of the sex determination locus^11^ and characterization of pathways active during fruit maturation^12^. Both assemblies are presently in the draft stage with the more complete assembly^12^ consisting of 82,354 scaffolds with an N50 of 329.9 kb and a maximum scaffold size of ∼4.5 Mb. This assembled sequence covers 90.2% of the genome and contains 41,660 gene models.

The origin of *P. dactylifera* remains enigmatic, and no clear wild ancestor has been identified^9^. The sugar palm, *P. sylvestris*, has been considered to be a likely progenitor of domesticated date palm. This species currently grows wild in the Indus Valley and parts of India, and its historical range may have extended as far west as the Gulf region^13^. However, genetic data have not established a clear relationship between *P. dactylifera* and *P. sylvestris*, or any of several peri-patrically distributed species with which it is known to hybridize^14^. Recent work suggests the origin of domesticated dates to be in the Middle East^15^,^16^, which is supported by archaeological data in the region^15^,^16^,^17^. The Gulf region holds the oldest evidence of date palm exploitation, with seeds dating to 7100 BP excavated in site remains in Dalma Island, Abu Dhabi and Kuwait^15^,^16^,^17^. Although alternate hypotheses propose a domestication centre of date palm in North Africa^18^, this crop appears ∼3,000 years later in the archaeological records of this region^19^.

Domesticated date palms exhibit a wide range of phenotypic diversity in fruit colour, sugar content, flowering time and other agronomic traits. Much of this diversity is likely the result of evolutionary diversification that drove adaptation of date palm cultivars across its native range in Asia and Africa. Characterization of genome diversity in date palm cultivars and discovery of genes controlling traits of interest will improve the prospects of date palm breeding for yield and other agronomic traits while providing a means to answer long-standing questions about date palm diversity and the history of domestication.

Despite the central importance of date palm as a traditional crop in the Middle East and North Africa and the agronomic challenges to their continued sustainable cultivation, very little is known about the genomic diversity of this species. In this article, we present analyses of whole genome re-sequencing of 62 varieties of *P. dactylifera*. The sequenced cultivars originate from locations spanning the traditional range of date palm cultivation from North Africa to the Middle East, as well as newer production areas in Pakistan. Our samples include common commercial varieties from all of the major date-producing countries in the region, and our analysis provides the first comprehensive catalogue of molecular variation in this species.

## Results

### Variation in the date palm genome

We sequenced the genomes of 62 varieties of date palm from 12 countries spanning the traditional range of cultivation of this species. Seventeen of the cultivars are from Africa (North Africa, Egypt and Sudan), 36 from the Middle East (the Arabian peninsula, Iraq and Iran) and nine from South Asia (Pakistan; Supplementary Table 1), with the largest sample of 17 varieties in our data set originating from Iraq. Approximately, 10 of the samples are popular varieties that are commercially important and cultivated widely and, include Medjool, Deglet Noor, Barhee and Zahidi. The rest are grown in more restricted areas and in smaller numbers. Although date palms are primarily propagated vegetatively, many of the cultivars may be landraces^20^ and have not been subjected to scientific breeding.

**Figure 1:**
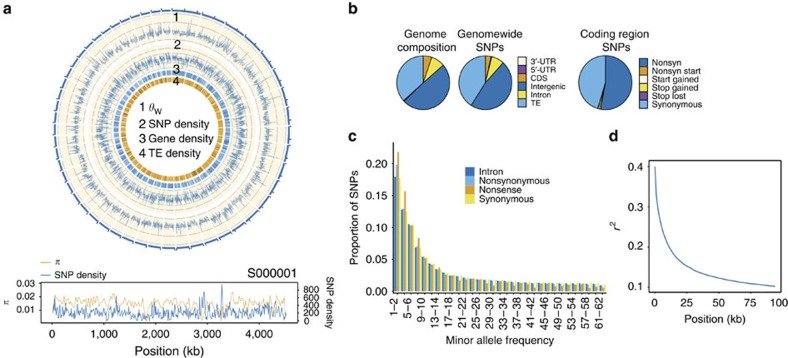
Summary of single nucleotide polymorphisms in 62 date palm cultivars. (**a**) Circos plot of the 50 longest scaffolds (18.4% of the cv. Khalas assembly)^12^. Tracks from outer to inner are *θ*_W_ (grid lines are drawn at 0.01 intervals) and SNP density (grid lines are drawn at 250-SNP intervals) in non-overlapping 25-kb bins. Gene density and transposable element densities in sliding windows of 100 kb with step size 20 kb. A zoom in scaffold S000001 showing *π* and SNP density in non-overlapping 10-kb bins (**b**) Distribution of SNPs among functional effect classes compared with the proportion of sites in the reference (cv. Khalas) genome. (**c**) Minor allele site-frequency spectrum among selected coding region site classes. (**d**) Decay of linkage disequilibrium measured as the squared correlation coefficient (*r*^2^) by physical distance in 62 cultivars.

Using paired-end (2 × 100 bp) Illumina sequencing, we obtained a mean sequencing depth of 20.8 × per sample when aligned to the cv. Khalas reference genome^12^ (Supplementary Table 2). After aligning the re-sequencing reads, we processed the alignments to remove duplicate reads, realigned reads around indels^21^,^22^, and applied a series of quality control filters with the intent of limiting false-positive variants^23^ (see Methods section). This procedure yielded 7,176,238 SNPs (excluding those found in transposable elements (TEs)), or ∼12 SNPs per kb, representing the most common sequence polymorphisms in cultivated date palm (Fig. 1a). An additional 4,933,882 SNPs are found in repetitive sequences, which we annotated as TE sequences^24^, and are excluded from our final SNP call set. To evaluate the quality of SNPs, we examined 37 genotypes by PCR amplification and Sanger sequencing and confirmed 36 of 37 (Supplementary Table 3), which represents a concordance rate of 97.2%.

The majority of non-TE SNPs in date palms (5,222,681 SNPs or 72.77%) are located in intergenic regions (Fig. 1b). SNPs in genic regions excluding TEs include 201,160 synonymous, 234,740 nonsynonymous (Fig. 1b), 1,479,953 intronic and 7,811 and 17,622 SNPs in 5′ and 3′ UTRs, respectively. The site-frequency spectrum for the various functional SNP classes indicates that both nonsynonymous and nonsense polymorphisms are skewed towards lower frequencies, suggesting they are enriched for slightly deleterious mutations (Fig. 1c).

We estimate the population mutation parameter defined as Watterson's theta (*θ*_W_) and nucleotide diversity (π) to be 0.01000±0.00003 and 0.0092±0.00002 (±represents standard error [s.e.], *n*=62), respectively (Supplementary Table 4). This indicates that pairs of randomly selected sequences on average differ at ∼1% of nucleotide sites. This estimate of nucleotide variation in date palm is higher than those for other perennial fruit crops, such as cassava (π=0.0026)^25^, peach (π=0.0015)^26^ and grapevine (π=0.0051)^27^.

Linkage disequilibrium (LD) decays relatively rapidly in date palms. LD measured as the squared correlation coefficient (*r*^2^) between SNPs decays to ∼50% of its maximum at ∼6 kb and 90% of its maximum at ∼40 kb (Fig. 1d). The majority of SNP pairs with *r*^2^ in near complete disequilibrium (>0.8) are found at physical distances less than 10 kb (Supplementary Fig. 1). This relatively rapid decay of LD suggests that genome-wide association studies (GWASs) should enable high-resolution mapping of genes associated with traits of agricultural significance.

### Population structure of date palms

**Figure 2:**
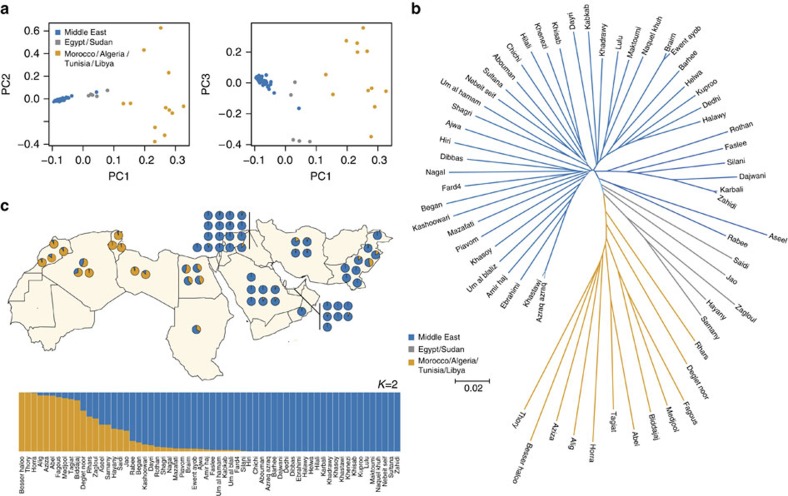
Population structure in cultivated date palm. (**a**) Principal component (PC) analysis of SNP genotypes based on ∼25,000 SNPs for 62 cultivars. PC1, 2 and 3 axes account for 11.96, 4.03 and 3.69% of the variation, respectively. (**b**) Neighbour-joining tree based on the distance metric of Gronau *et al*.^60^ using 7,176,238 SNPs. (**c**) Population stratification based on STRUCTURE for *K*=2. Pie charts represent admixture proportions (that is, percentage of genome composition associated with each ancestral subpopulation cluster), and are placed on the map in the traditionally recognized country of origin of each cultivar.

GWA mapping studies and modern breeding strategies, benefit from an understanding of population structure, LD and the genome-wide distribution of genetic variation^28^. Previous studies have suggested population differentiation within domesticated date palm^29^,^30^, and our genome-wide analysis confirms the presence of distinct North African and Middle Eastern/South Asian populations. This subdivision is apparent in the first axis of a principal component (PC) analysis^31^ (Fig. 2a) and in a neighbour-joining tree^32^ based on genetic distances from the whole genome SNP data (Fig. 2b). Using the population-based clustering method STRUCTURE^33^, we fit a model of population stratification in which an individual's genome is inferred to be composed of sites from up to *K* ancestral populations. We ran STRUCTURE for *K*=1–10 using the admixture model with correlated allele frequencies. Application of the Evanno method^34^ indicates that *K*=2 has the highest Δ*K* and therefore represents the best fit to the data (Fig. 2c; Supplementary Table 5), which is consistent with the distance-based and PC results. In the STRUCTURE analysis, the Middle Eastern and South Asian cultivars form a separate group, while the North African cultivars have a majority contribution of their genome from a population distinct from that found in the Middle East (Fig. 2c).

Our analysis provides evidence of genetic admixture between the predominant North African and Middle East populations in multiple varieties (Fig. 2c) consistent with hybridization between these two populations. The extent of admixture between the two regional populations ranges between <1 and 99% (Fig. 2c), with a number of samples appearing to be admixed between western and eastern subpopulations. Samples from the geographically intermediate areas of Egypt and Sudan are among the most admixed samples with 55–65% of their genomes being derived from the Middle Eastern population in the STRUCTURE analysis and appear as intermediate on the first axis of the PC analysis and a neighbour-joining tree. Admixture in samples from Egypt is consistent with a previous report^30^ and may indicate a hybrid origin of varieties cultivated in areas where the two subpopulations come into contact. Admixed samples from outside this region are also apparent (Fig. 2c), and may represent recent transfer of cultivars away from their country of origin^30^.

The North African population (excluding admixed samples such as those from Egypt and Sudan) has higher levels of nucleotide diversity compared with the Middle Eastern/South Asian population (π_North Africa_**=**0.01080±0.00003; ±s.e., *n*=10 versus π_Middle East/South Asia_=0.00810±0.00002; ± s.e., *n*=42; Supplementary Table 4). Higher diversity in Africa is also supported by estimates of *θ* from single diploid genomes^35^, which provides a more granular view of how diversity varies across the range of *P. dactylifera*. With one exception, all of the highest nucleotide diversity estimates from single genomes are from African samples (Supplementary Table 6), while single genome estimates outside of Africa have consistently lower levels of genetic diversity. These observations suggest that North African varieties are derived from a distinct North African population with a larger effective size (*N*_e_) than the Middle Eastern population.

### Genomic evidence of inbreeding in date palms

**Figure 3:**
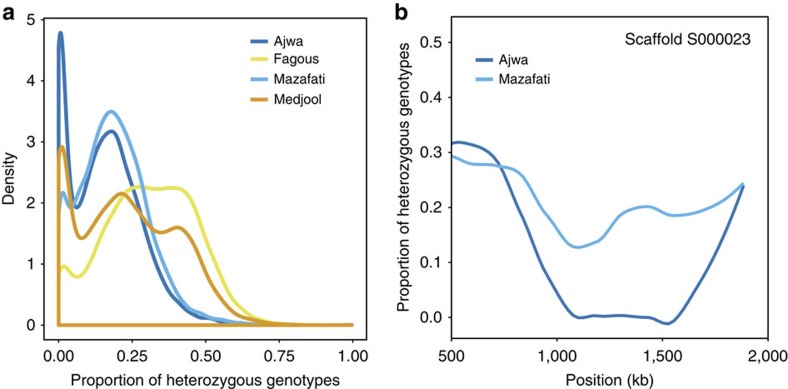
Evidence of inbreeding in date palm genomes. (**a**) Density distribution of the proportion of heterozygous genotypes in 50 kb windows for four date palm cultivars. Peaks in the distributions at low heterozygosity are apparent in the more inbred samples (**b**) An example run of homozygosity (ROH) in cv. Ajwa compared with cv. Mazafati on genome assembly scaffold S000023. Lines are loess fits to the proportion of heterozygous genotypes in non-overlapping 20 kb windows.

Date palms are obligate outcrossers, although cultivars show varying degrees of inbreeding. The individual inbreeding coefficient F_ind_ in different varieties ranges from completely outcrossed (F_ind_=1.2 × 10^−5^) to mildly inbred (F_ind_=0.125; Supplementary Table 6). More inbred cultivars such as cv. Ajwa and cv. Medjool have a large percentage (∼25%) of genomic segments that are almost completely homozygous, reflected in the bimodality in the distribution of heterozygosity in single genomes, with a peak near 0% heterozygosity (Fig. 3a). In contrast, outbred samples such as cv. Fagous and cv. Mazafati, have a more uniform genome-wide distribution, a higher average over-all heterozygosity, and relatively few genomic intervals that are predominantly homozygous (Fig. 3a).

In the most inbred samples, homozygous intervals are frequently found in long (spanning >500 kb) runs of homozygosity (ROH) with few heterozygous genotypes. This is evident in genomic regions where the percentage of heterozygous genotypes is reduced to approximately zero (Fig. 3b; Supplementary Fig. 2). In some cases, ROH are found in tracts spanning the length of the longest scaffolds in the genome assembly and therefore can exceed 1 Mb (Supplementary Fig. 2). Inferring ROH lengths is constrained by the quality of the draft assembly^12^, but the presence of long ROH implies that homozygous regions may be prominent features of some cultivars. This pattern of identity-by-descent may reflect a history of inbreeding in some varieties due to differences in the intensity of breeding, or conscious selection, for desired traits.

### Geographic selection in date palms

Strong population subdivision between North African and Middle Eastern/South Asian populations provides an opportunity to find genes associated with geographic adaptation by identifying genomic regions with reduced levels of nucleotide diversity in one population compared with the other^36^,^37^,^38^. We separately estimated *θ*_W_ in 5 kb windows for varieties from Africa and from the Middle East/South Asia, and for each interval calculated the ratio of diversity in the two populations.

We constructed a empirical distribution of this ratio (Supplementary Fig. 3), and identified outliers in the tails of a *Z*-score transformed distribution as regions that may harbour candidate adaptive genes^36^,^37^,^38^. By using this method, and applying the additional criteria that the *Z*-scores for two 5-kb windows within 10 kb of each other had to both be in the tail of the distribution (see Methods section), we identified ∼36 genomic regions that may have been subject to positive selection in the Middle East, and 20 regions in North Africa (Supplementary Tables 7 and 8). The sizes of these putative selective sweep regions range from ∼10–70 kb. We also estimated Tajima's D for each of these 5-kb windows across the genome, and find that genomic regions with outlier *Z*-scores 3 or more s.d. from the mean are enriched for negative Tajima's D value, consistent with recent selective sweeps in these regions (*χ*^2^ test, *P*<0.0001 for Middle East and *P*<0.0175 for North Africa; Supplementary Table 9).

**Figure 4:**
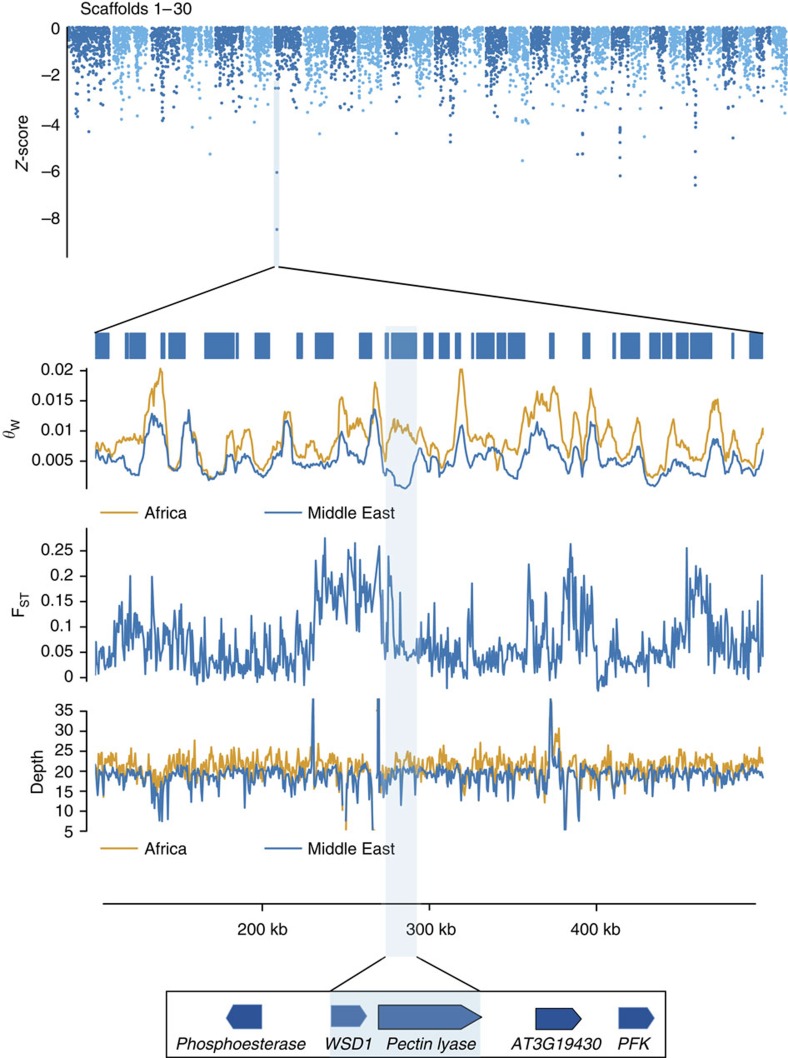
Identification of candidate selective sweep regions. The highlighted region on scaffold S000007 shows a reduction in diversity in samples from the Middle East in a region containing a pectin lyase gene, which is a candidate fruit ripening locus. Shown are the *Z*-score transformation of log_2_(*θ*_Middle East_/*θ*_North Africa_) in 5-kb windows across the longest 30 scaffolds in the genome assembly. Points from the negative half of the *Z*-score distribution are shown. *θ*_w_ and F_ST_ tracks show estimates from 5 kb and 500-bp windows. The *θ*_W_ track shows estimates in sliding windows of 5 kb with step size of 500 bp. The F_ST_ track shows estimates in 500-bp non-overlapping windows.

As an example, one outlier region on scaffold S000007 is depleted of nucleotide variation and has a ∼93% reduction in nucleotide diversity in Middle Eastern varieties relative to North African cultivars (*θ*_North Africa_=0.01027 versus *θ*_Middle East_=0.00077, Z=−8.088)^5^. Consistent with a history of recent positive selection, this region has a site-frequency spectrum skewed towards low frequency alleles (Tajima's D=−2.2780) in Middle Eastern/South Asian varieties, which is in the lower 0.1% of values in a genome-wide scan. This area of low diversity spans ∼10 kb, and includes a gene (*KacstDP.mRNA.S000007.21*) that encodes a pectin lyase (Fig. 4). This gene is implicated in cell wall degradation and fruit softening^39^, and points to geographic selection for differing fruit ripening characteristics.

Dates can be classified as being dry, semi-dry or soft when ripe. To examine whether fruit texture has a geographic association consistent with the selective sweep at the pectin lyase locus, we compiled published data on the fruit characteristics of 107 date varieties^8^,^40^. We find that North African varieties (*n*=64) are more variable in texture, with ∼52% of varieties producing soft dates and ∼31% dry. In contrast, a sample of Middle Eastern and South Asian date varieties (*n*=43) indicated that varieties producing soft dates account for ∼77% of the sample while dry date-producing varieties account for ∼7%. This predominance of soft dates in Middle Eastern/South Asian varieties is statistically significant (Fisher's exact test, *P*<0.0026), and is consistent with selection at the pectin lyase locus in Middle Eastern/South Asian varieties associated with cultural selection for fruit texture. Further studies can provide clearer mechanistic connections between specific mutations at this locus and fruit characteristics.

### Candidate loss-of-function polymorphisms

**Figure 5:**
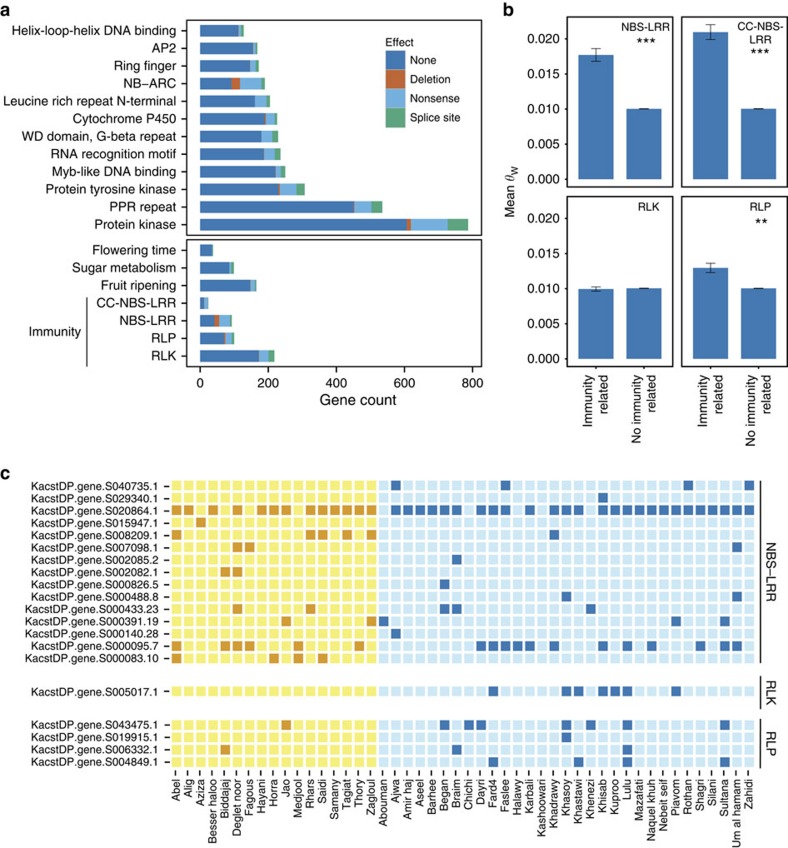
Variation in selected genes and pathways of interest in date palm. (**a**) Major effect mutations in selected pathways and large gene families in the date palm genome. (**b**) Evidence of elevated neutral polymorphism in regions containing *NBS–LRR*, *CC–NBS–LRR* and *RLP* genes consistent with long-term balancing selection acting on these gene families. Bars represent mean *θ*_W_ in 10-kb windows that contain at least one putative disease resistance gene in the indicated class compared with windows without these genes. *P* values are based on a two-tailed Wilcoxon rank-sum test (***P<10^−10^; **P<10^−5^). (**c**) Date palm cultivars with predicted deletion alleles at resistance/immunity-related gene loci in African (dark orange) and Middle Eastern (dark blue) samples. Samples without deletions are colored light yellow and light blue.

Major effect mutations, including gene deletions and nonsense polymorphisms that pre-maturely truncate encoded proteins, are classes of mutation predicted to impair gene function and represent the best candidates for functional variation among cultivars. We characterized candidate mutations in pathways associated with important agronomic traits including disease resistance, fruit ripening, fruit colour, flowering time and sugar metabolism (Fig. 5a). In total, we discovered 4,162 nonsense polymorphisms affecting 3,288 genes (excluding TE-related genes). Of these genes, 2,720 (65%) truncate the protein by at least 25% and are thus good candidates for loss-of-function alleles. Predicted major effect mutations including splice site and nonsense polymorphisms are dependent on the quality of the genome annotation and lower quality annotations are expected to inflate the number of genes impacted by these classes of damaging mutation. A comparable study of *Arabidopsis thaliana*, which has a well-curated structural annotation, found more than 4,000 genes affected by nonsense mutations in a set of 80 re-sequenced accessions^41^, which is comparable to the number in date palm.

In addition to characterizing major effect classes of SNPs, we identified cases of presence/absence variation in gene content among cultivars. By using a coverage criterion (see Methods section) to characterize gene deletions, we predicted 1,402 gene loss events. Many of these gene losses (27%) were found in only one cultivar, while ∼25% were found at moderate to high frequency (>20% of cultivars).

Both nonsense polymorphisms and gene deletions segregate in members of almost all large gene families and functional pathways we examined (Fig. 5a; Supplementary Data 1 for genes assigned to pathways). We find that only a small fraction of candidate flowering time (8%), sugar metabolism (13%) and fruit ripening (10%) genes are segregating for putative nonsense or splice site mutations that could affect gene function (Fig. 5a). Moreover, large gene families in the date palm genome including helix-loop-helix and myb-like DNA-binding proteins, cytochrome P450s and protein kinases, have putative nonsense mutations in ∼13% of the genes in these families (Fig. 5a). The fraction of observed gene losses is smaller, with a mean of ∼1.4% of genes segregating for a deletion in these families.

### Variation in candidate disease resistance genes

Disease resistance is an important agronomic trait in date palms where crop yields have been devastated by pathogens including the fungus *Fusarium oxysporum* responsible for Bayoud disease^42^. Resistance genes including the nucleotide-binding site/leucine rich repeat (NBS–LRR)^43^, receptor-like kinases (RLKs)^44^ and receptor-like proteases (RLPs)^45^ are conserved members of the plant innate immunity system and represent excellent candidates for variation in susceptibility to disease observed in date palm^46^. We evaluated if these conserved gene classes show evolutionary genetic patterns consistent with their presumed role in the immune response. Regions with CC–NBS–LRR (24 genes) and NBS–LRR genes (95 genes) are more polymorphic than other genomic regions (Fig. 5b; *P*_CC–NBS–LRR_<1.0 × 10^−16^; *P*_NBS–LRR_<1.0 × 10^−21^), consistent with long-term balancing selection acting on pathogen resistance loci as reported in other plants (Fig. 5b)^47^. RLPs, while not as diverse as the NBS–LRR containing proteins, also show significantly elevated nucleotide diversity (*P*<1.0 × 10^−7^), while RLKs are not found in unusually polymorphic regions of the date palm genome (Fig. 5b).

In addition to elevated levels of diversity, all classes of date palm resistance genes (with the exception of the NBS–LRR class) have a significant skew in the site-frequency spectrum towards higher frequency alleles as evidenced by more positive Tajima's D estimates compared with the genome-wide mean of D=−0.272 (Tajima's D_*NBS*–*LRR*_=−0.291 (*P*=0.545); D_*CC*–*NBS*–*LRR*_=−0.003 (*P*=0.00733); D_*RLK*_=−0.145 (*P*=0.00019); D_*RLP*_=0.0003 (*P*=4.7 × 10^−5^); Supplementary Fig. 4). Moreover, date palm varieties in our sample are frequently polymorphic for gene content in these gene classes (Fig. 5c). Together, these observations suggest that these classes of genes are evolving in a manner similar to what has been observed in disease resistance genes in other plant species, and are therefore candidates for variety-specific disease resistance reported in *P. dactylifera*^46^.

### Fruit colour polymorphism and the *virescens* gene

**Figure 6:**
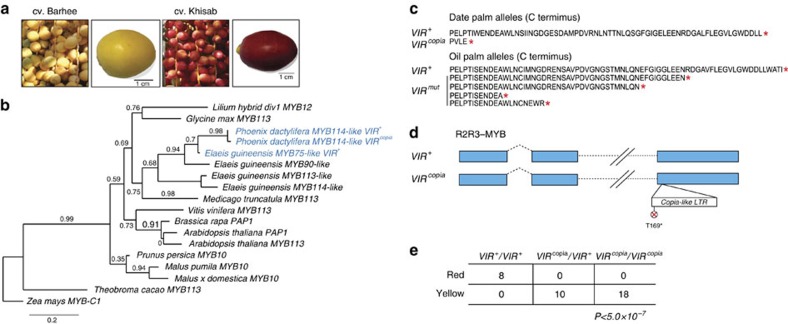
Fruit colour polymorphism in date palm is likely controlled by a dominant negative mutation in an R2R3 myb-like transcription factor. (**a**) *Khalal* stage fruits in cv. Barhee and cv. Khisab illustrating the red/yellow fruit colour polymorphism in date palm, respectively. (**b**) Phylogeny of selected members of the R2R3 myb-like transcription factor family. The analysis supports the orthology of date palm and oil palm *VIR* genes. (**c**) Alignment of the C-terminal end of the *VIR* alleles in date palm and the orthologous gene in oil palm^48^. (**d**) Representation of *VIR*^+^ and *VIR*^*copia*^ alleles found at the date palm orthologue of the oil palm R2R3 myb-like *VIR* gene, defined based on a pre-mature stop codon in exon 3 that truncates the protein in the cv. Khalas reference genome relative to the wild-type (*VIR*^+^) allele. The stop codon is introduced by a *copia*-like retrotransposon element insertion. (**e**) Genotype counts based on the alleles in (**d**) showing perfect concordance between *VIR* genotype and the fruit colour phenotype. Yellow represents a range of intermediate colours between yellow and red, including golden yellow and orange yellow (Supplementary Table 10). Samples with missing genotypes or whose fruit colour cannot be verified were excluded. This suggests that yellow fruit colour in both oil palm and date palm is caused by truncated alleles that act as dominant negative mutations.

Date palm varieties are valued for a diversity of fruit characteristics including colour, sugar composition, and texture. Fruit colour is central to varietal identity in date palms where *khalal* stage fruit varies in colour from dark red to light yellow (Fig. 6a). In the related oil palm *Elaeis guineensis*, the red and yellow fruit colour polymorphism is controlled by the *virescens* (*VIR)* gene, which encodes an R2R3 myb-like transcription factor^48^. Nonsense alleles of oil palm *VIR* act as dominant negative mutations that suppress the production of anthocyanins present in the epicarp of red fruit and thus leads to a yellow-coloured fruit.

We identified the date palm ortholog of *VIR* and present phylogenetic analysis that indicates it is most closely related to the *VIR* gene in oil palm (Fig. 6b). Interestingly, we observed that the *VIR* allele present in the cv. Khalas reference genome (NCBI Gene ID *LOC103717680*) has a *copia*-like long terminal repeat (LTR) retrotransposon insertion in the third exon, which truncates the *VIR*^*copia*^ allele relative to the oil palm gene (Fig. 6c,d; Supplementary Fig. 5). This is intriguing as cv. Khalas has yellow fruit and truncated alleles of *VIR* are responsible for yellow fruit colour in oil palm^48^.

By using a combination of approaches, we successfully designed PCR primers and amplified across the *copia*-like insertion and confirmed the existence of a 397-bp insertion in the cDNA isolated from the retrotransposon insertion allele *VIR*^*copia*^ of yellow-fruited cultivars. (Fig. 6d). This *VIR*^*copia*^ allele contains a T169* mutation located near the 5′ end of the *copia* LTR insertion, which truncates the wild-type (*VIR*^*+*^) protein by 62 amino acids (∼30% of the protein) that spans a transcriptional activation domain of the R2R3 myb-like transcription factor. By using a similar PCR-based approach, we successfully sequenced a complete *VIR*^*+*^ allele from both cDNA and genomic DNA from a cultivar with red fruit. *VIR*^*+*^ contains an open reading frame that is homologous to wild-type *VIR* in oil palm and includes an intact exon 3 (Fig. 6c,d).

We genotyped the data palm *VIR* orthologue in 36 varieties for which we had fruit colour information (Supplementary Table 10). We find that varieties that produce red fruit are found exclusively as *VIR*^*+*^/*VIR*^*+*^ homozygotes (*n*=8), while yellow-fruited varieties are either heterozygous (*VIR*^*+*^/*VIR*^*copia*^; *n*=10) or homozygous (*VIR*^*copia*^/*VIR*^*copia*^; *n*=18) for the *copia*-like insertion allele (*P*<5.0 × 10^−7^; Fig. 6e). This association between genotype at *VIR* and fruit colour phenotype suggests that *VIR* controls fruit colour in date palms and the pattern of dominance is consistent with *VIR*^*copia*^ acting as a dominant negative inhibitor of anthocyanin production comparable to the nonsense alleles reported in oil palm^48^. The genetic pattern we observe in date palm therefore parallels what has been observed in oil palms, where *VIR* acts monogenically to control fruit colour. Together, these results provide evidence that we have likely identified a causal allele for fruit colour polymorphism in date palms, and that yellow fruit colour in date and oil palms represents a genetic parallelism in this trait.

## Discussion

We report a comprehensive catalogue of genome-wide polymorphism in date palms, one of the most culturally and economically important crops of the Middle East and North Africa. The re-sequencing data provides essential information on the population structure, evolutionary history, and diversification of *P. dactylifera*, offers markers for varietal identification and GWAS approaches to mapping agriculturally important genes, and yields candidate mutations that present new opportunities for crop improvement.

Discovery of extensive variation within date palms and evidence of genetic differentiation between regional populations will be an important factor in structured association mapping^28^, as well as planning future breeding programs that capture the full range of diversity in date palm. In addition to discovery of extensive diversity, the relative rapid decay of LD in date palms suggests that GWAS should enable high-resolution mapping of genes associated with natural phenotypic variation. Interestingly, there is evidence for greater levels of inbreeding in some date palm cultivars despite the outcrossing nature of this species, which may allow for homozygosity mapping of recessive genes^49^,^50^.

Our finding that North African and Middle Eastern/South Asian date palm varieties are genetically distinct, as well as the late appearance of date palms in the North African archaeological data^18^,^19^, may indicate that date palms were domesticated in the Middle East with the rise of oasis agriculture^15^,^16^,^17^ and subsequently spread westward. We find, however, higher nucleotide diversity in North Africa, suggesting that this population is not the product of recent colonization and founder bottleneck following a hypothesized spread of date palms from a centre of origin in the Middle East.

We are unable to establish a clear understanding of the domestication history of date palms given that wild ancestral populations have never been identified^14^. Nevertheless, the evidence of geographic structure between regional North African and Middle Eastern populations coupled with the known archaeological record can be explained in two ways. One is that this species has arisen from two domestication events, one in the Middle East and a second late domestication in North Africa, possibly from separate gene pools that had diverged before the onset of domestication in either locale. Alternatively, one can posit the spread of domesticated Middle Eastern date palms and subsequent introgression with a wild or semi-cultivated population in North Africa. The latter is consistent with the elevated diversity of North African date palms, as well as the late appearance of dates in the archaeological record of the region; further detailed evolutionary demographic analysis may help discriminate between these alternative scenarios.

This catalogue of polymorphism provides a foundational resource that will assist with addressing challenges faced in date palm agriculture and developing hypotheses for genotype/phenotype relationships for trait diversity. For example, we have identified possible selective sweeps within *P. dactylifera* that promote geographic diversification. Fifty-six regions of the genome show a pattern consistent with independent histories of selection in African and Middle Eastern populations. Several of these regions harbour genes associated with fruit traits or response to abiotic stress, and provide material for further genetic studies of phenotypic diversification in this crop.

Aside from documenting the nature of perennial crop diversification, this study offers the possibility of marker-assisted selection, targeted breeding for specific traits, and discovery of candidate mutations in pathways associated with important agronomic traits^7^. Evidence that fruit colour variation in date and oil palms arise from mutations in the same gene suggests that, despite the evolutionary divergence between these two species, it may be possible to share genetic and molecular information across them to facilitate crop improvement. The availability of genome-wide SNPs, and the growing availability of comparative genome resources in plants, provides the ability to accelerate efforts to both uncover the nature of genetic and phenotypic diversity, but also to apply this to agronomic improvement in date palm^7^.

## Methods

### Sample collection and library preparation

Samples were obtained from either leaf or fruit tissue from 61 female and 1 male (cv. Fard4) cultivar of *P. dactylifera* from sources around the Middle East, North Africa, South Asia and the United States (Supplementary Table 1). Samples from the United States have a well-documented history of transplanting from locations in North Africa and the Middle East and their origins can therefore be traced to their original source^8^ country listed in Supplementary Table 1. DNA was extracted using plant DNeasy mini kit (Qiagen, Venlo, Netherlands) DNA extraction columns, and libraries prepared using Illumina TruSeq or Nextera (San Diego, CA) kits; 2 × 100 paired-end sequencing was conducted on an Illumina HiSeq 2500 sequencer (San Diego, CA) at the Center for Genomics and Systems Biology, New York University Abu Dhabi with one to four libraries per lane.

### Read alignment and SNP calling

Reads passing Illumina's quality control filter were aligned using Burroughs-Wheeler Aligner (v0.6.2)^51^ to the cv. Khalas reference genome^12^ that was modified to include the 158,462 bp chloroplast genome (Genbank GU811709.2)^52^. This modified assembly consisted of 558,181,296 bp in 82,355 scaffolds. Alignments for each sample were processed by removing duplicate reads using Picard-tools (version 1.82) MarkDuplicates and then merged using Picard-tools MergeSamFiles. Reads in insertion/deletion (indel) regions were then globally realigned using the Genome Analysis Toolkit (GATK) RealignerTargetCreator/IndelRealigner protocol (version 2.7–4)^21^,^22^.

SNP calling was performed using the GATK Unified Genotyper configured for diploid genomes. Sequencing reads with mapping quality of zero and low base quality were filtered before SNP calling per the GATK default settings. Base qualities were capped at the mapping quality of the read and bases close to indels adjusted downward during the SNP-calling step using the base alignment quality method to reduce false positives near indels^53^. Improperly paired reads were removed by applying the BadMateFilter per the Unified Genotyper default settings. SNPs were filtered to remove variants with total depth across samples of <345 (that is, less than an average of 5 × per sample) and depth >2,836 (that is, approximately twice the mean depth at variant sites), those with more than 5% missing genotypes and those found in repetitive sequences identified as low-complexity regions^23^ by mdust (ftp://occams.dfci.harvard.edu/pub/bio/tgi/software/seqclean/) or as TEs by REPET^24^. Three additional filters (MQRankSum<−42, ReadPosRankSum<−10, BaseQRankSum<−7)^21^,^22^ were applied based on low transition/transversion ratio suggesting enrichment for false positives below the cutoff thresholds.

SNP effects were assigned to each SNP using snpEff (version 2.0.5)^54^ based on gene models from the cv. Khalas reference genome^12^ and multiple effects collapsed to the most damaging effect using GATK VariantAnnotator^21^,^22^. Codons with two or three SNPs in the same codon were annotated separately as multiple-substitution codons and not considered further owing to potential mis-assignment of the effect by snpEff. All SNP effect classifications are dependent on published date palm gene models^12^ and future refinement of gene models may alter predicted effects. Raw read alignments and SNP/Indel polymorphisms in the re-sequenced genomes can be visualized in JBrowse^55^ at http://jbrowsephoenix.abudhabi.nyu.edu.

### Gene deletions

Homozygous gene deletions were inferred using a coverage breadth criterion. Gene models in which one or more samples were covered by at least one read at fewer than 15% of sites were considered as candidates for gene deletions. To reduce false positives associated with regions refractory to read mapping, inferred gene deletions were also required to be covered at 90% or more of sites in at least one re-sequenced sample. Samples with lower than 15 × coverage (Supplementary Table 2) were excluded from the gene deletion analysis.

### Gene expression in fruit

RNA-seq data from fruit was used to confirm that candidate genes (for example, *VIR*) are actively transcribed in the fruit. Gene expression data (FPKM) from fruit was obtained by mapping publicly available RNA-seq data^56^ to the reference assembly with Tophat (v2.0.6) using the -G and --no-novel-juncs arguments^57^. Read counts for each genome feature were determined using HTseq (v0.5.3p9; http://www-huber.embl.de/users/anders/HTSeq).

### Population structure

PC analysis^31^ and STRUCTURE^33^ analyses were conducted on a reduced SNP data set. Given that most scaffolds in the draft assembly^12^ have unknown physical locations and the linkage relationships of most SNPs are therefore unknown, we randomly selected ∼25 000 SNPs to reduce the impact of LD on the clustering results^33^. PC was conducted on the reduced data set with SNPRelate^31^.

STRUCTURE (version 2.3.4)^33^ was implemented using the admixture and no linkage models with a burnin length of 100,000 and 200,000 MCMC replicates following the burnin phase. The analysis was repeated 14 times for each value of *K*=1–10 and the program CLUMPP (version 1.1.2)^58^ used to permute the clusters generated from independent STRUCTURE runs. Analysis was run with the admixture model allowing for correlated allele frequencies among populations (Supplementary Table 5). *K*=2 was chosen as the best fit for the data based on a higher Δ*K*^34^ as calculated by Structure Harvester (v0.6.93)^59^.

### Genetic distance estimation

Neighbour-joining trees were constructed for the 62 *P. dactylifera* genome sequences using sites passing quality control filters (see above). Distances were calculated using a custom perl script that applies the following equation^60^ to the filtered whole genome SNP data:


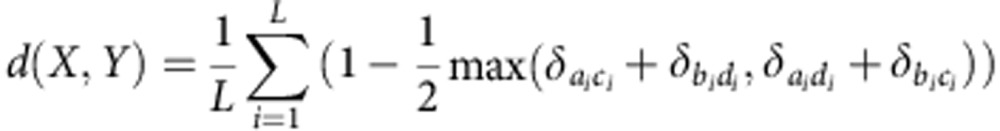


where *X* and *Y* represent two sample genomes, *L* is the total number of variable (that is, SNP) sites, *a*_*i*_ and *b*_*i*_ are the two allele copies in sample *X*, *c*_*i*_ and *d*_*i*_ are the two alleles copies in sample *Y*. Trees were then constructed from the distance matrix using the neighbour-joining method implemented in MEGA v6.06 (ref. 61).

### Population genetic parameter estimation

Population genetic statistics *θ*_W_, π, and Tajima's D were calculated directly from short-read alignments using ANGSD with non-overlapping 10 kb intervals (version 0.609)^62^. Regional estimates of *θ*_W_ and π are based African (*n*=10) or Middle Eastern (*n*=42) samples with little or no evidence of admixture in the STRUCTURE^33^ analysis. Single genome-based estimates of the population mutation rate *θ* were obtained directly from each of the sample alignments using mlRho (version 2.7)^35^; (Supplementary Table 6). Per-individual inbreeding coefficients were estimated using ngsF^63^ with genotype likelihoods generated by ANGSD^62^ provided as input. Differences in *θ*_W_ and Tajima's D between genomic intervals with and without *R* genes were tested with a two-tailed Wilcoxon rank-sum test. F_ST_ was calculated for non-overlapping genomic intervals in 500 bp windows using pypgen version 0.2.1 (https://pypi.python.org/pypi/pypgen).

LD was calculated using VCFtools (version 0.1.13)^64^ with the settings—geno-r2—ld-window-bp 100000—maf 0.1 to exclude SNPs with minor allele frequency <10%. The genome-wide LD decay rate (Fig. 1d) was estimated by binning *r*^2^ values in 1 kb intervals based on the physical distance between SNPs in the reference assembly and calculating the mean *r*^2^ for each interval. The analysis presented in Supplementary Fig. 1 was conducted using the same LD outputs as above, but binning *r*^2^ values into five categories based on the extent of LD. The cumulative count of SNP pairs in each category was then plotted as a function of the physical distance between SNPs in each pair. All LD analyses are based on the 390 scaffolds in the genome assembly that exceed 100 kb in length.

### Analysis of candidate selective sweep regions

Nucleotide diversity across the genome varies due to a combination of variation in the neutral mutation rate, the effects of linked selection and stochasticity of the coalescent process. Distinguishing neutral from selective processes responsible for patterns of polymorphism is challenging and increasingly so without an appropriate outgroup. We therefore adopted a comparative approach to identify regions of the genome where one of the two subpopulations identified by STRUCTURE^33^ is depleted of nucleotide variation with respect to the other, which may indicate the presence of a selective sweep in one population. Outlier regions were identified using this empirical distribution approach by calculating the log-transformed ratio of *θ*_W_ estimated for African (*n*=10) and Middle Eastern (*n*=42) for each 5 kb interval in the genome. The log-transformed ratios were then *Z*-score transformed so that differences in *θ*_W_ between geographic regions can be compared across genomic intervals with respect to s.d. from the mean as follows:


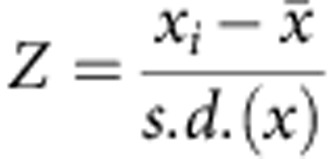


where *x*_*i*_ is log_2_(*θ*_population1_/*θ*_population2_) in each genome interval and s.d.(*x*) is the s.d. of *x*. The lower half of the *Z*-score distribution was then evaluated for both *θ*_Middle East_/*θ*_Africa_ and *θ*_Africa_/*θ*_Middle East_. Values in the lower tail of each distribution and at least five s.d. from the mean were considered outlier regions and candidates for selective sweeps.

### Heterozygosity in individual samples

The numbers of homozygous and heterozygous genotypes per sample were counted in 20 and 50 kb intervals on scaffolds with at least one complete interval. The proportion of heterozygous genotypes (that is, number heterozygote genotypes/total genotype calls) were then tabulated and density distributions and heatmaps constructed using ggplot2 (http://ggplot2.org). ROH were identified manually as extended regions (>500 kb) with zero, or near zero, heterozygosity in samples with at least 15 × coverage.

### Gene functional annotation and gene set analysis

Genes were annotated with PFAM terms using InterProScan 5 (http://www.ebi.ac.uk/Tools/pfa/iprscan5/), Gene Ontology (GO) terms using AutoFact^65^ and Kyoto Encyclopedia of Genes and Genomes (KEGG) identifiers using the KEGG Automated Annotation Server (KAAS; http://www.genome.jp/tools/kaas/). Sugar metabolism and fruit ripening gene classes were identified based on a combination of InterProScan 5 (http://www.ebi.ac.uk/Tools/pfa/iprscan5/) and homology to *A. thaliana* genes using Proteinortho V5.11 (ref. 66; (Supplementary Data 1). Candidate resistance *R* genes were identified using the PRGdb database 2.0 (ref. 67). Candidate flowering-time loci were obtained based on homology to flowering-time genes in *A. thaliana*^68^ using Proteinortho V5.11 (ref. 66). Statistical analysis was conducted in the R Statistical Programming Language (http://www.R-project.org).

### Repeats identification and annotation

Identification and annotation of repeats was performed with the REPET package^24^ on the cv. Khalas genome assembly.

### Characterization of *VIR*

The orthologue to the oil palm R2R3 myb-like transcription factor encoded by the *VIR* locus was determined using proteinortho V5.11 (ref. 66) and was found to encode a conserved protein that is expressed in date palm fruit^56^. Alignment of the orthologous protein (NCBI Gene ID *LOC103717680*) on scaffold S000271 (ref. 12) to the orthologous oil palm sequence and other R2R3 myb-like transcription factors (Supplementary Fig. 5) suggested a truncated allele in the reference genome of cv. Khalas relative to the wild-type oil palm protein. Phylogenetic analysis was conducted by downloading plant sequences identified as homologues to oil palm *VIR*^48^, aligning with MUSCLE^69^ and reconstructing the phylogeny from a 105 amino acid conserved region spanning the R2R3 domain with the Wheelan and Goldman substitution model (gamma parameter=4) in PhyML (version 3.0; Fig. 6b).

Annotation of TEs in the R2R3 myb-like gene region of the reference assembly with CENSOR (http://www.girinst.org/censor) identified a *copia*-like LTR retrotransposon that interrupts the homologous sequence in oil palm (Fig. 6b) in exon 3 of the date palm gene. The possibility of a reference genome assembly error in exon 3 of the *copia*-like insertion allele (*VIR*^*copia*^) in cv. Khalas was excluded by PCR+Sanger sequencing of genomic DNA from two varieties with yellow fruits (cv. Horra and cv. Lulu), which both confirmed the junction between the *copia*-like element and the 5′ end of exon 3. Characterization of the C-terminal end of *VIR* was complicated by the absence of sequence homologous to the oil palm *VIR* downstream of the *copia*-like element due to an apparent mis-assembly of scaffold S000271 in the vicinity of the *copia*-like element. However, we identified a scaffold (S022998) in the reference assembly with sequence homologous to exon 3 of oil palm *VIR*. Using a primer in this region paired with primers at the 5′ end of the gene on scaffold S000271, we PCR amplified and Sanger sequenced the 5′ and 3′ breakpoints between the *copia*-like insertion and the exon 3 from cDNA of a yellow-fruited variety (cv. Lulu). Inspection of the Sanger-based sequencing traces revealed a simple insertion of the element into exon 3 (Fig. 6d). Comparison of cDNA and genomic DNA sequences from the 5′ UTR through the *copia*-like insertion in cv. Lulu confirmed the existence of a stop codon at position 169 of the *VIR*^*copia*^ allele and confirmed the intron–exon structure of NCBI Gene ID *LOC103717680*, but suggest a mis-annotation of the corresponding gene (*KacstDP.gene.S000271.14*) in the annotation used throughout this study^12^. Although we were unable to amplify across the entire *copia*-like element from genomic DNA, these observations suggest that the annotated terminal stop in cv. Khalas *LOC103717680* (Supplementary Fig. 5) is introduced by a nonsense mutation in a *copia*-like element, which truncates the protein relative to wild-type *VIR* in oil palm^48^.

Inspection of the short-read alignments suggested the *copia*-like sequence in the cv. Khalas assembly was absent in cultivars that produce red fruit. To characterize wild-type (*VIR*^*+*^) in date palm, we initially used Velvet^70^ to *de novo* assemble unmapped reads from a red-fruited variety (cv. Ajwa) and confirmed the existence of a contig with an exon 3 sequence homologous to oil palm *VIR*. By using the primer downstream of the *copia*-like insertion in *VIR*^*copia*^ in a yellow-fruited cultivars (see above), we successfully PCR amplified and sequenced a complete wild-type allele from both genomic DNA and cDNA in a red-fruited variety (cv. Khenezi). This confirmed the existence of a complete open reading frame of *VIR*^*+*^ in date palm that is predicted to encode a protein 231 amino acids in length. The intron–exon structure of *VIR*^*+*^ includes two introns and three exons including a complete exon 3, which is the same as *VIR*^*copia*^ with the exception of the *copia*-like insertion in exon 3. An intact third exon was subsequently confirmed by PCR+Sanger sequencing of genomic DNA from a second cv. with red fruit (cv. Um al Blaliz). Reverse transcription PCR of cDNA from each *VIR*^*copia*^ and *VIR*^*+*^ homozygotes (cv. Lulu and cv. Khenezi) confirm that both alleles are expressed at 105 days post-pollination in *khalal* stage fruit (Supplementary Fig. 6).

Genotypes at the *VIR* locus (Supplementary Table 10) were determined through manual inspection of the short-read alignments. A 2 × 3 contingency chi-square test was conducted to test the null hypothesis of independence between genotype and *khalal* stage fruit colour (Fig. 6e).

### SNP and deletion validation

Nineteen SNPs predicted by the SNP-calling and filtering approach above were randomly chosen for validation by PCR+Sanger sequencing (Supplementary Table 3). Primers were designed flanking the focal SNP and one sample predicted to be homozygous for the reference allele and one sample predicted to be homozygous for the alternate was sequenced. Gene deletions relative to the reference assembly were validated using a PCR-based approach. Primers were designed in genes predicted to be deleted and amplifications attempted in a sample predicted to have the insertion allele and a sample predicted to be homozygous for the deletion. PCR products were then visualized using agarose gel electrophoresis (Supplementary Fig. 7).

## Additional information

**Accession codes:** The sequence data have been deposited into NCBI Sequence Read Archive (SRA) under project number PRJNA296800. Sequences for Sanger-based sequencing of the *VIR* gene have been deposited in the GenBank database under accession numbers KT734804 and KT734805.

**How to cite this article:** Hazzouri, K. M. *et al*. Whole genome re-sequencing of date palms yields insights into diversification of a fruit tree crop. *Nat. Commun.* 6:8824 doi: 10.1038/ncomms9824 (2015).

## Supplementary Material

Supplementary Information Supplementary Figures 1-7 and Supplementary Tables 1-10

Supplementary Data 1 Candidate genes in flowering time, sugar metabolism, fruit maturation, 25 and disease resistance pathways.
