# Supplementary material for: Whole genome re-sequencing of date palms yields insights into diversification of a fruit tree crop
Source: Nat Commun. 2015 Nov 9;6:8824. doi: 10.1038/ncomms9824 (PMC4667612; doi:10.1038/ncomms9824)
Supplement: Supplementary Information — Supplementary Figures 1-7 and Supplementary Tables 1-10 [file ncomms9824-s1.pdf]

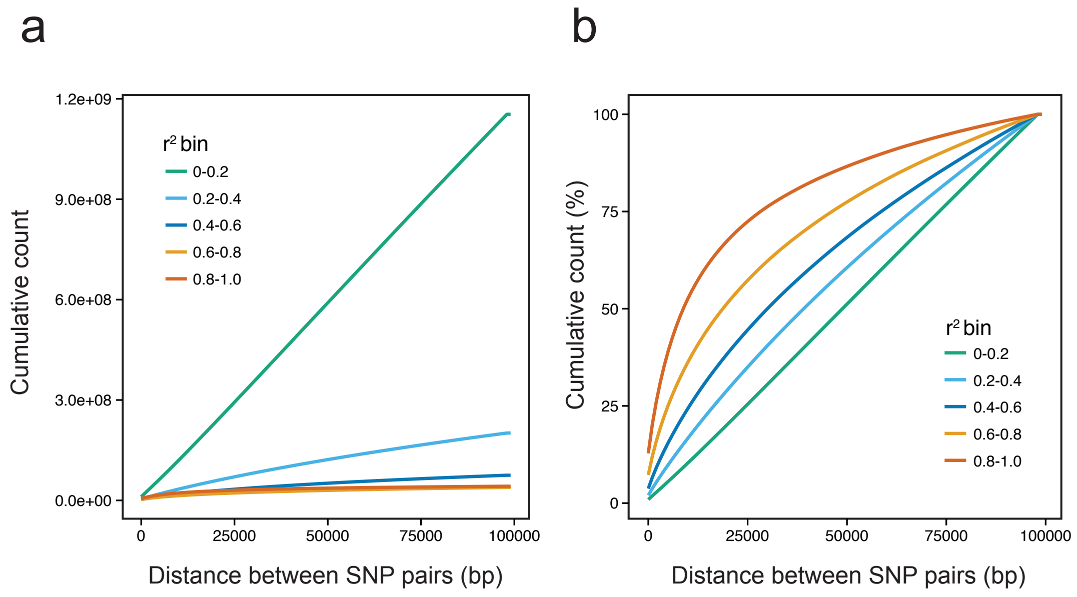

Supplementary Figure 1. Summary of linkage disequilibrium in date palm. (a) Cumulative counts of SNP pairs in r^2 bins plotted as function of the physical distance between each pair. (b) Cumulative counts of SNP pairs in r^2 bins as in (a) but plotted as a percentage of the total in each r^2 class. This illustrates the proportion of SNPs in each r^2 class that are found at different physical distances.

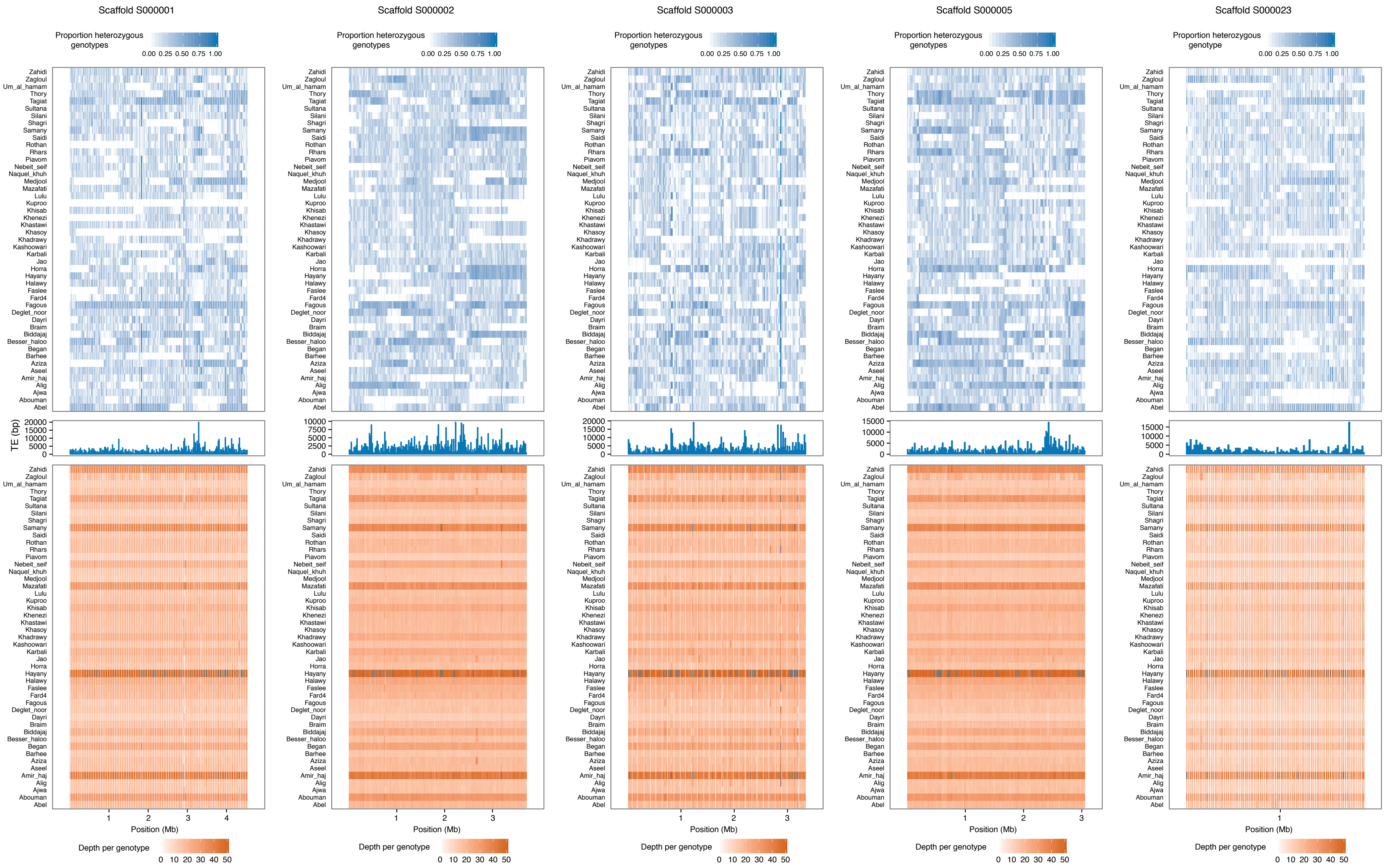

Supplementary Figure 2. Runs of homozgosity in date palm samples with 15X or higher coverage. Heatmaps in the upper panel are the proportion of called genotypes that are heterozygous in non-overlapping windows of 20 Kb. The middle panel shows the number of transposable element-related sites in each interval. The lower panel of heatmaps represents the per-genotype coverage (i.e., number of reads per sample at genotyped sites / number of called genotypes) in each interval. Gray in the coverage heatmap represents intervals where the depth per genotype exceeds 50X. Scaffolds 1-3 are the three longest scaffolds in the assembly. Scaffold 23 is also highlighted in Figure 3b. Comparison of panels shows that runs of homozygosity do not correspond to transposable element repeat content or low coverage.

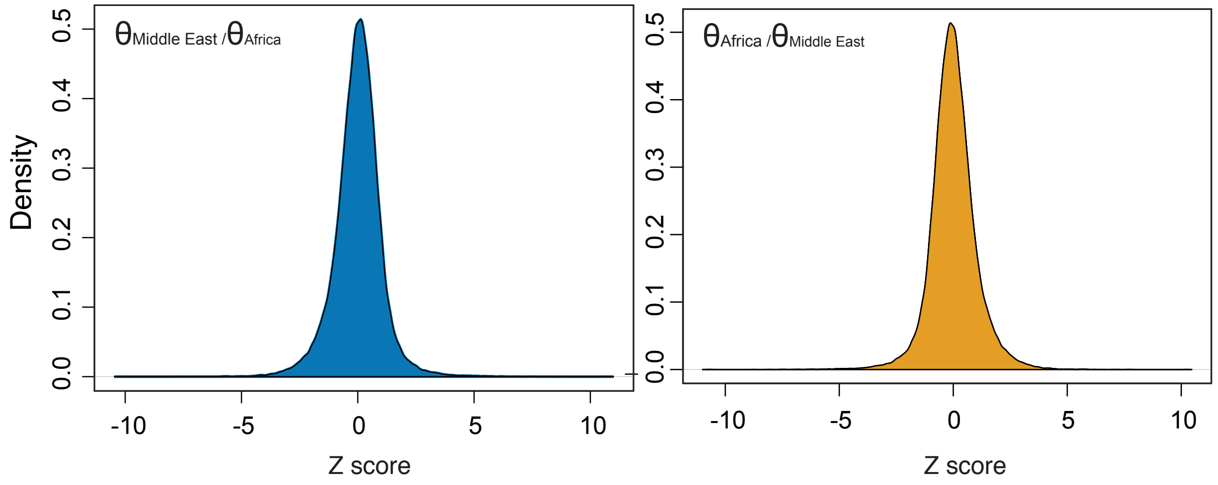

Supplementary Figure 3. Distributions of Z scores for log($\theta_{Middle\ East}/\theta_{Africa}$) and log($\theta_{Africa}/\theta_{Middle\ East}$) in genomic windows. The lower tails of each distribution were used to identify candidate sweep regions.

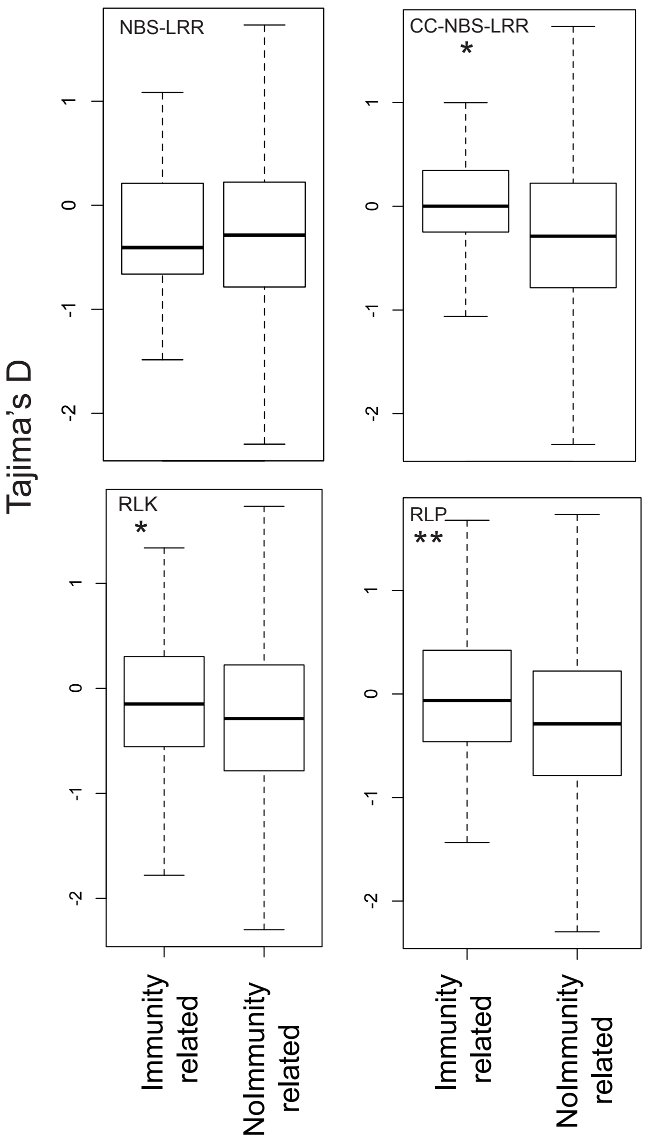

Supplementary Figure 4. Tajima’s D of disease resistance genes classes compared to the rest of the genome. *P < 0.01, **P < 0.001

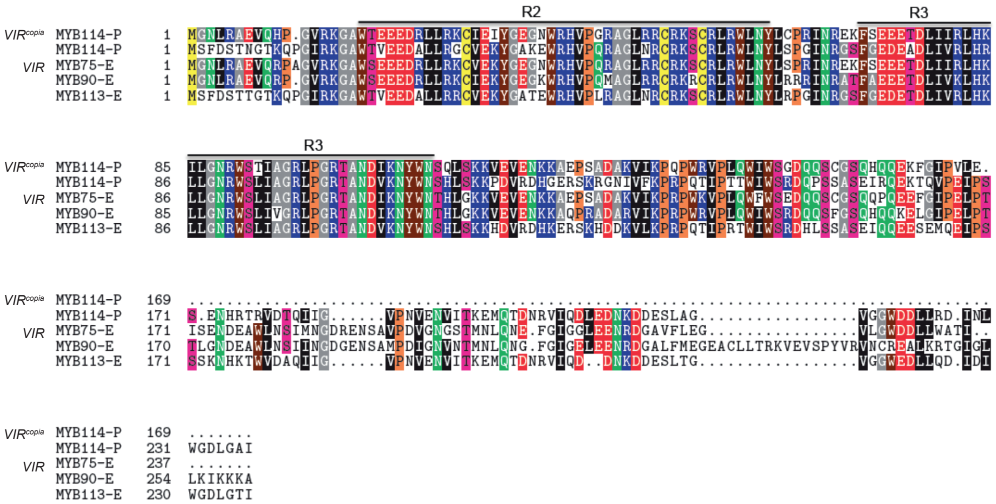

Supplementary Figure 5. Alignment of *VIR* and closely-related R2R3 myb-like transcription factors in date palm and oil palm genomes. Oil palm proteins are labeled “E” and date palm “P”. Oil palm *VIR* and date palm *VIR*^{copia} (NCBI Gene ID *LOC103717680*) are labeled. Location of R2 and R3 domains are shown. Sequences in the alignment were downloaded from NCBI. The *VIR*^{copia} protein is truncated relative to oil palm *VIR*.

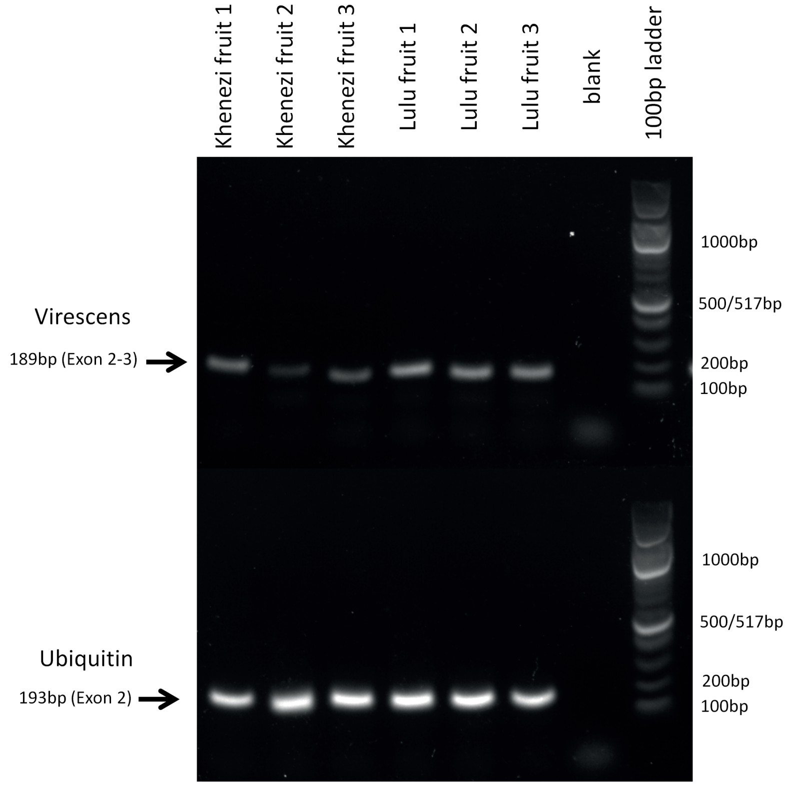

Supplementary Figure 6. RT-PCR of *VIR* from 3 replicates of red (cv. Khenezi) and yellow (cv. Lulu) *khalal* stage fruit collected at 105 days post-pollination.

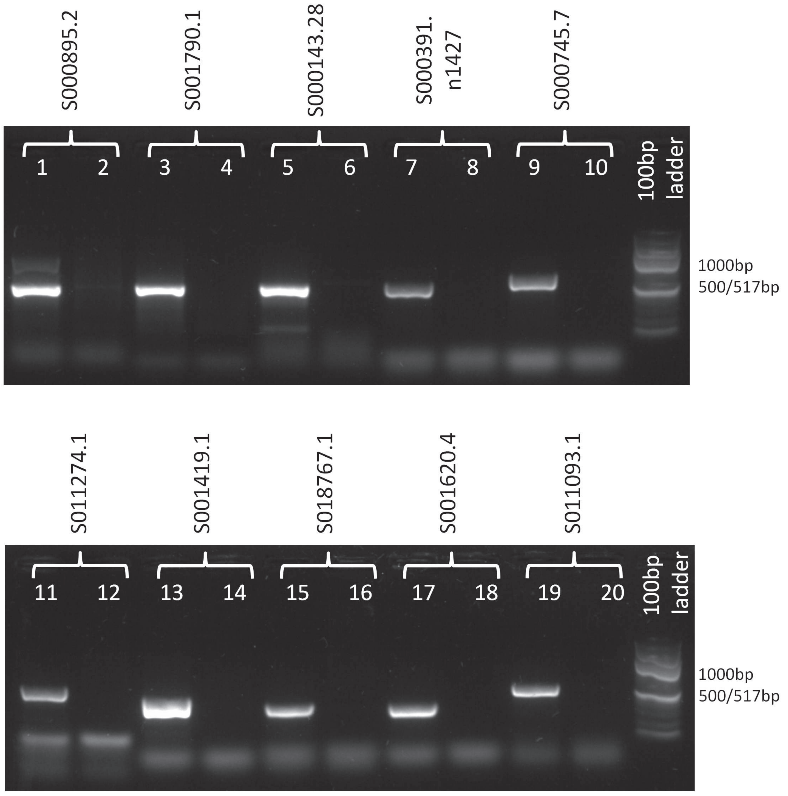

Supplementary Figure 7. Gene deletion validation. Agarose gel electrophoresis of PCR products for pairs of samples with and without a predicted gene deletion. Ten genes yielded the result expected for a gene presence/absence polymorphism and three genes failed to validate (*KacstDP.gene.S011041.1*, *KacstDP.gene.S002995.1*, *KacstDP.-gene.S000003.134*). Odd numbered lanes contain samples with a predicted insertion allele and even numbered lanes contain samples with a predicted homozygous deletion for each gene. Samples in each lanes are: (1) Chichi (2) Medjool (3) Sultana (4) Ajwa (5) Chichi (6) Thory (7) Chichi (8) Abouman (9) Chichi (10) Barhee (11) Chichi (12) Amir Haj (13) Medjool (14) Lulu (15) Chichi (16) Amir haj (17) Chichi (18) Horra (19) Chichi (20) Rothan. Gene model identifiers have been abbreviated from their standard form with the KacstDP.gene prefix.

Supplementary Table 1. Date palm cultivars, their traditional country of origin, and tissue source information.

Variety	Origin[a]	tissue	Source
Thory	Algeria	leaf	Robert Krueger (USDA[b])
Rhars	Algeria	leaf	Deborah Thirkhill (ASU[c])
Amir haj	Iraq	leaf	Robert Krueger (USDA)
Saidi	Egypt	leaf	Robert Krueger (USDA)
Zagloul	Egypt	leaf	Nadia Haider (Atomic Energy Commission[d])
Hayany	Egypt	leaf	Robert Krueger (USDA)
Samany	Egypt	leaf	Robert Krueger (USDA)
Piavom	Iran	fruit	Joel Malek (Weill Cornell Medical College[e])
Rabee	Iran	fruit	Joel Malek (Weill Cornell Medical College)
Mazafati	Iran	fruit	Joel Malek (Weill Cornell Medical College)
Kabkab red	Iran	leaf	Nadia Haider (Atomic Energy Commision)
Tagiat	Libya	fruit	Madhu Subramani (date palm festival, Libya)
Began	Pakistan	leaf	Ghulam Sarwar Markhand (DPRI[f])
Abel	Libya	fruit	Madhu Subramani (Date palm festival, Libya)
Khadrawy	Iraq	leaf	Nadia Haider (Atomic Energy Commision)
Khastawi	Iraq	leaf	Deborah Thirkhill (ASU)
Zahidi	Iraq	leaf	Robert Krueger (USDA)
Um al blaliz	Iraq	leaf	Hussam Khierallah (DPRU[g])
Um al hamam	Iraq	leaf	Hussam Khierallah (DPRU)
Sultana	Iraq	leaf	Abdul Wahab Zayed, Jorik Visser (DPTCL[h])
Azraq Azraq	Iraq	leaf	Hussam Khierallah (DPRU)
Ebrahimi	Iraq	leaf	Hussam Khierallah (DPRU)
Ewent ayob	Iraq	leaf	Hussam Khierallah (DPRU)
Silani	Iraq	leaf	Hussam Khierallah (DPRU)
Medjool	Morocco	leaf	Abdul Wahab Zayed, Jorik Visser (DPTCL)
Fagous	Morocco	fruit	Youssef Idaghdour (date palm festival, Morocco)
Biddajaj	Morocco	fruit	Youssef Idaghdour (date palm festival, Morocco)
Aziza	Morocco	fruit	Youssef Idaghdour (date palm festival, Morocco)
Braim	Oman	leaf	Deborah Thirkhill (ASU)
Kashoowari	Pakistan	leaf	Ghulam Sarwar Markhand (DPRI)

Naquel khuh	Pakistan	leaf	Ghulam Sarwar Markhand (DPRI)
Karbali	Pakistan	leaf	Ghulam Sarwar Markhand (DPRI)
Dedhi	Pakistan	leaf	Ghulam Sarwar Markhand (DPRI)
Aseel	Pakistan	leaf	Ghulam Sarwar Markhand (DPRI)
Kuproo	Pakistan	leaf	Ghulam Sarwar Markhand (DPRI)
Khasoy	Pakistan	leaf	Ghulam Sarwar Markhand (DPRI)
Chichi	Saudi Arabia	leaf	Abdul Wahab Zayed, Jorik Visser (DPTCL)
Hilali	Saudi Arabia	leaf	Robert Krueger (USDA)
Khenezi	Saudi Arabia	leaf	Abdul Wahab Zayed, Jorik Visser (DPTCL)
Nebeit seif	Saudi Arabia	leaf	Abdul Wahab Zayed, Jorik Visser (DPTCL)
Ajwa	Saudi Arabia	leaf	Khaled Masmoudi (ICBA[i])
Rothan	Saudi Arabia	leaf	Khaled Masmoudi (ICBA)
Faslee	Pakistan	leaf	Ghulam Sarwar Markhand (DPRI)
Shagri	Saudi Arabia	leaf	Khaled Masmoudi (ICBA)
Maktoumi	Iraq	leaf	Abdul Wahab Zayed, Jorik Visser (DPTCL)
Dayri	Iraq	leaf	Robert Krueger (USDA)
Barhee	Iraq	leaf	Robert Krueger (USDA)
Halawy	Iraq	leaf	Robert Krueger (USDA)
Khisab	Iraq	leaf	Robert Krueger (USDA)
Jao	Sudan	fruit	Khaled Hazzouri (date palm festival, Abu dhabi, UAE)
Alig	Tunisia	leaf	Nabila Kadri (TCD[j])
Besser heloo	Tunisia	leaf	Nabila Kadri (TCD)
Deglet noor	Algeria	leaf	Nabila Kadri (TCD)
Horra	Tunisia	leaf	Robert Krueger (USDA)
Dibbas	UAE	leaf	Abdul Wahab Zayed, Jorik Visser (DPTCL)
Helwa	UAE	leaf	Abdul Wahab Zayed, Jorik Visser (DPTCL)
Lulu	UAE	leaf	Abdul Wahab Zayed, Jorik Visser (DPTCL)
Nagal	UAE	fruit	Khaled Hazzouri (date palm festival, Abu Dhabi, UAE)
Abouman	UAE	leaf	Abdul Wahab Zayed, Jorik Visser (DPTCL)
Hiri	UAE	leaf	Abdul Wahab Zayed, Jorik Visser (DPTCL)
Fard4	UAE	leaf	Robert Krueger (USDA)
Dajwani	Iraq	leaf	Hussam Khierallah (DPRU)

[a]country of origin
[b]United States Department of Agriculture, Riverside, CA USA
[c]Arizona State University Date Palm Collection, Arizona State University Tempe, AZ USA
[d]Department of Molecular Biology and Biotechnology, Atomic Energy Commission of Syria, Damascus, Syria
[e]Genomics Core Laboratory, Weill Cornell Medical College in Qatar, Doha, Qatar
[f]Date Palm Research Institute, Sindh, Pakistan
[g]Date Palm Research Unit, College of Agriculture, University of Baghdad, Baghdad, Iraq
[h]Date Palm Tissue Culture Laboratory, United Arab Emirates University, Al-Ain, Abu Dhabi, UAE
[i]International Center for Biosaline Agriculture, Dubai, United Arab Emirates
[j]Technical Center of Dates, Ministry of Agriculture, Kebili, Tunisia

Supplementary Table 2. Sequencing, coverage statistics and the proportion of missing genotypes per sample.

Variety	Total reads	Mapped reads	Percent mapped (%)	Read length	Coverage (X)	Fraction missing genotypes
Chichi	109321274	86723531	79.3290526	93	14.44922723	0.0021
Medjool	112835840	103516262	91.740587	93	17.24710669	0.0023
Alig	131317182	119286627	90.8385523	93	19.8746471	0.0020
Besser_haloo	143153760	129533822	90.4857979	93	21.58195829	0.0035
Dibbas	64385430	57440736	89.2138734	93	9.570346564	0.0072
Kashoowari	132542886	118890896	89.6999451	93	19.80871342	0.0012
Maktoumi	94760558	82105015	86.6447146	93	13.67972458	0.0022
Naquel_khuh	128138114	112873686	88.0875194	93	18.80617081	0.0006
Piavom	101150170	91315838	90.2774934	93	15.21436314	0.0011
Rabee	87588432	72963286	83.3024229	93	12.15659795	0.0025
Helwa	93762992	79854462	85.1662903	93	13.30475424	0.0024
Hilali	41717804	36050413	86.4149345	93	6.006450651	0.0271
Hiri	37248078	30085305	80.770087	93	5.012588894	0.0539
Dedhi	79820616	71996753	90.1981927	93	11.99556144	0.0021

Saidi	133266564	119227559	89.4654709	93	19.86480569	0.0011
Khenezi	168550808	145451032	86.2950666	93	24.23396497	0.0007
Khadrawy	173379372	155991281	89.9710728	93	25.99010257	0.0004
Lulu	142356434	128516000	90.2776196	93	21.41237638	0.0007
Aseel	159213998	134491532	84.4721781	93	22.40797491	0.0010
Sultana	152396552	136799671	89.7655946	93	22.7925398	0.0007
Horra	147138214	127966502	86.97027	93	21.32082313	0.0026
Kuproo	145236312	132007111	90.891258	93	21.99403923	0.0007
Braim	141664676	129201530	91.2023616	93	21.52659427	0.0005
Deglet_noor	130029030	116134272	89.3141109	93	19.34942531	0.0015
Fard4	163481700	146318457	89.5014286	93	24.37848885	0.0009
Thory	139638242	123414451	88.381556	93	20.56239438	0.0024
Khastawi	154069874	136426128	88.5482181	93	22.73030285	0.0005
Rhars	151860304	136458378	89.8578328	93	22.73567611	0.0008
Khasoy	159291734	123975711	77.8293436	93	20.65590733	0.0012
Dayri	99972410	90514726	90.5397059	93	15.08088784	0.0021
Barhee	130291328	104754767	80.4004139	93	17.45345715	0.0012
Zahidi	281092688	238952154	85.0083137	93	39.81242382	0.0001

Amir_haj	397297466	281061091	70.7432378	93	46.8283005	0.0004
Hayany	407478644	317739714	77.9770225	93	52.93941881	0.0004
Samany	289624384	243111732	83.9403536	93	40.50546164	0.0006
Halawy	162595060	155428265	95.5922431	101	28.1239355	0.0004
Khisab	165194552	157467769	95.3226163	101	28.49297313	0.0006
Abouman	236015032	217220519	92.0367305	93	36.19166105	0.0004
Jao	155404282	128714348	82.8254835	93	21.44542365	0.0020
Abel	158159836	126359518	79.8935565	93	21.05307945	0.0028
Tagiat	250752326	190235490	75.8658925	93	31.69561699	0.0015
Karbali	189760940	152368475	80.2949622	93	25.38649768	0.0003
Zagloul	155332522	141068499	90.8171046	93	23.50378005	0.0022
Kabkab (red)	107932054	47320407	43.8427745	93	7.884172907	0.0453
Mazafati	290561316	210195293	72.3411141	93	35.02117034	0.0003
Began	211166858	174433736	82.6046936	93	29.06284672	0.0005
Faslee	168656478	145539146	86.293244	93	24.24864587	0.0006
Biddajaj	215359992	152892528	70.9939328	93	25.47381148	0.0024
Fagous	156801560	102742463	65.5238781	93	17.11818208	0.0023
Aziza	163627344	119725731	73.1697576	93	19.94780739	0.0027

Nebeit_seif	183511382	157268890	85.6998014	93	26.20296824	0.0005
Ajwa	126140454	114039697	90.4069181	93	19.00044286	0.0018
Um_al_hamam	105133034	95326359	90.6721278	93	15.8825662	0.0019
Um_al_blaliz	75838196	69893058	92.1607603	93	11.64505948	0.0023
Ewent_ayob	69893862	64821218	92.7423613	93	10.80002737	0.0027
Azraq_azraq	73139580	67832930	92.7444894	93	11.30181634	0.0021
Ebrahimi	76075150	70979772	93.3021782	93	11.82611966	0.0023
Dajwani	67058744	62476457	93.1667569	93	10.4093608	0.0038
Silani	102840866	96168201	93.51166	93	16.02282763	0.0013
Nagal	87540390	79122520	90.3840159	93	13.18280353	0.0362
Shagri	116838766	107420563	91.9391454	93	17.89761217	0.0012

Supplementary Table 3. SNP validation by PCR and Sanger sequencing.

Scaffold	Position	SNP effect	Reference Illumina	Alternative Illumina	Reference Sanger	Alternative Sanger	Reference Cultivar	Alternative Cultivar
S000092	187705	Stop Gained	C/C	A/A	C/C	A/A	Azraq azraq	Biddajaj
S000368	193868	Stop Gained	C/C	A/A	C/C	A/A	Khastawi	Rothan
S000441	11861	Stop Gained	T/T	A/A	T/T	A/A	Shagri	Horra
S000039	216873	Intron	C/C	G/G	C/C	G/G	Thory	Biddajaj
S000079	195726	Intron	A/A	G/G	A/A	G/G	Thory	Dajwani
S000161	474552	Downstream	A/A	G/G	A/A	G/G	Abouman	Um al hamam
S000188	145368	Intergenic	C/C	A/A	NA	A/A	NA	Braim
S000223	82074	Downstream	G/G	T/T	G/G	T/T	Lulu	Thory
S000253	376826	Intergenic	T/T	C/C	T/T	C/C	Azraq azraq	Dajwani
S000287	188920	Intron	C/C	T/T	C/C	T/T	Khastawi	Abouman
S000528	177537	Intron	A/A	G/G	A/A	G/G	Um al blaliz	Biddajaj
S001489	6575	Downstream	C/C	T/T	C/C	T/T	Rothan	Khastawi
S002461	10593	Intergenic	T/T	G/G	T/T	T/T	Khastawi	Dajwani
S007357	2185	Intergenic	A/A	G/G	A/A	G/G	Khastawi	Rothan
S000613	130775	Intergenic	C/C	T/T	C/C	T/T	Rothan, Biddajaj	Braim
S000641	81057	Upstream	A/A	G/G	A/A	G/G	Braim	Ebrahimi
S000648	145677	Downstream	A/A	G/G	A/A	G/G	Rabee, Dajwani	Biddajaj
S000013	1255178	Upstream	C/C	T/T	C/C	T/T	Rabee	Biddajaj
S000021	1142712	Downstream	A/A	T/T	A/A	T/T	Thory	Jao

Supplementary Table 4. Summary of nucleotide diversity in cultivated date palm. Diversity was estimated as the mean ± S.E in 5 Kb windows.
Admixed individuals identified by STRUCTURE are not considered in the estimates for each subpopulation.

	N[a]	π	θ_W	Tajima's D
All	62	0.00920 ± .00002	0.01000 ± 0.00003	-0.271 ± 0.0038
Africa	10	0.01080 ± .00003	0.009 ± 0.00003	0.401 ± 0.0033
Middle East[b]	42	0.00810 ± .00002	0.007 ± 0.00002	0.045 ± 0.0033

[a]Number of diploid genome sequences
[b]Includes samples from Pakistan

Supplementary Table 5. Summary of STRUCTURE results using the Evanno method from K=1 to K=10 based on 14 replicate runs per K.

K	Reps	MeanLnP(K)	Stdev	LnP(K)	Ln'(K)	ΔK
1	14	-1006411.371	1835.1114	NA	NA	NA
2	14	-935745.4143	74.2193	70665.95714	63572.97857	856.555898
3	14	-928652.4357	2775.7607	7092.978571	953537.1143	343.522814
4	14	-1875096.571	1756104.856	-946444.1357	581599.9786	0.331188
5	14	-2239940.729	1762370.075	-364844.1571	3242538.507	1.839874
6	14	-5847323.393	13259870.92	-3607382.664	24180944.65	1.823618
7	14	-33635650.71	20176130.98	-27788327.31	35744053.78	1.771601
8	14	-25679924.24	28894922.58	7955726.464	27306432.46	0.945025
9	14	-45030630.24	24391713.23	-19350705.99	19374411.16	0.794303
10	14	-45006925.06	27398419.21	23705.17143	NA	NA

Supplementary Table 6. Summary of statistics derived from individual genome sequences. Single genome estimates of θ are based on the method of Haubold et al. (2010) and F_{ind} estimates were obtained from ngsF (Vieira et al. 2013). F_{ind} was not obtained for admixed individuals as the estimate is based on allele frequencies in Africa or the Middle East.

Sample	θ^a	ε^b	$-\log(L)^c$	$^dF_{ind}$
Chichi	5.38e-03<5.39e-03<5.40e-03	2.88e-03<2.88e-03<2.88e-03	7.42E+08	0.004098
Medjool	7.34e-03<7.35e-03<7.36e-03	3.10e-03<3.10e-03<3.10e-03	7.81E+08	0.006179
Alig	7.38e-03<7.40e-03<7.42e-03	2.07e-03<2.07e-03<2.07e-03	1.55E+08	0.0002
Besser_haloo	7.49e-03<7.50e-03<7.51e-03	3.12e-03<3.12e-03<3.12e-03	8.12E+08	0.002925
Dibbas	5.33e-03<5.34e-03<5.35e-03	2.94e-03<2.94e-03<2.94e-03	6.30E+08	0.002772
Kashoowari	6.16e-03<6.17e-03<6.18e-03	3.09e-03<3.09e-03<3.09e-03	8.09E+08	0.021529
Maktoumi	5.65e-03<5.66e-03<5.67e-03	3.15e-03<3.15e-03<3.15e-03	7.33E+08	0.00113
Naquel_khuh	6.13e-03<6.14e-03<6.14e-03	2.86e-03<2.86e-03<2.86e-03	8.02E+08	0.000249
Piavom	6.31e-03<6.32e-03<6.33e-03	3.01e-03<3.01e-03<3.01e-03	7.61E+08	0.000122
Rabee	6.03e-03<6.04e-03<6.05e-03	2.83e-03<2.83e-03<2.83e-03	7.15E+08	NA
Helwa	5.47e-03<5.48e-03<5.49e-03	2.54e-03<2.54e-03<2.54e-03	7.14E+08	0.008344
Hilali	4.74e-03<4.75e-03<4.76e-03	2.68e-03<2.68e-03<2.68e-03	4.55E+08	0.016391
Hiri	4.64e-03<4.65e-03<4.66e-03	3.22e-03<3.22e-03<3.23e-03	3.73E+08	0.023636
Dedhi	5.41e-03<5.42e-03<5.43e-03	3.09e-03<3.09e-03<3.10e-03	7.22E+08	0.037501

Saidi	7.35e-03<7.36e-03<7.37e-03	3.45e-03<3.45e-03<3.45e-03	8.16E+08	NA
Khenezi	6.26e-03<6.29e-03<6.29e-03	3.59e-03<3.59e-03<3.59e-03	8.60E+08	0.009427
Khadrawy	6.48e-03<6.49e-03<6.50e-03	3.07e-03<3.07e-03<3.08e-03	8.69E+08	0.000468
Lulu	6.46e-03<6.46e-03<6.47e-03	3.35e-03<3.35e-03<3.35e-03	8.29E+08	0.00021
Aseel	7.88e-03<7.89e-03<7.90e-03	3.59e-03<3.59e-03<3.59e-03	8.60E+08	NA
Sultana	6.00e-03<6.01e-03<6.02e-03	3.30e-03<3.30e-03<3.30e-03	8.43E+08	0.01177
Horra	7.98e-03<7.99e-03<8.00e-03	3.32e-03<3.32e-03<3.32e-03	8.21E+08	0.009218
Kuproo	5.99e-03<6.00e-03<6.01e-03	3.36e-03<3.36e-03<3.36e-03	8.40E+08	0.053323
Braim	6.55e-03<6.56e-03<6.57e-03	3.48e-03<3.48e-03<3.48e-03	8.40E+08	0.000482
Deglet_noor	7.89e-03<7.89e-03<7.90e-03	3.19e-03<3.19e-03<3.19e-03	8.06E+08	NA
Fard4	6.22e-03<6.23e-03<6.24e-03	3.30e-03<3.30e-03<3.30e-03	8.60E+08	0.045325
Thory	7.78e-03<7.79e-03<7.80e-03	3.15e-03<3.15e-03<3.15e-03	8.08E+08	0.000188
Khastawi	6.13e-03<6.13e-03<6.14e-03	3.39e-03<3.39e-03<3.39e-03	8.44E+08	0.000478
Rhars	8.10e-03<8.11e-03<8.12e-03	3.40e-03<3.40e-03<3.40e-03	8.55E+08	NA
Khasoy	5.75e-03<5.75e-03<5.76e-03	3.38e-03<3.38e-03<3.38e-03	8.24E+08	0.03509
Dayri	6.35e-03<6.36e-03<6.37e-03	3.41e-03<3.41e-03<3.41e-03	7.62E+08	0.002744
Barhee	6.27e-03<6.27e-03<6.28e-03	3.47e-03<3.47e-03<3.47e-03	7.98E+08	0.003529
Zahidi	7.38e-03<7.39e-03<7.40e-03	2.75e-03<2.75e-03<2.75e-03	9.42E+08	0.000013

Amir_haj	7.00e-03<7.01e-03<7.02e-03	3.78e-03<3.78e-03<3.78e-03	1.04E+09	0.000179
Hayany	7.95e-03<7.96e-03<7.96e-03	3.78e-03<3.78e-03<3.78e-03	1.09E+09	NA
Samany	7.71e-03<7.72e-03<7.73e-03	3.35e-03<3.36e-03<3.36e-03	9.77E+08	NA
Halawy	6.77e-03<6.78e-03<6.79e-03	2.58e-03<2.58e-03<2.58e-03	8.56E+08	0.000316
Khisab	5.83e-03<5.90e-03<5.98e-03	2.11e-03<2.12e-03<2.13e-03	6.91E+06	0.005689
Abouman	6.51e-03<6.51e-03<6.52e-03	4.27e-03<4.27e-03<4.27e-03	1.00E+09	0.001252
Jao	6.52e-03<6.53e-03<6.54e-03	3.32e-03<3.32e-03<3.32e-03	8.31E+08	NA
Abel	7.39e-03<7.40e-03<7.41e-03	3.22e-03<3.22e-03<3.22e-03	8.18E+08	0.003502
Tagiat	8.03e-03<8.04e-03<8.04e-03	3.68e-03<3.68e-03<3.68e-03	9.35E+08	0.000012
Karbali	7.02e-03<7.03e-03<7.04e-03	3.13e-03<3.13e-03<3.13e-03	8.72E+08	0.000019
Zagloul	7.55e-03<7.55e-03<7.60e-03	3.36e-03<3.36e-03<3.36e-03	8.46E+08	NA
Kabkab red	5.17e-03<5.18e-03<5.19e-03	4.26e-03<4.26e-03<4.26e-03	5.36E+08	0.004839
Mazafati	6.96e-03<6.97e-03<6.97e-03	3.48e-03<3.48e-03<3.48e-03	9.56E+08	0.001655
Began	7.33e-03<7.34e-03<7.35e-03	3.57e-03<3.57e-03<3.57e-03	9.19E+08	NA
Faslee	6.94e-03<6.95e-03<6.96e-03	3.13e-03<3.13e-03<3.13e-03	8.61E+08	0.001396
Biddajaj	7.41e-03<7.42e-03<7.43e-03	3.84e-03<3.84e-03<3.84e-03	8.81E+08	0.004395
Fagous	8.06e-03<8.07e-03<8.08e-03	3.75e-03<3.75e-03<3.75e-03	7.99E+08	0
Aziza	7.43e-03<7.44e-03<7.45e-03	2.98e-03<2.98e-03<2.98e-03	8.03E+08	0.000083

Nebeit_seif	6.13e-03<6.14e-03<6.14e-03	3.15e-03<3.15e-03<3.15e-03	8.77E+08	0.010226
Ajwa	5.92e-03<5.93e-03<5.94e-03	2.84e-03<2.84e-03<2.84e-03	7.84E+08	0.005948
Um_al_hamam	5.86e-03<5.87e-03<5.88e-03	5.07e-03<5.07e-03<5.07e-03	8.19E+08	0.003752
Um_al_blaliz	6.05e-03<6.06e-03<6.06e-03	4.09e-03<4.09e-03<4.09e-03	7.42E+08	0.000212
Ewent_ayob	5.85e-03<5.86e-03<5.87e-03	3.81e-03<3.81e-03<3.81e-03	7.16E+08	0.000885
Azraq_azraq	5.58e-03<5.59e-03<5.60e-03	3.29e-03<3.29e-03<3.29e-03	7.14E+08	0.000816
Ebrahimi	5.81e-03<5.81e-03<5.82e-03	4.04e-03<4.04e-03<4.04e-03	7.45E+08	0.000739
Dajwani	5.39e-03<5.40e-03<5.40e-03	3.48e-03<3.48e-03<3.48e-03	6.96E+08	0.124829
Silani	6.46e-03<6.47e-03<6.47e-03	4.30e-03<4.30e-03<4.30e-03	8.12E+08	0.000163
Nagal	5.79e-03<5.80e-03<5.80e-03	4.99e-03<4.99e-03<4.99e-03	7.16E+08	0.018994
Rothan	6.54e-03<6.54e-03<6.55e-03	3.57e-03<3.57e-03<3.57e-03	8.33e+08	0.022961
Shagri	6.47e-03<6.48e-03<6.49e-03	3.50e-03<3.50e-03<3.50e-03	8.03E+08	0.011398

[a]population mutation parameter estimate with 95% confidence intervals
[b]Sequencing error rate estimate with 95% confidence intervals
[c]Negative log likelihood
[d]Inbreeding coefficient

Supplementary Table 7. Genomic regions with outlier Z scores for the statistic $\theta_{Middle\ East}/\theta_{Africa}$.

Scaffold	Start position (bp)	End position (bp)	θ, North Africa	θ, Middle East	Z, Middle East	Transcript IDa
S000007	285000	290000	0.010333459	0.000488091	-9.160032915	KacstDP.mRNA.S000007.21
S000007	280000	285000	0.010202121	0.001042352	-7.016693611	
S000013	1745000	1750000	0.007419217	0.001144429	-5.872277856	
S000013	1750000	1755000	0.006669986	0.001131341	-5.608491336	
S000016	1505000	1510000	0.01911666	0.003925687	-5.077339952	KacstDP.mRNA.S000016.98
S000016	1510000	1515000	0.01517932	0.00181823	-6.574856212	
S000016	1515000	1520000	0.014688329	0.002965084	-5.124925382	
S000019	1240000	1245000	0.013653392	0.001789733	-6.324417324	KacstDP.mRNA.S000019.94
S000019	1250000	1255000	0.01155726	0.001519166	-6.316724241	
S000019	1255000	1260000	0.012431234	0.002118321	-5.595640812	
S000019	1245000	1250000	0.01325772	0.002300245	-5.545569116	
S000021	975000	980000	0.007204438	0.001264778	-5.512888682	KacstDP.mRNA.S000021.85
S000021	980000	985000	0.011346868	0.001416247	-6.460569354	KacstDP.mRNA.S000021.86
S000021	985000	990000	0.009339509	0.001714485	-5.388821552	KacstDP.mRNA.S000021.87
S000021	990000	995000	0.016643426	0.00254856	-5.892610753	
S000021	995000	1000000	0.008089633	0.00151755	-5.328663379	
S000021	1000000	1005000	0.009887375	0.001825391	-5.37305203	
S000021	1005000	1010000	0.014940394	0.001457026	-7.146026723	
S000021	1010000	1015000	0.012791829	0.002304589	-5.440947122	
S000025	985000	990000	0.027351653	0.002608305	-7.208269652	KacstDP.mRNA.S000025.94
S000025	990000	995000	0.03204479	0.002754162	-7.497034829	KacstDP.mRNA.S000025.95
S000025	995000	1000000	0.016915233	0.00229122	-6.233320963	KacstDP.mRNA.S000025.96
S000025	1020000	1025000	0.002768142	0.000521086	-5.319024487	

S000025	1050000	1055000	0.004160301	0.000832173	-5.150357063	
S000045	340000	345000	0.006573053	0.001256989	-5.275249844	KacstDP.mRNA.S000045.30
S000045	345000	350000	0.008593463	0.001791899	-5.034855147	KacstDP.mRNA.S000045.31
S000045	350000	355000	0.006962083	0.001404903	-5.125932649	
S000056	730000	735000	0.02159655	0.003772729	-5.526596603	KacstDP.mRNA.S000056.70
S000056	735000	740000	0.008928676	0.001585158	-5.481728383	
S000056	740000	745000	0.014487533	0.002926808	-5.122781297	
S000068	795000	800000	0.013834093	0.001990454	-6.065646181	KacstDP.mRNA.S000068.46
S000068	800000	805000	0.014034309	0.00269525	-5.263456369	KacstDP.mRNA.S000068.47
S000068	805000	810000	0.009564264	0.001980785	-5.053786427	
S000078	610000	615000	0.008474436	0.00172321	-5.104693907	KacstDP.mRNA.S000078.47
S000078	620000	625000	0.009875731	0.001746217	-5.492964421	
S000080	405000	410000	0.009679794	0.001445236	-5.962843316	KacstDP.mRNA.S000080.35
S000080	410000	415000	0.009390939	0.001930863	-5.073893485	
S000117	340000	345000	0.015353947	0.001655957	-6.866338333	KacstDP.mRNA.S000117.25
S000117	350000	355000	0.012124994	0.002223995	-5.39110755	KacstDP.mRNA.S000117.26<br>KacstDP.mRNA.S000117.27
S000131	430000	435000	0.007256079	0.00146976	-5.115459452	KacstDP.mRNA.S000131.13
S000131	435000	440000	0.007316205	0.001499648	-5.082458519	
S000131	450000	455000	0.007606502	0.001563297	-5.075082828	
S000142	600000	605000	0.006809411	0.00119912	-5.504324097	KacstDP.mRNA.S000142.45
S000142	610000	615000	0.005760134	0.001127012	-5.211720181	
S000180	230000	235000	0.013583895	0.002511431	-5.369073045	KacstDP.mRNA.S000180.22
S000180	235000	240000	0.008305525	0.001413521	-5.599110101	KacstDP.mRNA.S000180.23
S000180	240000	245000	0.008475956	0.001429094	-5.625100412	
S000211	35000	40000	0.005444409	0.00070804	-6.346426088	KacstDP.mRNA.S000211.6
S000211	40000	45000	0.004355498	0.000861733	-5.180766792	

S000260	370000	375000	0.008641501	0.001740117	-5.131804052	KacstDP.mRNA.S000260.22
S000260	375000	380000	0.010981504	0.001935519	-5.50187725	
S000260	385000	390000	0.011158671	0.00172392	-5.867967404	
S000298	225000	230000	0.009925663	0.001763636	-5.479400387	KacstDP.mRNA.S000298.18
S000298	230000	235000	0.008859012	0.001398061	-5.808886109	
S000303	140000	145000	0.008544548	0.001675799	-5.205087561	KacstDP.mRNA.S000303.8
S000303	150000	155000	0.011741569	0.002449139	-5.033946918	
S000303	160000	165000	0.008577681	0.001665866	-5.232354571	
S000333	50000	55000	0.011140905	0.002265727	-5.104311978	
S000333	55000	60000	0.013891483	0.002314036	-5.658687012	
S000350	150000	155000	0.008952462	0.000931983	-6.964614322	KacstDP.mRNA.S000350.14
S000350	160000	165000	0.008312883	0.001625331	-5.213676315	
S000350	165000	170000	0.00679058	0.001375995	-5.114400384	
S000361	40000	45000	0.006267588	0.000788435	-6.438804829	KacstDP.mRNA.S000361.4
S000361	45000	50000	0.004680779	0.000833212	-5.474360097	
S000406	100000	105000	0.014871019	0.00268971	-5.430047829	KacstDP.mRNA.S000406.6
S000406	105000	110000	0.011748469	0.001187822	-7.045823247	
S000406	110000	115000	0.005530116	0.000873339	-5.806917313	
S000406	115000	120000	0.010939075	0.000908683	-7.591707773	
S000406	120000	125000	0.011095016	0.001119814	-7.050634404	
S000406	125000	130000	0.013655309	0.000799358	-8.563957038	
S000406	130000	135000	0.012368052	0.000920772	-7.896069784	
S000406	140000	145000	0.011962771	0.002447747	-5.087375858	
S000406	145000	150000	0.013158292	0.002022389	-5.882292589	
S000465	5000	10000	0.004184037	0.000490612	-6.634050451	KacstDP.mRNA.S000465.1
S000465	10000	15000	0.006677053	0.000885322	-6.29263368	
S000472	15000	20000	0.013621746	0.002344576	-5.567789478	KacstDP.mRNA.S000472.4

S000472	20000	25000	0.009762284	0.001941563	-5.16627732	
S000678	90000	95000	0.019776825	0.002413164	-6.523466525	KacstDP.mRNA.S000678.6
S000678	95000	100000	0.016679099	0.001687407	-7.044050267	
S000678	100000	105000	0.018270534	0.000910586	-9.010895796	
S000678	105000	110000	0.014414173	0.001893567	-6.318383563	
S000678	110000	115000	0.010544581	0.001505197	-6.087634483	
S000678	115000	120000	0.018546113	0.003119549	-5.631709446	
S000678	120000	125000	0.017223272	0.002752186	-5.774207672	
S000713	45000	50000	0.005743178	0.000954217	-5.665893718	KacstDP.mRNA.S000713.1
S000713	50000	55000	0.010655727	0.001812495	-5.600654544	KacstDP.mRNA.S000713.1.1
S000713	55000	60000	0.00495268	0.000962596	-5.230220559	KacstDP.mRNA.S000713.3
S000713	65000	70000	0.00585114	0.000907917	-5.855807103	
S000713	75000	80000	0.008812318	0.001540414	-5.524830694	
S000713	80000	85000	0.009167451	0.000995756	-6.846666416	
S000713	90000	95000	0.012629771	0.001197064	-7.225239503	
S000713	95000	100000	0.004842631	0.000912539	-5.316152259	
S000713	100000	105000	0.005158712	0.000931009	-5.436138348	
S000723	10000	15000	0.009929602	0.001703398	-5.577047092	KacstDP.mRNA.S000723.1
S000723	15000	20000	0.009078728	0.001534688	-5.617916965	
S000753	60000	65000	0.006916872	0.001020786	-5.99513194	KacstDP.mRNA.S000753.1
S000753	65000	70000	0.010118519	0.001953753	-5.248458085	
S000806	40000	45000	0.022277886	0.002273159	-7.020327564	KacstDP.mRNA.S000806.4
S000806	45000	50000	0.01551857	0.00300648	-5.23915782	KacstDP.mRNA.S000806.5
S000806	50000	55000	0.019591912	0.002865756	-6.019839738	
S001237	30000	35000	0.006415394	0.001167166	-5.413770976	
S001237	35000	40000	0.005428086	0.001084694	-5.153097092	
S001416	10000	15000	0.008158672	0.001364835	-5.646922318	KacstDP.mRNA.S001416.1

S001416	15000	20000	0.01173465	0.002127253	-5.423751705	KacstDP.mRNA.S001416.2
S001416	20000	25000	0.008539382	0.001361375	-5.780673416	KacstDP.mRNA.S001416.3
S001445	5000	10000	0.018782552	0.001181955	-8.36305836	KacstDP.mRNA.S001445.2
S001445	15000	20000	0.005765897	0.001127751	-5.212677211	
S001487	10000	15000	0.006506877	0.001046816	-5.755429266	KacstDP.mRNA.S001487.1
S001487	15000	20000	0.011446145	0.001764377	-5.874188146	KacstDP.mRNA.S001487.2
S001497	15000	20000	0.01207546	0.002363228	-5.211042217	KacstDP.mRNA.S001497.4
S001497	30000	35000	0.006707567	0.00094931	-6.111436527	
S001747	15000	20000	0.006484217	0.001319285	-5.103071176	
S001747	20000	25000	0.008104145	0.001488002	-5.388267167	

[a]Transcript identifiers that found within the genomic interval

Supplementary Table 8. Genomic regions with outlier Z scores for the statistic $\theta_{Africa}/\theta_{Middle\ East.}$

Scaffold	Start position (bp)	Stop position (bp)	θ, North Africa	θ, Middle East	Z, North Africa	Transcript ID[a]
S000035	1180000	1185000	0.000138604	0.00201177	-8.111340705	KacstDP.mRNA.S000035.101
S000035	1190000	1195000	0.000300968	0.005467611	-8.734858531	KacstDP.mRNA.S000035.102
						KacstDP.mRNA.S000035.103
S000046	960000	965000	0.000321926	0.002292854	-6.133590803	KacstDP.mRNA.S000046.81
S000046	970000	975000	0.000583183	0.002937346	-5.171076775	KacstDP.mRNA.S000046.82
S000051	600000	605000	0.000273355	0.00132503	-5.064561513	KacstDP.mRNA.S000051.46
S000051	605000	610000	0.00053259	0.003286079	-5.734849421	
S000125	170000	175000	0.000371492	0.002397117	-5.859296027	KacstDP.mRNA.S000125.11
S000125	175000	180000	0.000532262	0.006426701	-7.599987913	
S000145	610000	615000	0.000779263	0.00403221	-5.245974834	KacstDP.mRNA.S000145.43
S000145	615000	620000	0.000440682	0.003109052	-6.107251224	
S000153	425000	430000	0.000444999	0.002829535	-5.818461133	KacstDP.mRNA.S000153.31
S000153	430000	435000	0.000366106	0.002528329	-6.047915734	
S000178	390000	395000	0.002795035	0.014176855	-5.190538109	KacstDP.mRNA.S000178.31
S000178	405000	410000	0.000382469	0.002763374	-6.173398052	KacstDP.mRNA.S000178.34
S000178	410000	415000	0.000285305	0.001707004	-5.649390383	KacstDP.mRNA.S000178.32
S000178	440000	445000	0.000308713	0.003449833	-7.384933752	KacstDP.mRNA.S000178.33
						KacstDP.mRNA.S000178.35
S000216	150000	155000	0.000302138	0.003057022	-7.108915969	KacstDP.mRNA.S000216.14
S000216	155000	160000	0.000547364	0.004970892	-6.808685495	

S000237	60000	65000	0.000456827	0.003297798	-6.171023971	KacstDP.mRNA.S000237.5
S000237	70000	75000	0.001263912	0.006509414	-5.232963729	
S000262	135000	140000	0.0016658	0.009239133	-5.438831094	KacstDP.mRNA.S000262.13
S000262	140000	145000	0.00122317	0.00898391	-6.219039526	
S000356	190000	195000	0.001059098	0.00856635	-6.486940872	KacstDP.mRNA.S000356.17
S000356	195000	200000	0.000992718	0.00648772	-5.894647048	
S000364	220000	225000	0.001289077	0.007174415	-5.448421517	KacstDP.mRNA.S000364.17
S000364	225000	230000	0.001595254	0.007741268	-5.067654781	KacstDP.mRNA.S000364.18
S000364	230000	235000	0.001247045	0.006416759	-5.230459714	
S000364	235000	240000	0.001474231	0.007716537	-5.277944438	
S000433	20000	25000	0.000674709	0.003964882	-5.599423147	KacstDP.mRNA.S000433.2
S000433	30000	35000	0.001552555	0.00759928	-5.091598905	
S000433	65000	70000	0.001056718	0.005072052	-5.037221342	
S000433	70000	75000	0.000480505	0.005736636	-7.568623226	KacstDP.mRNA.S000433.6
S000576	95000	100000	0.000337912	0.002156099	-5.82811408	
S000576	110000	115000	0.000203219	0.00096275	-5.000917597	KacstDP.mRNA.S000576.6
S000585	40000	45000	0.000366774	0.002894125	-6.418234266	
S000585	45000	50000	0.000446989	0.002458524	-5.415606262	
S000959	50000	55000	0.000493893	0.002702981	-5.401741422	
S000959	60000	65000	0.000292709	0.002722385	-6.874925826	
S001008	25000	30000	0.003367143	0.015971442	-5.004329287	KacstDP.mRNA.S001080.2
S001008	30000	35000	0.003539565	0.017529974	-5.124260058	
S001373	35000	40000	0.004343273	0.027678999	-5.824712106	

S001373	40000	45000	0.006186785	0.03577077	-5.554323131	
S001411	10000	15000	0.000618504	0.003043144	-5.10602054	KacstDP.mRNA.S001411.2
S001411	15000	20000	0.000385184	0.002385274	-5.744988488	
S001411	20000	25000	0.000572589	0.00352272	-5.726854377	
S001479	30000	35000	0.000716288	0.003650154	-5.203528972	KacstDP.mRNA.S001479.5
S001479	35000	40000	0.000636582	0.006249991	-7.025322595	

[a]Transcript identifiers that found within the genomic interval

Supplementary Table 9. Test for homogeneity of positive and negative Tajima's D values in candidate sweep regions versus the remainder of the genome. Candidate sweep regions are here represented as intervals in the tails of the Z-score distribution of $\log(\theta_{Middle\ East}/\theta_{Africa})$ and $\log(\theta_{Africa}/\theta_{Middle\ East})$, where the tail is defined as intervals 3 standard deviations from the mean for the lower tail of the distribution. Tests are a two-tailed chi-square 2 X 2 contingency table with Yates correction.

$\theta_{Middle\ East}/\theta_{Africa}$	*D* (-)	*D* (+)	Total
Tail	459	161	620
Genome	41960	43754	85714
Total	42419	43915	86334

Chi-square = 153.903, *df* = 1, *P* < 0.0001

$\theta_{Africa}/\theta_{Middle\ East}$	*D* (-)	*D* (+)	Total
Tail	235	389	624
Genome	28358	57352	85710
Total	28593	57741	86334

Chi-square = 5.647, *df* = 1, *P* < 0.0175

Supplementary Table 10. Summary of fruit color and *VIR* genotype of date palm varieties.

Variety	Color	Genotype
Abel	NA	VIR^{copia}/VIR^{copia}
Abouman	Yellow	VIR^{copia}/VIR^{copia}
Ajwa	Red	VIR^{+}/VIR^{+}
Alig	NA	VIR^{copia}/VIR^{+}
Amir_haj	Yellow	VIR^{copia}/VIR^{+}
Aseel	NA	VIR^{copia}/VIR^{+}
Aziza	NA	VIR^{copia}/VIR^{copia}
Azraq_Azraq	Yellow	VIR^{copia}/VIR^{+}
Barhee	Yellow	VIR^{copia}/VIR^{copia}
Began	NA	VIR^{+}/VIR^{+}
Besser_heloo	NA	VIR^{copia}/VIR^{+}
Biddajaj	NA	VIR^{copia}/VIR^{copia}
Braim	Yellow with fine red stippling	VIR^{copia}/VIR^{+}
Chichi	Yellow	VIR^{copia}/VIR^{copia}
Dajwani	Yellow	VIR^{copia}/VIR^{copia}
Dayri	Red	$VIR^{copia}/?$
Dedhi	NA	VIR^{copia}/VIR^{copia}
Deglet_noor	NA	VIR^{copia}/VIR^{+}
Dibbas	Golden yellow	VIR^{copia}/VIR^{copia}
Ebrahimi	Yellow	VIR^{copia}/VIR^{+}
Ewent_ayob	Yellow	VIR^{copia}/VIR^{copia}
Fagous	NA	VIR^{copia}/VIR^{copia}
Fard4	NA	VIR^{+}/VIR^{+}
Faslee	NA	VIR^{copia}/VIR^{copia}
Halawy	Yellow	VIR^{copia}/VIR^{copia}
Hayany	Red	VIR^{+}/VIR^{+}
Helwa	Yellow	VIR^{copia}/VIR^{copia}
Hilali	Yellow	VIR^{copia}/VIR^{+}
Hiri	Yellow	VIR^{copia}/VIR^{copia}
Horra	Yellow	VIR^{copia}/VIR^{copia}
Jao	NA	VIR^{copia}/VIR^{copia}
Kabkab red	Red	VIR^{+}/VIR^{+}
Karbali	NA	VIR^{copia}/VIR^{copia}
Kashoowari	NA	VIR^{copia}/VIR^{+}
Khadrawy	Yellow	VIR^{copia}/VIR^{copia}
Khasoy	NA	VIR^{+}/VIR^{+}
Khastawi	Yellow	VIR^{copia}/VIR^{+}
Khenezi	Red	VIR^{+}/VIR^{+}
Khisab	Red	VIR^{+}/VIR^{+}
Kuproo	NA	VIR^{copia}/VIR^{copia}
Lulu	Yellow	VIR^{copia}/VIR^{copia}
Maktoumi	Yellow	VIR^{copia}/VIR^{copia}
Mazafati	NA	VIR^{+}/VIR^{+}
Medjool	Orange-yellow	VIR^{copia}/VIR^{+}
Nagal	NA	VIR^{copia}/VIR^{+}

Naquel_khuh	NA	VIR^{copia}/VIR^{+}
Nebeit_seif	Yellow	VIR^{copia}/VIR^{+}
P. sylvestris	NA	
Piavom	NA	VIR^{copia}/VIR^{+}
Rabee	NA	VIR^{copia}/VIR^{+}
Rhars	Yellow	VIR^{copia}/VIR^{copia}
Rothan	Yellow	
Saidi	Orange-yellow	VIR^{copia}/VIR^{+}
Samany	Yellow	VIR^{copia}/VIR^{+}
Shagri	NA	VIR^{copia}/VIR^{copia}
Silani	Yellow	VIR^{copia}/VIR^{copia}
Sultana	Yellow	VIR^{copia}/VIR^{copia}
Tagiat	NA	VIR^{copia}/VIR^{copia}
Thory	Yellow	VIR^{copia}/VIR^{copia}
Um al-blaliz	Red	VIR^{+}/VIR^{+}
Um- al-hamam	Yellow	VIR^{copia}/VIR^{copia}
Zagloul	Red	VIR^{+}/VIR^{+}
Zahidi	Yellow	VIR^{copia}/VIR^{copia}
