# Supplementary material for: Whole genome re-sequencing of date palms yields insights into diversification of a fruit tree crop
Source: Nat Commun. 2015 Nov 9;6:8824. doi: 10.1038/ncomms9824 (PMC4667612; doi:10.1038/ncomms9824)
Supplement: Supplementary Data 1 — Candidate genes in flowering time, sugar metabolism, fruit maturation, 25 and disease resistance pathways. [file ncomms9824-s2.pdf]

Supplementary Data 1. Candidate genes in flowering time, sugar metabolism, fruit maturation, and disease resistance pathways.

Gene ID	Transcript ID	Gene	Trait
KacstDP.gene.S000796.7	KacstDP.mRNA.S000796.7	PHYA	Flowering time
KacstDP.gene.S000017.102	KacstDP.mRNA.S000017.102	GAI	Flowering time
KacstDP.gene.S000018.20	KacstDP.mRNA.S000018.20	MFT	Flowering time
KacstDP.gene.S000089.34	KacstDP.mRNA.S000089.34	GI	Flowering time
KacstDP.gene.S000002.289	KacstDP.mRNA.S000002.289	GI	Flowering time
KacstDP.gene.S000070.66	KacstDP.mRNA.S000070.66	PFT1	Flowering time
KacstDP.gene.S000747.6	KacstDP.mRNA.S000747.6	FRL2	Flowering time
KacstDP.gene.S000266.17	KacstDP.mRNA.S000266.17	FT	Flowering time
KacstDP.gene.S000001.n8	KacstDP.mRNA.S000001.n8	FT	Flowering time
KacstDP.gene.S000462.4	KacstDP.mRNA.S000462.4	RGL2	Flowering time
KacstDP.gene.S000010.55	KacstDP.mRNA.S000010.55	FKF1	Flowering time
KacstDP.gene.S000013.77	KacstDP.mRNA.S000013.77	PHYB	Flowering time
KacstDP.gene.S000112.35	KacstDP.mRNA.S000112.35	FVE	Flowering time
KacstDP.gene.S000008.85	KacstDP.mRNA.S000008.85	FVE	Flowering time
KacstDP.gene.S000017.61	KacstDP.mRNA.S000017.61	SVP	Flowering time
KacstDP.gene.S000025.20	KacstDP.mRNA.S000025.20	ATC	Flowering time
KacstDP.gene.S000003.35	KacstDP.mRNA.S000003.35	ATC	Flowering time
KacstDP.gene.S000096.56	KacstDP.mRNA.S000096.56	SOC1	Flowering time
KacstDP.gene.S000004.37	KacstDP.mRNA.S000004.37	SOC1	Flowering time
KacstDP.gene.S000811.8	KacstDP.mRNA.S000811.8	FLK	Flowering time
KacstDP.gene.S000434.9	KacstDP.mRNA.S000434.9	GAr1	Flowering time
KacstDP.gene.S000108.14	KacstDP.mRNA.S000108.14	GAr1	Flowering time
KacstDP.gene.S000415.13	KacstDP.mRNA.S000415.13	SPY	Flowering time
KacstDP.gene.S000319.23	KacstDP.mRNA.S000319.23	SPY	Flowering time

KacstDP.gene.S001969.1	KacstDP.mRNA.S001969.1	GA1	Flowering time
KacstDP.gene.S000219.17	KacstDP.mRNA.S000219.17	CRY1	Flowering time
KacstDP.gene.S000067.41	KacstDP.mRNA.S000067.41.1	CRY1	Flowering time
KacstDP.gene.S000067.41	KacstDP.mRNA.S000067.41	CRY1	Flowering time
KacstDP.gene.S000637.5	KacstDP.mRNA.S000637.5	ESD4	Flowering time
KacstDP.gene.S000093.9	KacstDP.mRNA.S000093.9	EBS	Flowering time
KacstDP.gene.S000006.37	KacstDP.mRNA.S000006.37	SLY1	Flowering time
KacstDP.gene.S000251.25	KacstDP.mRNA.S000251.25	VIN3L	Flowering time
KacstDP.gene.S000038.18	KacstDP.mRNA.S000038.18	ATMYB33	Flowering time
KacstDP.gene.S000016.n262	KacstDP.mRNA.S000016.n286.1	CO	Flowering time
KacstDP.gene.S000057.72	KacstDP.mRNA.S000057.72	CO	Flowering time
KacstDP.gene.S000194.8	KacstDP.mRNA.S000194.8	FPF1	Flowering time
KacstDP.gene.S000059.n618	KacstDP.mRNA.S000059.n658	FPF1	Flowering time
KacstDP.gene.S000316.19	KacstDP.mRNA.S000316.19	CDF1	Flowering time
KacstDP.gene.S000228.12	KacstDP.mRNA.S000228.12	CDF1	Flowering time
KacstDP.gene.S000771.5	KacstDP.mRNA.S000771.5	HXK	Starch and sucrose metabolism
KacstDP.gene.S000368.13	KacstDP.mRNA.S000368.13	HXK	Starch and sucrose metabolism
KacstDP.gene.S000003.136	KacstDP.mRNA.S000003.136	HXK	Starch and sucrose metabolism
KacstDP.gene.S000005.13	KacstDP.mRNA.S000005.13	HXK	Starch and sucrose metabolism
KacstDP.gene.S002141.2	KacstDP.mRNA.S002141.2	HXK	Starch and sucrose metabolism
KacstDP.gene.S000041.6	KacstDP.mRNA.S000041.6	HXK	Starch and sucrose metabolism
KacstDP.gene.S017439.1	KacstDP.mRNA.S017439.1	HXK	Starch and sucrose metabolism
KacstDP.gene.S000101.6	KacstDP.mRNA.S000101.6	INV	Starch and sucrose metabolism
KacstDP.gene.S000088.48	KacstDP.mRNA.S000088.48	INV	Starch and sucrose metabolism
KacstDP.gene.S000022.70	KacstDP.mRNA.S000022.70	INV	Starch and sucrose metabolism
KacstDP.gene.S000014.122	KacstDP.mRNA.S000014.122	INV	Starch and sucrose metabolism
KacstDP.gene.S000003.55	KacstDP.mRNA.S000003.55	INV	Starch and sucrose metabolism

KacstDP.gene.S000199.37	KacstDP.mRNA.S000199.37	INV	Starch and sucrose metabolism
KacstDP.gene.S000013.100	KacstDP.mRNA.S000013.100	INV	Starch and sucrose metabolism
KacstDP.gene.S000102.22	KacstDP.mRNA.S000102.22	SPS	Starch and sucrose metabolism
KacstDP.gene.S000106.22	KacstDP.mRNA.S000106.22	SPS	Starch and sucrose metabolism
KacstDP.gene.S000003.204	KacstDP.mRNA.S000003.204	SPS	Starch and sucrose metabolism
KacstDP.gene.S000359.16	KacstDP.mRNA.S000359.16	SPS	Starch and sucrose metabolism
KacstDP.gene.S000036.66	KacstDP.mRNA.S000036.66	SPS	Starch and sucrose metabolism
KacstDP.gene.S000183.16	KacstDP.mRNA.S000183.16	SUS	Starch and sucrose metabolism
KacstDP.gene.S000135.48	KacstDP.mRNA.S000135.48	SUS	Starch and sucrose metabolism
KacstDP.gene.S000002.59	KacstDP.mRNA.S000002.59	SUS	Starch and sucrose metabolism
KacstDP.gene.S000059.46	KacstDP.mRNA.S000059.46	SUS	Starch and sucrose metabolism
KacstDP.gene.S000007.151	KacstDP.mRNA.S000007.151	SUS	Starch and sucrose metabolism
KacstDP.gene.S000051.43	KacstDP.mRNA.S000051.43	SUS	Starch and sucrose metabolism
KacstDP.gene.S000117.18	KacstDP.mRNA.S000117.18	SUS	Starch and sucrose metabolism
KacstDP.gene.S000028.30	KacstDP.mRNA.S000028.30	SUS	Starch and sucrose metabolism
KacstDP.gene.S000028.31	KacstDP.mRNA.S000028.31	SUS	Starch and sucrose metabolism
KacstDP.gene.S000002.n35	KacstDP.mRNA.S000002.n41	SUS	Starch and sucrose metabolism
KacstDP.gene.S000172.24	KacstDP.mRNA.S000172.24	phosphofructokinase	Starch and sucrose metabolism
KacstDP.gene.S000152.33	KacstDP.mRNA.S000152.33	phosphofructokinase	Starch and sucrose metabolism
KacstDP.gene.S000096.8	KacstDP.mRNA.S000096.8.1	phosphofructokinase	Starch and sucrose metabolism
KacstDP.gene.S000096.8	KacstDP.mRNA.S000096.8	phosphofructokinase	Starch and sucrose metabolism
KacstDP.gene.S000122.28	KacstDP.mRNA.S000122.28	phosphofructokinase	Starch and sucrose metabolism
KacstDP.gene.S003571.1	KacstDP.mRNA.S003571.1	phosphofructokinase	Starch and sucrose metabolism
KacstDP.gene.S000109.29	KacstDP.mRNA.S000109.29	phosphofructokinase	Starch and sucrose metabolism
KacstDP.gene.S000841.5	KacstDP.mRNA.S000841.5	phosphofructokinase	Starch and sucrose metabolism
KacstDP.gene.S000218.24	KacstDP.mRNA.S000218.24	phosphofructokinase	Starch and sucrose metabolism
KacstDP.gene.S000005.40	KacstDP.mRNA.S000005.40	phosphofructokinase	Starch and sucrose metabolism

KacstDP.gene.S000304.1	KacstDP.mRNA.S000304.1	phosphofructokinase	Starch and sucrose metabolism
KacstDP.gene.S000112.69	KacstDP.mRNA.S000112.69	phosphofructokinase	Starch and sucrose metabolism
KacstDP.gene.S000050.46	KacstDP.mRNA.S000050.46	phosphofructokinase	Starch and sucrose metabolism
KacstDP.gene.S002951.1	KacstDP.mRNA.S002951.1	phosphofructokinase	Starch and sucrose metabolism
KacstDP.gene.S000228.38	KacstDP.mRNA.S000228.38	phosphofructokinase	Starch and sucrose metabolism
KacstDP.gene.S000494.5	KacstDP.mRNA.S000494.5	phosphofructokinase	Starch and sucrose metabolism
KacstDP.gene.S000015.136	KacstDP.mRNA.S000015.136	phosphofructokinase	Starch and sucrose metabolism
KacstDP.gene.S000159.19	KacstDP.mRNA.S000159.19	Fructokinase	Starch and sucrose metabolism
KacstDP.gene.S000047.19	KacstDP.mRNA.S000047.19	startch synthase SS	Starch and sucrose metabolism
KacstDP.gene.S000137.28	KacstDP.mRNA.S000137.28	startch synthase SS	Starch and sucrose metabolism
KacstDP.gene.S000021.77	KacstDP.mRNA.S000021.77	startch synthase SS	Starch and sucrose metabolism
KacstDP.gene.S000003.183	KacstDP.mRNA.S000003.183	startch synthase SS	Starch and sucrose metabolism
KacstDP.gene.S000228.17	KacstDP.mRNA.S000228.17	startch synthase SS	Starch and sucrose metabolism
KacstDP.gene.S000814.6	KacstDP.mRNA.S000814.6	startch synthase SS	Starch and sucrose metabolism
KacstDP.gene.S000008.68	KacstDP.mRNA.S000008.68	startch synthase SS	Starch and sucrose metabolism
KacstDP.gene.S000023.47	KacstDP.mRNA.S000023.47	Phosphoglucomutase	Starch and sucrose metabolism
KacstDP.gene.S000010.44	KacstDP.mRNA.S000010.44	Phosphoglucomutase	Starch and sucrose metabolism
KacstDP.gene.S000003.101	KacstDP.mRNA.S000003.101	Phosphoglucomutase	Starch and sucrose metabolism
KacstDP.gene.S027867.1	KacstDP.mRNA.S027867.1	Phosphoglucomutase	Starch and sucrose metabolism
KacstDP.gene.S000029.127	KacstDP.mRNA.S000029.127	Phosphoglucomutase	Starch and sucrose metabolism
KacstDP.gene.S000073.47	KacstDP.mRNA.S000073.47	Phosphoglucomutase	Starch and sucrose metabolism
KacstDP.gene.S001139.2	KacstDP.mRNA.S001139.2	Phosphoglycerate kinase	Starch and sucrose metabolism
KacstDP.gene.S000315.20	KacstDP.mRNA.S000315.20	Phosphoglycerate kinase	Starch and sucrose metabolism
KacstDP.gene.S000034.109	KacstDP.mRNA.S000034.109	Phosphoglycerate kinase	Starch and sucrose metabolism
KacstDP.gene.S001139.1	KacstDP.mRNA.S001139.1	Phosphoglycerate kinase	Starch and sucrose metabolism
KacstDP.gene.S000315.19	KacstDP.mRNA.S000315.19	Phosphoglycerate kinase	Starch and sucrose metabolism
KacstDP.gene.S000456.1	KacstDP.mRNA.S000456.1	Phosphoglycerate kinase	Starch and sucrose metabolism

KacstDP.gene.S000175.35	KacstDP.mRNA.S000175.35	ADP-glucose phosphorylase	Starch and sucrose metabolism
KacstDP.gene.S000665.8	KacstDP.mRNA.S000665.8	ADP-glucose phosphorylase	Starch and sucrose metabolism
KacstDP.gene.S000040.34	KacstDP.mRNA.S000040.34	ADP-glucose phosphorylase	Starch and sucrose metabolism
KacstDP.gene.S000103.40	KacstDP.mRNA.S000103.40	ADP-glucose phosphorylase	Starch and sucrose metabolism
KacstDP.gene.S000020.121	KacstDP.mRNA.S000020.121	ADP-glucose phosphorylase	Starch and sucrose metabolism
KacstDP.gene.S000187.20	KacstDP.mRNA.S000187.20	ADP-glucose phosphorylase	Starch and sucrose metabolism
KacstDP.gene.S000047.78	KacstDP.mRNA.S000047.78	ADP-glucose phosphorylase	Starch and sucrose metabolism
KacstDP.gene.S000551.2	KacstDP.mRNA.S000551.2	Fructose-1,6-bisphophate	Starch and sucrose metabolism
KacstDP.gene.S000031.61	KacstDP.mRNA.S000031.61	Fructose-1,6-bisphophate	Starch and sucrose metabolism
KacstDP.gene.S000777.12	KacstDP.mRNA.S000777.12	Fructose-1,6-bisphophate	Starch and sucrose metabolism
KacstDP.gene.S000314.6	KacstDP.mRNA.S000314.6	Fructose-1,6-bisphophate	Starch and sucrose metabolism
KacstDP.gene.S000094.36	KacstDP.mRNA.S000094.36	Fructose-1,6-bisphophate	Starch and sucrose metabolism
KacstDP.gene.S000033.50	KacstDP.mRNA.S000033.50	Fructose-1,6-bisphophate	Starch and sucrose metabolism
KacstDP.gene.S000014.108	KacstDP.mRNA.S000014.108	Fructose-bisphosphate aldolase	Starch and sucrose metabolism
KacstDP.gene.S000025.151	KacstDP.mRNA.S000025.151	Fructose-bisphosphate aldolase	Starch and sucrose metabolism
KacstDP.gene.S000020.125	KacstDP.mRNA.S000020.125	Fructose-bisphosphate aldolase	Starch and sucrose metabolism
KacstDP.gene.S000005.136	KacstDP.mRNA.S000005.136	Fructose-bisphosphate aldolase	Starch and sucrose metabolism
KacstDP.gene.S000798.9	KacstDP.mRNA.S000798.9	Fructose-bisphosphate aldolase	Starch and sucrose metabolism
KacstDP.gene.S000175.19	KacstDP.mRNA.S000175.19	Fructose-bisphosphate aldolase	Starch and sucrose metabolism
KacstDP.gene.S000074.42	KacstDP.mRNA.S000074.42	Fructose-bisphosphate aldolase	Starch and sucrose metabolism
KacstDP.gene.S000001.236	KacstDP.mRNA.S000001.236	Fructose-bisphosphate aldolase	Starch and sucrose metabolism
KacstDP.gene.S000504.12	KacstDP.mRNA.S000504.12	Triosephosphate isomerase	Starch and sucrose metabolism
KacstDP.gene.S000027.61	KacstDP.mRNA.S000027.61	Triosephosphate isomerase	Starch and sucrose metabolism
KacstDP.gene.S000166.45	KacstDP.mRNA.S000166.45	Triosephosphate isomerase	Starch and sucrose metabolism
KacstDP.gene.S000231.9	KacstDP.mRNA.S000231.9	Triosephosphate isomerase	Starch and sucrose metabolism
KacstDP.gene.S000316.14	KacstDP.mRNA.S000316.14	Malate deshydrogenase MDH	Starch and sucrose metabolism
KacstDP.gene.S000228.21	KacstDP.mRNA.S000228.21	Malate deshydrogenase MDH	Starch and sucrose metabolism

KacstDP.gene.S000002.122	KacstDP.mRNA.S000002.122	Malate deshydrogenase MDH	Starch and sucrose metabolism
KacstDP.gene.S000293.26	KacstDP.mRNA.S000293.26	Malate deshydrogenase MDH	Starch and sucrose metabolism
KacstDP.gene.S000073.82	KacstDP.mRNA.S000073.82	Malate deshydrogenase MDH	Starch and sucrose metabolism
KacstDP.gene.S000022.124	KacstDP.mRNA.S000022.124	glucose-6-phosphate isomerase	Starch and sucrose metabolism
KacstDP.gene.S000353.23	KacstDP.mRNA.S000353.23	Ribulose bisphosphate carboxylase small chain	Starch and sucrose metabolism
KacstDP.gene.S000141.43	KacstDP.mRNA.S000141.43	Ribulose bisphosphate carboxylase small chain	Starch and sucrose metabolism
KacstDP.gene.S000102.9	KacstDP.mRNA.S000102.9	Ribulose bisphosphate carboxylase small chain	Starch and sucrose metabolism
KacstDP.gene.S000251.26	KacstDP.mRNA.S000251.26	Ribulose bisphosphate carboxylase small chain	Starch and sucrose metabolism
KacstDP.gene.S000012.1	KacstDP.mRNA.S000012.1	Ribulose bisphosphate carboxylase small chain	Starch and sucrose metabolism
KacstDP.gene.S000004.28	KacstDP.mRNA.S000004.28	cellulase	Fruit ripening
KacstDP.gene.S000019.80	KacstDP.mRNA.S000019.80	cellulase	Fruit ripening
KacstDP.gene.S000049.24	KacstDP.mRNA.S000049.24	cellulase	Fruit ripening
KacstDP.gene.S000092.6	KacstDP.mRNA.S000092.6	cellulase	Fruit ripening
KacstDP.gene.S000092.7	KacstDP.mRNA.S000092.7	cellulase	Fruit ripening
KacstDP.gene.S000137.42	KacstDP.mRNA.S000137.42	cellulase	Fruit ripening
KacstDP.gene.S000187.21	KacstDP.mRNA.S000187.21	cellulase	Fruit ripening
KacstDP.gene.S000156.50	KacstDP.mRNA.S000156.50	cellulase	Fruit ripening
KacstDP.gene.S000175.33	KacstDP.mRNA.S000175.33	cellulase	Fruit ripening
KacstDP.gene.S000175.34	KacstDP.mRNA.S000175.34	cellulase	Fruit ripening
KacstDP.gene.S000180.13	KacstDP.mRNA.S000180.13	cellulase	Fruit ripening
KacstDP.gene.S000255.8	KacstDP.mRNA.S000255.8	cellulase	Fruit ripening
KacstDP.gene.S000255.9	KacstDP.mRNA.S000255.9	cellulase	Fruit ripening

KacstDP.gene.S000262.11	KacstDP.mRNA.S000262.11	cellulase	Fruit ripening
KacstDP.gene.S000310.3	KacstDP.mRNA.S000310.3	cellulase	Fruit ripening
KacstDP.gene.S000310.5	KacstDP.mRNA.S000310.5	cellulase	Fruit ripening
KacstDP.gene.S000441.1	KacstDP.mRNA.S000441.1	cellulase	Fruit ripening
KacstDP.gene.S000523.9	KacstDP.mRNA.S000523.9	cellulase	Fruit ripening
KacstDP.gene.S000795.1	KacstDP.mRNA.S000795.1	cellulase	Fruit ripening
KacstDP.gene.S000922.2	KacstDP.mRNA.S000922.2	cellulase	Fruit ripening
KacstDP.gene.S002007.1	KacstDP.mRNA.S002007.1	cellulase	Fruit ripening
KacstDP.gene.S000001.328	KacstDP.mRNA.S000001.328	alpha-amylase	Fruit ripening
KacstDP.gene.S000003.60	KacstDP.mRNA.S000003.60	alpha-amylase	Fruit ripening
KacstDP.gene.S000008.120	KacstDP.mRNA.S000008.120	alpha-amylase	Fruit ripening
KacstDP.gene.S000013.181	KacstDP.mRNA.S000013.181	alpha-amylase	Fruit ripening
KacstDP.gene.S000025.4	KacstDP.mRNA.S000025.4	alpha-amylase	Fruit ripening
KacstDP.gene.S000074.21	KacstDP.mRNA.S000074.21	alpha-amylase	Fruit ripening
KacstDP.gene.S000077.31	KacstDP.mRNA.S000077.31	alpha-amylase	Fruit ripening
KacstDP.gene.S000145.34	KacstDP.mRNA.S000145.34	alpha-amylase	Fruit ripening
KacstDP.gene.S000162.9	KacstDP.mRNA.S000162.9	alpha-amylase	Fruit ripening
KacstDP.gene.S000371.20	KacstDP.mRNA.S000371.20	alpha-amylase	Fruit ripening
KacstDP.gene.S000375.15	KacstDP.mRNA.S000375.15	alpha-amylase	Fruit ripening
KacstDP.gene.S000554.6	KacstDP.mRNA.S000554.6	alpha-amylase	Fruit ripening
KacstDP.gene.S000554.7	KacstDP.mRNA.S000554.7	alpha-amylase	Fruit ripening
KacstDP.gene.S000634.3	KacstDP.mRNA.S000634.3	alpha-amylase	Fruit ripening
KacstDP.gene.S000561.7	KacstDP.mRNA.S000561.7	carboxylesterase	Fruit ripening
KacstDP.gene.S000442.6	KacstDP.mRNA.S000442.6	carboxylesterase	Fruit ripening
KacstDP.gene.S000254.27	KacstDP.mRNA.S000254.27	carboxylesterase	Fruit ripening
KacstDP.gene.S000080.21	KacstDP.mRNA.S000080.21	carboxylesterase	Fruit ripening
KacstDP.gene.S001430.2	KacstDP.mRNA.S001430.2	carboxylesterase	Fruit ripening

KacstDP.gene.S000036.16	KacstDP.mRNA.S000036.16	carboxylesterase	Fruit ripening
KacstDP.gene.S000655.2	KacstDP.mRNA.S000655.2	carboxylesterase	Fruit ripening
KacstDP.gene.S000062.70	KacstDP.mRNA.S000062.70	carboxylesterase	Fruit ripening
KacstDP.gene.S000555.1	KacstDP.mRNA.S000555.1	Beta-galactosidase	Fruit ripening
KacstDP.gene.S000008.103	KacstDP.mRNA.S000008.103	Beta-galactosidase	Fruit ripening
KacstDP.gene.S000022.29	KacstDP.mRNA.S000022.29	Beta-galactosidase	Fruit ripening
KacstDP.gene.S000711.1	KacstDP.mRNA.S000711.1	Beta-galactosidase	Fruit ripening
KacstDP.gene.S000579.18	KacstDP.mRNA.S000579.18	Beta-galactosidase	Fruit ripening
KacstDP.gene.S000579.19	KacstDP.mRNA.S000579.19	Beta-galactosidase	Fruit ripening
KacstDP.gene.S000073.25	KacstDP.mRNA.S000073.25	Beta-galactosidase	Fruit ripening
KacstDP.gene.S000309.3	KacstDP.mRNA.S000309.3	Beta-galactosidase	Fruit ripening
KacstDP.gene.S000087.46	KacstDP.mRNA.S000087.46	Beta-galactosidase	Fruit ripening
KacstDP.gene.S000161.48	KacstDP.mRNA.S000161.48	Beta-galactosidase	Fruit ripening
KacstDP.gene.S000431.3	KacstDP.mRNA.S000431.3	Beta-galactosidase	Fruit ripening
KacstDP.gene.S000176.2	KacstDP.mRNA.S000176.2	Beta-galactosidase	Fruit ripening
KacstDP.gene.S000163.2	KacstDP.mRNA.S000163.2	Beta-galactosidase	Fruit ripening
KacstDP.gene.S000050.103	KacstDP.mRNA.S000050.103	Expansin	Fruit ripening
KacstDP.gene.S000354.5	KacstDP.mRNA.S000354.5	Expansin	Fruit ripening
KacstDP.gene.S000320.15	KacstDP.mRNA.S000320.15	Expansin	Fruit ripening
KacstDP.gene.S000228.6	KacstDP.mRNA.S000228.6	Expansin	Fruit ripening
KacstDP.gene.S000012.12	KacstDP.mRNA.S000012.12	Expansin	Fruit ripening
KacstDP.gene.S000019.54	KacstDP.mRNA.S000019.54	Expansin	Fruit ripening
KacstDP.gene.S000002.105	KacstDP.mRNA.S000002.105	Expansin	Fruit ripening
KacstDP.gene.S000257.17	KacstDP.mRNA.S000257.17	Expansin	Fruit ripening
KacstDP.gene.S000232.27	KacstDP.mRNA.S000232.27	Expansin	Fruit ripening
KacstDP.gene.S000006.59	KacstDP.mRNA.S000006.59	Expansin	Fruit ripening
KacstDP.gene.S000027.67	KacstDP.mRNA.S000027.67	Expansin	Fruit ripening

KacstDP.gene.S000010.179	KacstDP.mRNA.S000010.179	Expansin	Fruit ripening
KacstDP.gene.S000152.10	KacstDP.mRNA.S000152.10	Expansin	Fruit ripening
KacstDP.gene.S000394.10	KacstDP.mRNA.S000394.10	Expansin	Fruit ripening
KacstDP.gene.S000079.58	KacstDP.mRNA.S000079.58	Expansin	Fruit ripening
KacstDP.gene.S000770.3	KacstDP.mRNA.S000770.3	Expansin	Fruit ripening
KacstDP.gene.S000077.47	KacstDP.mRNA.S000077.47	Expansin	Fruit ripening
KacstDP.gene.S000527.n1549	KacstDP.mRNA.S000527.n1648	Expansin	Fruit ripening
KacstDP.gene.S000796.3	KacstDP.mRNA.S000796.3	Expansin	Fruit ripening
KacstDP.gene.S000035.15	KacstDP.mRNA.S000035.15	Expansin	Fruit ripening
KacstDP.gene.S000300.9	KacstDP.mRNA.S000300.9	Expansin	Fruit ripening
KacstDP.gene.S001366.3	KacstDP.mRNA.S001366.3	Expansin	Fruit ripening
KacstDP.gene.S000135.8	KacstDP.mRNA.S000135.8	Expansin	Fruit ripening
KacstDP.gene.S001145.1	KacstDP.mRNA.S001145.1	pectin methyltransferase (PMT)	Fruit ripening
KacstDP.gene.S000064.67	KacstDP.mRNA.S000064.67	pectin methyltransferase (PMT)	Fruit ripening
KacstDP.gene.S000230.23	KacstDP.mRNA.S000230.23	pectin methyltransferase (PMT)	Fruit ripening
KacstDP.gene.S000674.9	KacstDP.mRNA.S000674.9	O-acyl transferase	Fruit ripening
KacstDP.gene.S000151.29	KacstDP.mRNA.S000151.29	O-acyl transferase	Fruit ripening
KacstDP.gene.S000134.42	KacstDP.mRNA.S000134.42	1-aminocyclopropane-1-carboxylate synthase	Fruit ripening
KacstDP.gene.S000032.101	KacstDP.mRNA.S000032.101	1-aminocyclopropane-1-carboxylate synthase	Fruit ripening
KacstDP.gene.S000062.69	KacstDP.mRNA.S000062.69	1-aminocyclopropane-1-carboxylate synthase	Fruit ripening
KacstDP.gene.S000099.66	KacstDP.mRNA.S000099.66	1-aminocyclopropane-1-carboxylate synthase	Fruit ripening
KacstDP.gene.S000011.151	KacstDP.mRNA.S000011.151	1-aminocyclopropane-1-carboxylate synthase	Fruit ripening

KacstDP.gene.S000619.3	KacstDP.mRNA.S000619.3	pectin(pectate) lyase	Fruit ripening
KacstDP.gene.S000273.13	KacstDP.mRNA.S000273.13	pectin(pectate) lyase	Fruit ripening
KacstDP.gene.S000002.303	KacstDP.mRNA.S000002.303	pectin(pectate) lyase	Fruit ripening
KacstDP.gene.S000119.3	KacstDP.mRNA.S000119.3	pectin(pectate) lyase	Fruit ripening
KacstDP.gene.S000184.29	KacstDP.mRNA.S000184.29	pectin(pectate) lyase	Fruit ripening
KacstDP.gene.S000273.7	KacstDP.mRNA.S000273.7	pectin(pectate) lyase	Fruit ripening
KacstDP.gene.S000619.9	KacstDP.mRNA.S000619.9	pectin(pectate) lyase	Fruit ripening
KacstDP.gene.S000004.214	KacstDP.mRNA.S000004.214	pectin(pectate) lyase	Fruit ripening
KacstDP.gene.S001142.8	KacstDP.mRNA.S001142.8	pectin(pectate) lyase	Fruit ripening
KacstDP.gene.S003461.1	KacstDP.mRNA.S003461.1	pectin(pectate) lyase	Fruit ripening
KacstDP.gene.S000210.36	KacstDP.mRNA.S000210.36	pectin(pectate) lyase	Fruit ripening
KacstDP.gene.S018126.1	KacstDP.mRNA.S018126.1	plant invertase/pectin methylesterase	Fruit ripening
KacstDP.gene.S000116.6	KacstDP.mRNA.S000116.6	plant invertase/pectin methylesterase	Fruit ripening
KacstDP.gene.S000059.45	KacstDP.mRNA.S000059.45	plant invertase/pectin methylesterase	Fruit ripening
KacstDP.gene.S000464.19	KacstDP.mRNA.S000464.19	plant invertase/pectin methylesterase	Fruit ripening
KacstDP.gene.S000624.4	KacstDP.mRNA.S000624.4	plant invertase/pectin methylesterase	Fruit ripening
KacstDP.gene.S000913.7	KacstDP.mRNA.S000913.7	plant invertase/pectin methylesterase	Fruit ripening
KacstDP.gene.S000136.41	KacstDP.mRNA.S000136.41	plant invertase/pectin methylesterase	Fruit ripening
KacstDP.gene.S000074.69	KacstDP.mRNA.S000074.69	plant invertase/pectin methylesterase	Fruit ripening
KacstDP.gene.S003125.1	KacstDP.mRNA.S003125.1	plant invertase/pectin methylesterase	Fruit ripening
KacstDP.gene.S000135.26	KacstDP.mRNA.S000135.26	plant invertase/pectin methylesterase	Fruit ripening
KacstDP.gene.S000618.10	KacstDP.mRNA.S000618.10	plant invertase/pectin methylesterase	Fruit ripening
KacstDP.gene.S000183.28	KacstDP.mRNA.S000183.28	plant invertase/pectin methylesterase	Fruit ripening
KacstDP.gene.S000116.58	KacstDP.mRNA.S000116.58	plant invertase/pectin methylesterase	Fruit ripening
KacstDP.gene.S000116.5	KacstDP.mRNA.S000116.5	plant invertase/pectin methylesterase	Fruit ripening
KacstDP.gene.S000018.111	KacstDP.mRNA.S000018.111	plant invertase/pectin methylesterase	Fruit ripening
KacstDP.gene.S000002.155	KacstDP.mRNA.S000002.155	plant invertase/pectin methylesterase	Fruit ripening

KacstDP.gene.S000021.100	KacstDP.mRNA.S000021.100	plant invertase/pectin methylesterase	Fruit ripening
KacstDP.gene.S000001.192	KacstDP.mRNA.S000001.192	plant invertase/pectin methylesterase	Fruit ripening
KacstDP.gene.S000104.54	KacstDP.mRNA.S000104.54	plant invertase/pectin methylesterase	Fruit ripening
KacstDP.gene.S000028.4	KacstDP.mRNA.S000028.4	plant invertase/pectin methylesterase	Fruit ripening
KacstDP.gene.S000059.44	KacstDP.mRNA.S000059.44	plant invertase/pectin methylesterase	Fruit ripening
KacstDP.gene.S000116.4	KacstDP.mRNA.S000116.4	plant invertase/pectin methylesterase	Fruit ripening
KacstDP.gene.S000160.12	KacstDP.mRNA.S000160.12	plant invertase/pectin methylesterase	Fruit ripening
KacstDP.gene.S000036.118	KacstDP.mRNA.S000036.118	plant invertase/pectin methylesterase	Fruit ripening
KacstDP.gene.S000050.81	KacstDP.mRNA.S000050.81	plant invertase/pectin methylesterase	Fruit ripening
KacstDP.gene.S000392.14	KacstDP.mRNA.S000392.14	plant invertase/pectin methylesterase	Fruit ripening
KacstDP.gene.S000392.13	KacstDP.mRNA.S000392.13	plant invertase/pectin methylesterase	Fruit ripening
KacstDP.gene.S000027.99	KacstDP.mRNA.S000027.99	plant invertase/pectin methylesterase	Fruit ripening
KacstDP.gene.S000013.11	KacstDP.mRNA.S000013.11	polygalacturonase activity	Fruit ripening
KacstDP.gene.S000636.12	KacstDP.mRNA.S000636.12	polygalacturonase activity	Fruit ripening
KacstDP.gene.S000187.43	KacstDP.mRNA.S000187.43	polygalacturonase activity	Fruit ripening
KacstDP.gene.S000026.43	KacstDP.mRNA.S000026.43	polygalacturonase activity	Fruit ripening
KacstDP.gene.S000645.3	KacstDP.mRNA.S000645.3	polygalacturonase activity	Fruit ripening
KacstDP.gene.S000191.2	KacstDP.mRNA.S000191.2	polygalacturonase activity	Fruit ripening
KacstDP.gene.S001089.1	KacstDP.mRNA.S001089.1	polygalacturonase activity	Fruit ripening
KacstDP.gene.S000561.12	KacstDP.mRNA.S000561.12	polygalacturonase activity	Fruit ripening
KacstDP.gene.S000013.108	KacstDP.mRNA.S000013.108	polygalacturonase activity	Fruit ripening
KacstDP.gene.S000580.16	KacstDP.mRNA.S000580.16	polygalacturonase activity	Fruit ripening
KacstDP.gene.S000047.59	KacstDP.mRNA.S000047.59	polygalacturonase activity	Fruit ripening
KacstDP.gene.S000758.5	KacstDP.mRNA.S000758.5	polygalacturonase activity	Fruit ripening
KacstDP.gene.S000047.58	KacstDP.mRNA.S000047.58	polygalacturonase activity	Fruit ripening
KacstDP.gene.S000047.57	KacstDP.mRNA.S000047.57	polygalacturonase activity	Fruit ripening
KacstDP.gene.S000006.142	KacstDP.mRNA.S000006.142	polygalacturonase activity	Fruit ripening

KacstDP.gene.S000358.12	KacstDP.mRNA.S000358.12	polygalacturonase activity	Fruit ripening
KacstDP.gene.S000155.34	KacstDP.mRNA.S000155.34	polygalacturonase activity	Fruit ripening
KacstDP.gene.S000362.5	KacstDP.mRNA.S000362.5	polygalacturonase activity	Fruit ripening
KacstDP.gene.S000084.11	KacstDP.mRNA.S000084.11	polygalacturonase activity	Fruit ripening
KacstDP.gene.S000476.9	KacstDP.mRNA.S000476.9	polygalacturonase activity	Fruit ripening
KacstDP.gene.S000115.37	KacstDP.mRNA.S000115.37	polygalacturonase activity	Fruit ripening
KacstDP.gene.S000104.22	KacstDP.mRNA.S000104.22	polygalacturonase activity	Fruit ripening
KacstDP.gene.S000379.4	KacstDP.mRNA.S000379.4	polygalacturonase activity	Fruit ripening
KacstDP.gene.S000047.9	KacstDP.mRNA.S000047.9	polygalacturonase activity	Fruit ripening
KacstDP.gene.S000300.12	KacstDP.mRNA.S000300.12	polygalacturonase activity	Fruit ripening
KacstDP.gene.S000437.5	KacstDP.mRNA.S000437.5	polygalacturonase activity	Fruit ripening
KacstDP.gene.S000518.16	KacstDP.mRNA.S000518.16	polygalacturonase activity	Fruit ripening
KacstDP.gene.S000001.93	KacstDP.mRNA.S000001.93.1	polygalacturonase activity	Fruit ripening
KacstDP.gene.S000001.93	KacstDP.mRNA.S000001.93	polygalacturonase activity	Fruit ripening
KacstDP.gene.S000521.10	KacstDP.mRNA.S000521.10	polygalacturonase activity	Fruit ripening
KacstDP.gene.S000115.62	KacstDP.mRNA.S000115.62	polygalacturonase activity	Fruit ripening
KacstDP.gene.S000241.25	KacstDP.mRNA.S000241.25	xyloglucan:xyloglucosyl transferase	Fruit ripening
KacstDP.gene.S000032.n418	KacstDP.mRNA.S000032.n451	xyloglucan:xyloglucosyl transferase	Fruit ripening
KacstDP.gene.S000202.21	KacstDP.mRNA.S000202.21	xyloglucan:xyloglucosyl transferase	Fruit ripening
KacstDP.gene.S000334.23	KacstDP.mRNA.S000334.23	xyloglucan:xyloglucosyl transferase	Fruit ripening
KacstDP.gene.S000227.24	KacstDP.mRNA.S000227.24	xyloglucan:xyloglucosyl transferase	Fruit ripening
KacstDP.gene.S000177.17	KacstDP.mRNA.S000177.17	xyloglucan:xyloglucosyl transferase	Fruit ripening
KacstDP.gene.S000021.133	KacstDP.mRNA.S000021.133	xyloglucan:xyloglucosyl transferase	Fruit ripening
KacstDP.gene.S000063.89	KacstDP.mRNA.S000063.89	xyloglucan:xyloglucosyl transferase	Fruit ripening
KacstDP.gene.S000164.13	KacstDP.mRNA.S000164.13	NA	TN
KacstDP.gene.S000178.12	KacstDP.mRNA.S000178.12	NA	TN
KacstDP.gene.S000004.173	KacstDP.mRNA.S000004.173	NA	RLP

KacstDP.gene.S000312.19	KacstDP.mRNA.S000312.19	NA	RLP
KacstDP.gene.S000521.18	KacstDP.mRNA.S000521.18	NA	RLP
KacstDP.gene.S000167.23	KacstDP.mRNA.S000167.23	NA	RLP
KacstDP.gene.S015303.1	KacstDP.mRNA.S015303.1	NA	L
KacstDP.gene.S000096.44	KacstDP.mRNA.S000096.44	NA	L
KacstDP.gene.S000017.121	KacstDP.mRNA.S000017.121	NA	RLP
KacstDP.gene.S000153.40	KacstDP.mRNA.S000153.40	NA	RLP
KacstDP.gene.S035254.1	KacstDP.mRNA.S035254.1	NA	L
KacstDP.gene.S000033.36	KacstDP.mRNA.S000033.36	NA	RLP
KacstDP.gene.S002109.2	KacstDP.mRNA.S002109.2	NA	L
KacstDP.gene.S000060.20	KacstDP.mRNA.S000060.20	NA	RLP
KacstDP.gene.S000058.19	KacstDP.mRNA.S000058.19	NA	L
KacstDP.gene.S000356.23	KacstDP.mRNA.S000356.23	NA	RLP
KacstDP.gene.S000101.44	KacstDP.mRNA.S000101.44	NA	RLP
KacstDP.gene.S000015.100	KacstDP.mRNA.S000015.100	NA	RLP
KacstDP.gene.S000040.8	KacstDP.mRNA.S000040.8	NA	L
KacstDP.gene.S000015.149	KacstDP.mRNA.S000015.149	NA	RLP
KacstDP.gene.S001181.2	KacstDP.mRNA.S001181.2.2	NA	RLP
KacstDP.gene.S001181.2	KacstDP.mRNA.S001181.2.3	NA	RLP
KacstDP.gene.S001181.2	KacstDP.mRNA.S001181.2	NA	RLP
KacstDP.gene.S001181.2	KacstDP.mRNA.S001181.2.1	NA	RLP
KacstDP.gene.S025465.1	KacstDP.mRNA.S025465.1	NA	L
KacstDP.gene.S020771.1	KacstDP.mRNA.S020771.1	NA	RLP
KacstDP.gene.S000011.42	KacstDP.mRNA.S000011.42	NA	RLP
KacstDP.gene.S000172.19	KacstDP.mRNA.S000172.19	NA	RLP
KacstDP.gene.S000217.4	KacstDP.mRNA.S000217.4	NA	RLP
KacstDP.gene.S000005.83	KacstDP.mRNA.S000005.83	NA	L

KacstDP.gene.S000001.n29	KacstDP.mRNA.S000001.n32	NA	RLP
KacstDP.gene.S000017.69	KacstDP.mRNA.S000017.69	NA	RLP
KacstDP.gene.S000195.17	KacstDP.mRNA.S000195.17	NA	L
KacstDP.gene.S000131.26	KacstDP.mRNA.S000131.26	NA	RLP
KacstDP.gene.S000089.54	KacstDP.mRNA.S000089.54	NA	RLP
KacstDP.gene.S000089.18	KacstDP.mRNA.S000089.18	NA	L
KacstDP.gene.S000002.277	KacstDP.mRNA.S000002.277	NA	RLP
KacstDP.gene.S000850.2	KacstDP.mRNA.S000850.2	NA	RLP
KacstDP.gene.S000001.335	KacstDP.mRNA.S000001.335	NA	RLP
KacstDP.gene.S007169.1	KacstDP.mRNA.S007169.1	NA	RLP
KacstDP.gene.S008113.1	KacstDP.mRNA.S008113.1	NA	RLP
KacstDP.gene.S002927.1	KacstDP.mRNA.S002927.1	NA	RLP
KacstDP.gene.S001121.2	KacstDP.mRNA.S001121.2	NA	RLP
KacstDP.gene.S000001.18	KacstDP.mRNA.S000001.18	NA	RLP
KacstDP.gene.S000262.6	KacstDP.mRNA.S000262.6	NA	RLP
KacstDP.gene.S000199.4	KacstDP.mRNA.S000199.4	NA	RLP
KacstDP.gene.S000059.58	KacstDP.mRNA.S000059.58	NA	L
KacstDP.gene.S000190.14	KacstDP.mRNA.S000190.14	NA	RLP
KacstDP.gene.S000349.6	KacstDP.mRNA.S000349.6	NA	RLP
KacstDP.gene.S000073.71	KacstDP.mRNA.S000073.71	NA	RLP
KacstDP.gene.S000134.17	KacstDP.mRNA.S000134.17	NA	RLP
KacstDP.gene.S000129.20	KacstDP.mRNA.S000129.20	NA	RLP
KacstDP.gene.S000006.63	KacstDP.mRNA.S000006.63	NA	RLP
KacstDP.gene.S000062.29	KacstDP.mRNA.S000062.29	NA	RLP
KacstDP.gene.S000855.3	KacstDP.mRNA.S000855.3	NA	RLP
KacstDP.gene.S000128.55	KacstDP.mRNA.S000128.55	NA	RLP
KacstDP.gene.S019915.1	KacstDP.mRNA.S019915.1	NA	RLP

KacstDP.gene.S000007.31	KacstDP.mRNA.S000007.31	NA	RLP
KacstDP.gene.S000096.13	KacstDP.mRNA.S000096.13	NA	RLP
KacstDP.gene.S000063.56	KacstDP.mRNA.S000063.56	NA	RLP
KacstDP.gene.S000040.3	KacstDP.mRNA.S000040.3	NA	RLP
KacstDP.gene.S000106.53	KacstDP.mRNA.S000106.53	NA	L
KacstDP.gene.S043475.1	KacstDP.mRNA.S043475.1	NA	RLP
KacstDP.gene.S000199.11	KacstDP.mRNA.S000199.11	NA	RLP
KacstDP.gene.S000457.21	KacstDP.mRNA.S000457.21	NA	RLP
KacstDP.gene.S000199.8	KacstDP.mRNA.S000199.8	NA	RLP
KacstDP.gene.S000152.n1008	KacstDP.mRNA.S000152.n1076	NA	RLP
KacstDP.gene.S000286.12	KacstDP.mRNA.S000286.12	NA	RLP
KacstDP.gene.S000124.20	KacstDP.mRNA.S000124.20	NA	RLP
KacstDP.gene.S000222.18	KacstDP.mRNA.S000222.18	NA	RLP
KacstDP.gene.S000423.9	KacstDP.mRNA.S000423.9	NA	RLP
KacstDP.gene.S000035.103	KacstDP.mRNA.S000035.103	NA	L
KacstDP.gene.S000025.60	KacstDP.mRNA.S000025.60	NA	L
KacstDP.gene.S000062.63	KacstDP.mRNA.S000062.63	NA	RLP
KacstDP.gene.S000032.107	KacstDP.mRNA.S000032.107	NA	RLP
KacstDP.gene.S000212.19	KacstDP.mRNA.S000212.19	NA	RLP
KacstDP.gene.S000255.34	KacstDP.mRNA.S000255.34	NA	RLP
KacstDP.gene.S000359.6	KacstDP.mRNA.S000359.6	NA	RLP
KacstDP.gene.S014157.1	KacstDP.mRNA.S014157.1	NA	RLP
KacstDP.gene.S000630.8	KacstDP.mRNA.S000630.8	NA	RLP
KacstDP.gene.S000456.2	KacstDP.mRNA.S000456.2	NA	RLP
KacstDP.gene.S000108.21	KacstDP.mRNA.S000108.21	NA	RLP
KacstDP.gene.S023072.1	KacstDP.mRNA.S023072.1	NA	L
KacstDP.gene.S001082.2	KacstDP.mRNA.S001082.2	NA	L

KacstDP.gene.S000199.7	KacstDP.mRNA.S000199.7	NA	RLP
KacstDP.gene.S000053.106	KacstDP.mRNA.S000053.106	NA	RLP
KacstDP.gene.S000053.105	KacstDP.mRNA.S000053.105	NA	RLP
KacstDP.gene.S000443.16	KacstDP.mRNA.S000443.16	NA	RLP
KacstDP.gene.S000199.9	KacstDP.mRNA.S000199.9	NA	RLP
KacstDP.gene.S000007.209	KacstDP.mRNA.S000007.209	NA	RLK
KacstDP.gene.S000053.116	KacstDP.mRNA.S000053.116	NA	RLK
KacstDP.gene.S000055.79	KacstDP.mRNA.S000055.79	NA	RLK
KacstDP.gene.S000133.54	KacstDP.mRNA.S000133.54	NA	RLK
KacstDP.gene.S000346.20	KacstDP.mRNA.S000346.20	NA	RLK
KacstDP.gene.S000224.2	KacstDP.mRNA.S000224.2	NA	RLK
KacstDP.gene.S000765.3	KacstDP.mRNA.S000765.3	NA	RLK
KacstDP.gene.S000270.28	KacstDP.mRNA.S000270.28	NA	RLK
KacstDP.gene.S000002.33	KacstDP.mRNA.S000002.33	NA	RLK
KacstDP.gene.S000101.28	KacstDP.mRNA.S000101.28	NA	RLK
KacstDP.gene.S000071.60	KacstDP.mRNA.S000071.60	NA	RLK
KacstDP.gene.S000261.19	KacstDP.mRNA.S000261.19	NA	RLK
KacstDP.gene.S000012.120	KacstDP.mRNA.S000012.120	NA	RLK
KacstDP.gene.S000202.43	KacstDP.mRNA.S000202.43	NA	RLK
KacstDP.gene.S000145.52	KacstDP.mRNA.S000145.52	NA	RLK
KacstDP.gene.S000015.24	KacstDP.mRNA.S000015.24	NA	RLK
KacstDP.gene.S000001.120	KacstDP.mRNA.S000001.120	NA	RLK
KacstDP.gene.S000107.7	KacstDP.mRNA.S000107.7	NA	RLK
KacstDP.gene.S000457.22	KacstDP.mRNA.S000457.22	NA	RLK
KacstDP.gene.S000162.2	KacstDP.mRNA.S000162.2	NA	RLK
KacstDP.gene.S000001.349	KacstDP.mRNA.S000001.349.1	NA	C
KacstDP.gene.S000001.349	KacstDP.mRNA.S000001.349.2	NA	C

KacstDP.gene.S000100.7	KacstDP.mRNA.S000100.7.1	NA	RLK
KacstDP.gene.S000100.7	KacstDP.mRNA.S000100.7	NA	RLK
KacstDP.gene.S000084.64	KacstDP.mRNA.S000084.64	NA	RLK
KacstDP.gene.S000084.64	KacstDP.mRNA.S000084.64.1	NA	RLK
KacstDP.gene.S000193.37	KacstDP.mRNA.S000193.37	NA	C
KacstDP.gene.S000056.86	KacstDP.mRNA.S000056.86	NA	RLK
KacstDP.gene.S000020.17	KacstDP.mRNA.S000020.17	NA	RLK
KacstDP.gene.S000389.18	KacstDP.mRNA.S000389.18	NA	RLK
KacstDP.gene.S000944.4	KacstDP.mRNA.S000944.4	NA	RLK
KacstDP.gene.S000009.188	KacstDP.mRNA.S000009.188	NA	RLK
KacstDP.gene.S003085.1	KacstDP.mRNA.S003085.1	NA	RLK
KacstDP.gene.S003621.1	KacstDP.mRNA.S003621.1	NA	RLK
KacstDP.gene.S000115.39	KacstDP.mRNA.S000115.39	NA	RLK
KacstDP.gene.S000173.n1047	KacstDP.mRNA.S000173.n1116	NA	RLK
KacstDP.gene.S000041.48	KacstDP.mRNA.S000041.48	NA	RLK
KacstDP.gene.S000010.43	KacstDP.mRNA.S000010.43	NA	RLK
KacstDP.gene.S000519.13	KacstDP.mRNA.S000519.13	NA	RLK
KacstDP.gene.S000183.32	KacstDP.mRNA.S000183.32	NA	RLK
KacstDP.gene.S001042.1	KacstDP.mRNA.S001042.1	NA	RLK
KacstDP.gene.S000008.169	KacstDP.mRNA.S000008.169	NA	RLK
KacstDP.gene.S000693.3	KacstDP.mRNA.S000693.3	NA	RLK
KacstDP.gene.S000259.4	KacstDP.mRNA.S000259.4	NA	RLK
KacstDP.gene.S000273.26	KacstDP.mRNA.S000273.26	NA	RLK
KacstDP.gene.S000394.5	KacstDP.mRNA.S000394.5	NA	RLK
KacstDP.gene.S000002.82	KacstDP.mRNA.S000002.82	NA	RLK
KacstDP.gene.S000243.13	KacstDP.mRNA.S000243.13	NA	RLK
KacstDP.gene.S000006.19	KacstDP.mRNA.S000006.19	NA	RLK

KacstDP.gene.S000041.13	KacstDP.mRNA.S000041.13	NA	RLK
KacstDP.gene.S000469.2	KacstDP.mRNA.S000469.2	NA	RLK
KacstDP.gene.S000046.90	KacstDP.mRNA.S000046.90	NA	RLK
KacstDP.gene.S000060.21	KacstDP.mRNA.S000060.21	NA	RLK
KacstDP.gene.S000032.71	KacstDP.mRNA.S000032.71	NA	RLK
KacstDP.gene.S000047.54	KacstDP.mRNA.S000047.54	NA	RLK
KacstDP.gene.S000624.6	KacstDP.mRNA.S000624.6	NA	RLK
KacstDP.gene.S000019.94	KacstDP.mRNA.S000019.94	NA	RLK
KacstDP.gene.S000029.63	KacstDP.mRNA.S000029.63	NA	RLK
KacstDP.gene.S000237.10	KacstDP.mRNA.S000237.10	NA	RLK
KacstDP.gene.S000819.4	KacstDP.mRNA.S000819.4	NA	RLK
KacstDP.gene.S000939.1	KacstDP.mRNA.S000939.1	NA	RLK
KacstDP.gene.S000249.35	KacstDP.mRNA.S000249.35	NA	RLK
KacstDP.gene.S000181.16	KacstDP.mRNA.S000181.16	NA	RLK
KacstDP.gene.S007049.1	KacstDP.mRNA.S007049.1	NA	RLK
KacstDP.gene.S000006.141	KacstDP.mRNA.S000006.141	NA	RLK
KacstDP.gene.S000006.n122	KacstDP.mRNA.S000006.n136	NA	RLK
KacstDP.gene.S000044.30	KacstDP.mRNA.S000044.30	NA	RLK
KacstDP.gene.S000237.17	KacstDP.mRNA.S000237.17	NA	RLK
KacstDP.gene.S000429.18	KacstDP.mRNA.S000429.18	NA	RLK
KacstDP.gene.S000044.41	KacstDP.mRNA.S000044.41	NA	RLK
KacstDP.gene.S000023.113	KacstDP.mRNA.S000023.113	NA	RLK
KacstDP.gene.S000089.46	KacstDP.mRNA.S000089.46	NA	RLK
KacstDP.gene.S000006.187	KacstDP.mRNA.S000006.187	NA	RLK
KacstDP.gene.S000730.2	KacstDP.mRNA.S000730.2	NA	RLK
KacstDP.gene.S000223.1	KacstDP.mRNA.S000223.1	NA	RLK
KacstDP.gene.S000006.188	KacstDP.mRNA.S000006.188	NA	RLK

KacstDP.gene.S000024.6	KacstDP.mRNA.S000024.6	NA	RLK
KacstDP.gene.S000042.30	KacstDP.mRNA.S000042.30	NA	RLK
KacstDP.gene.S000432.18	KacstDP.mRNA.S000432.18	NA	RLK
KacstDP.gene.S002703.2	KacstDP.mRNA.S002703.2	NA	RLK
KacstDP.gene.S000457.3	KacstDP.mRNA.S000457.3	NA	RLK
KacstDP.gene.S000423.2	KacstDP.mRNA.S000423.2	NA	RLK
KacstDP.gene.S000042.29	KacstDP.mRNA.S000042.29	NA	RLK
KacstDP.gene.S000110.30	KacstDP.mRNA.S000110.30	NA	RLK
KacstDP.gene.S000278.30	KacstDP.mRNA.S000278.30	NA	RLK
KacstDP.gene.S001413.3	KacstDP.mRNA.S001413.3	NA	RLK
KacstDP.gene.S000343.2	KacstDP.mRNA.S000343.2	NA	RLK
KacstDP.gene.S000019.124	KacstDP.mRNA.S000019.124	NA	RLK
KacstDP.gene.S000005.213	KacstDP.mRNA.S000005.213	NA	RLK
KacstDP.gene.S000850.3	KacstDP.mRNA.S000850.3	NA	RLK
KacstDP.gene.S000457.16	KacstDP.mRNA.S000457.16	NA	RLK
KacstDP.gene.S000034.41	KacstDP.mRNA.S000034.41	NA	RLK
KacstDP.gene.S005017.1	KacstDP.mRNA.S005017.1	NA	RLK
KacstDP.gene.S000236.13	KacstDP.mRNA.S000236.13	NA	RLK
KacstDP.gene.S000088.62	KacstDP.mRNA.S000088.62	NA	RLK
KacstDP.gene.S000088.63	KacstDP.mRNA.S000088.63	NA	RLK
KacstDP.gene.S000080.30	KacstDP.mRNA.S000080.30	NA	RLK
KacstDP.gene.S004071.1	KacstDP.mRNA.S004071.1	NA	RLK
KacstDP.gene.S000020.101	KacstDP.mRNA.S000020.101	NA	RLK
KacstDP.gene.S000058.78	KacstDP.mRNA.S000058.78	NA	RLK
KacstDP.gene.S000850.1	KacstDP.mRNA.S000850.1	NA	RLK
KacstDP.gene.S000007.130	KacstDP.mRNA.S000007.130	NA	RLK
KacstDP.gene.S000443.17	KacstDP.mRNA.S000443.17	NA	RLK

KacstDP.gene.S000275.1	KacstDP.mRNA.S000275.1	NA	RLK
KacstDP.gene.S009692.1	KacstDP.mRNA.S009692.1	NA	RLK
KacstDP.gene.S000733.6	KacstDP.mRNA.S000733.6.3	NA	RLK
KacstDP.gene.S000457.2	KacstDP.mRNA.S000457.2	NA	RLK
KacstDP.gene.S000317.3	KacstDP.mRNA.S000317.3	NA	RLK
KacstDP.gene.S000112.29	KacstDP.mRNA.S000112.29	NA	RLK
KacstDP.gene.S007590.1	KacstDP.mRNA.S007590.1	NA	RLK
KacstDP.gene.S000261.8	KacstDP.mRNA.S000261.8	NA	RLK
KacstDP.gene.S000617.8	KacstDP.mRNA.S000617.8	NA	RLK
KacstDP.gene.S000060.5	KacstDP.mRNA.S000060.5	NA	RLK
KacstDP.gene.S000123.62	KacstDP.mRNA.S000123.62	NA	RLK
KacstDP.gene.S000032.82	KacstDP.mRNA.S000032.82	NA	RLK
KacstDP.gene.S000086.73	KacstDP.mRNA.S000086.73.1	NA	RLK
KacstDP.gene.S000086.73	KacstDP.mRNA.S000086.73	NA	RLK
KacstDP.gene.S000037.41	KacstDP.mRNA.S000037.41	NA	RLK
KacstDP.gene.S000037.41	KacstDP.mRNA.S000037.41.1	NA	RLK
KacstDP.gene.S000021.24	KacstDP.mRNA.S000021.24	NA	RLK
KacstDP.gene.S000001.270	KacstDP.mRNA.S000001.270	NA	RLK
KacstDP.gene.S000953.2	KacstDP.mRNA.S000953.2	NA	RLK
KacstDP.gene.S000683.8	KacstDP.mRNA.S000683.8	NA	RLK
KacstDP.gene.S000044.42	KacstDP.mRNA.S000044.42	NA	RLK
KacstDP.gene.S000019.126	KacstDP.mRNA.S000019.126	NA	RLK
KacstDP.gene.S000061.67	KacstDP.mRNA.S000061.67	NA	RLK
KacstDP.gene.S001149.2	KacstDP.mRNA.S001149.2	NA	RLK
KacstDP.gene.S000008.138	KacstDP.mRNA.S000008.138	NA	RLK
KacstDP.gene.S000002.288	KacstDP.mRNA.S000002.288	NA	RLK
KacstDP.gene.S000454.4	KacstDP.mRNA.S000454.4	NA	RLK

KacstDP.gene.S000280.21	KacstDP.mRNA.S000280.21	NA	RLK
KacstDP.gene.S000451.12	KacstDP.mRNA.S000451.12	NA	RLK
KacstDP.gene.S017578.1	KacstDP.mRNA.S017578.1	NA	RLK
KacstDP.gene.S000116.10	KacstDP.mRNA.S000116.10	NA	RLK
KacstDP.gene.S000103.11	KacstDP.mRNA.S000103.11	NA	RLK
KacstDP.gene.S000067.63	KacstDP.mRNA.S000067.63	NA	RLK
KacstDP.gene.S000219.11	KacstDP.mRNA.S000219.11	NA	RLK
KacstDP.gene.S000015.142	KacstDP.mRNA.S000015.142	NA	RLK
KacstDP.gene.S000630.1	KacstDP.mRNA.S000630.1	NA	RLK
KacstDP.gene.S000045.61	KacstDP.mRNA.S000045.61	NA	RLK
KacstDP.gene.S000005.240	KacstDP.mRNA.S000005.240	NA	RLK
KacstDP.gene.S000001.12	KacstDP.mRNA.S000001.12	NA	RLK
KacstDP.gene.S000054.65	KacstDP.mRNA.S000054.65	NA	RLK
KacstDP.gene.S000113.54	KacstDP.mRNA.S000113.54	NA	RLK
KacstDP.gene.S000817.7	KacstDP.mRNA.S000817.7	NA	RLK
KacstDP.gene.S000518.4	KacstDP.mRNA.S000518.4	NA	RLK
KacstDP.gene.S000518.2	KacstDP.mRNA.S000518.2	NA	RLK
KacstDP.gene.S000126.28	KacstDP.mRNA.S000126.28.1	NA	RLK
KacstDP.gene.S000126.28	KacstDP.mRNA.S000126.28	NA	RLK
KacstDP.gene.S000624.8	KacstDP.mRNA.S000624.8	NA	RLK
KacstDP.gene.S000012.45	KacstDP.mRNA.S000012.45	NA	RLK
KacstDP.gene.S000100.59	KacstDP.mRNA.S000100.59	NA	RLK
KacstDP.gene.S000111.6	KacstDP.mRNA.S000111.6	NA	RLK
KacstDP.gene.S000707.8	KacstDP.mRNA.S000707.8	NA	NL
KacstDP.gene.S000054.8	KacstDP.mRNA.S000054.8	NA	NL
KacstDP.gene.S000707.5	KacstDP.mRNA.S000707.5	NA	N
KacstDP.gene.S000940.3	KacstDP.mRNA.S000940.3	NA	N

KacstDP.gene.S000083.13	KacstDP.mRNA.S000083.13	NA	N
KacstDP.gene.S001281.3	KacstDP.mRNA.S001281.3	NA	N
KacstDP.gene.S000121.18	KacstDP.mRNA.S000121.18	NA	CN
KacstDP.gene.S022075.1	KacstDP.mRNA.S022075.1	NA	N
KacstDP.gene.S024301.1	KacstDP.mRNA.S024301.1	NA	N
KacstDP.gene.S000474.11	KacstDP.mRNA.S000474.11	NA	N
KacstDP.gene.S048247.1	KacstDP.mRNA.S048247.1	NA	N
KacstDP.gene.S061251.1	KacstDP.mRNA.S061251.1	NA	N
KacstDP.gene.S053495.1	KacstDP.mRNA.S053495.1	NA	N
KacstDP.gene.S000290.23	KacstDP.mRNA.S000290.23	NA	N
KacstDP.gene.S000025.165	KacstDP.mRNA.S000025.165	NA	N
KacstDP.gene.S038271.1	KacstDP.mRNA.S038271.1	NA	N
KacstDP.gene.S000268.27	KacstDP.mRNA.S000268.27	NA	N
KacstDP.gene.S000143.27	KacstDP.mRNA.S000143.27	NA	N
KacstDP.gene.S000143.28	KacstDP.mRNA.S000143.28	NA	N
KacstDP.gene.S000241.20	KacstDP.mRNA.S000241.20	NA	CN
KacstDP.gene.S008522.1	KacstDP.mRNA.S008522.1	NA	N
KacstDP.gene.S000106.46	KacstDP.mRNA.S000106.46	NA	CN
KacstDP.gene.S000268.28	KacstDP.mRNA.S000268.28	NA	N
KacstDP.gene.S000054.9	KacstDP.mRNA.S000054.9	NA	CN
KacstDP.gene.S000026.10	KacstDP.mRNA.S000026.10	NA	NL
KacstDP.gene.S018023.1	KacstDP.mRNA.S018023.1	NA	N
KacstDP.gene.S000054.10	KacstDP.mRNA.S000054.10	NA	CN
KacstDP.gene.S000707.7	KacstDP.mRNA.S000707.7	NA	N
KacstDP.gene.S013153.1	KacstDP.mRNA.S013153.1	NA	N
KacstDP.gene.S000242.1	KacstDP.mRNA.S000242.1	NA	CN
KacstDP.gene.S000808.2	KacstDP.mRNA.S000808.2	NA	N

KacstDP.gene.S000427.5	KacstDP.mRNA.S000427.5	NA	N
KacstDP.gene.S001462.3	KacstDP.mRNA.S001462.3	NA	N
KacstDP.gene.S000241.16	KacstDP.mRNA.S000241.16	NA	N
KacstDP.gene.S000083.10	KacstDP.mRNA.S000083.10	NA	NL
KacstDP.gene.S000411.16	KacstDP.mRNA.S000411.16	NA	N
KacstDP.gene.S000013.89	KacstDP.mRNA.S000013.89	NA	CN
KacstDP.gene.S046239.1	KacstDP.mRNA.S046239.1	NA	N
KacstDP.gene.S010506.1	KacstDP.mRNA.S010506.1	NA	N
KacstDP.gene.S000351.2	KacstDP.mRNA.S000351.2	NA	N
KacstDP.gene.S000289.10	KacstDP.mRNA.S000289.10	NA	N
KacstDP.gene.S018174.1	KacstDP.mRNA.S018174.1	NA	N
KacstDP.gene.S016784.1	KacstDP.mRNA.S016784.1	NA	N
KacstDP.gene.S009701.1	KacstDP.mRNA.S009701.1	NA	N
KacstDP.gene.S000002.69	KacstDP.mRNA.S000002.69	NA	N
KacstDP.gene.S006308.1	KacstDP.mRNA.S006308.1	NA	N
KacstDP.gene.S026120.1	KacstDP.mRNA.S026120.1	NA	N
KacstDP.gene.S028264.1	KacstDP.mRNA.S028264.1	NA	N
KacstDP.gene.S000375.6	KacstDP.mRNA.S000375.6	NA	CN
KacstDP.gene.S000003.16	KacstDP.mRNA.S000003.16	NA	N
KacstDP.gene.S000689.1	KacstDP.mRNA.S000689.1	NA	N
KacstDP.gene.S012316.1	KacstDP.mRNA.S012316.1	NA	N
KacstDP.gene.S000488.10	KacstDP.mRNA.S000488.10	NA	N
KacstDP.gene.S002110.2	KacstDP.mRNA.S002110.2	NA	N
KacstDP.gene.S000072.19	KacstDP.mRNA.S000072.19	NA	NL
KacstDP.gene.S006035.1	KacstDP.mRNA.S006035.1	NA	N
KacstDP.gene.S003309.1	KacstDP.mRNA.S003309.1	NA	CN
KacstDP.gene.S000263.26	KacstDP.mRNA.S000263.26	NA	N

KacstDP.gene.S005985.1	KacstDP.mRNA.S005985.1	NA	CN
KacstDP.gene.S021056.1	KacstDP.mRNA.S021056.1	NA	N
KacstDP.gene.S037682.1	KacstDP.mRNA.S037682.1	NA	N
KacstDP.gene.S000064.42	KacstDP.mRNA.S000064.42	NA	N
KacstDP.gene.S015629.1	KacstDP.mRNA.S015629.1	NA	CN
KacstDP.gene.S000008.135	KacstDP.mRNA.S000008.135	NA	NL
KacstDP.gene.S000003.18	KacstDP.mRNA.S000003.18	NA	N
KacstDP.gene.S008245.1	KacstDP.mRNA.S008245.1	NA	CN
KacstDP.gene.S000285.15	KacstDP.mRNA.S000285.15	NA	N
KacstDP.gene.S000433.22	KacstDP.mRNA.S000433.22	NA	CN
KacstDP.gene.S000208.1	KacstDP.mRNA.S000208.1	NA	N
KacstDP.gene.S000133.36	KacstDP.mRNA.S000133.36	NA	CN
KacstDP.gene.S006070.1	KacstDP.mRNA.S006070.1	NA	N
KacstDP.gene.S000561.3	KacstDP.mRNA.S000561.3	NA	CN
KacstDP.gene.S000208.3	KacstDP.mRNA.S000208.3	NA	CN
KacstDP.gene.S000208.2	KacstDP.mRNA.S000208.2	NA	CNL
KacstDP.gene.S059637.1	KacstDP.mRNA.S059637.1	NA	N
KacstDP.gene.S002837.2	KacstDP.mRNA.S002837.2	NA	N
KacstDP.gene.S000451.6	KacstDP.mRNA.S000451.6	NA	N
KacstDP.gene.S003701.3	KacstDP.mRNA.S003701.3	NA	N
KacstDP.gene.S001178.1	KacstDP.mRNA.S001178.1	NA	N
KacstDP.gene.S002378.2	KacstDP.mRNA.S002378.2	NA	N
KacstDP.gene.S000492.8	KacstDP.mRNA.S000492.8	NA	N
KacstDP.gene.S000474.10	KacstDP.mRNA.S000474.10	NA	N
KacstDP.gene.S000492.7	KacstDP.mRNA.S000492.7	NA	N
KacstDP.gene.S002837.3	KacstDP.mRNA.S002837.3	NA	N
KacstDP.gene.S028798.1	KacstDP.mRNA.S028798.1	NA	L

KacstDP.gene.S000452.5	KacstDP.mRNA.S000452.5	NA	NL
KacstDP.gene.S038385.1	KacstDP.mRNA.S038385.1	NA	L
KacstDP.gene.S000433.23	KacstDP.mRNA.S000433.23	NA	NL
KacstDP.gene.S000957.7	KacstDP.mRNA.S000957.7	NA	RLK
KacstDP.gene.S000004.176	KacstDP.mRNA.S000004.176	NA	RLK
KacstDP.gene.S000083.19	KacstDP.mRNA.S000083.19	NA	RLK
KacstDP.gene.S001009.1	KacstDP.mRNA.S001009.1	NA	RLP
KacstDP.gene.S000007.98	KacstDP.mRNA.S000007.98	NA	NL
KacstDP.gene.S000044.39	KacstDP.mRNA.S000044.39	NA	RLK
KacstDP.gene.S007098.1	KacstDP.mRNA.S007098.1	NA	NL
KacstDP.gene.S000036.87	KacstDP.mRNA.S000036.87	NA	RLK
KacstDP.gene.S000457.1	KacstDP.mRNA.S000457.1	NA	RLK
KacstDP.gene.S000007.103	KacstDP.mRNA.S000007.103	NA	NL
KacstDP.gene.S000363.3	KacstDP.mRNA.S000363.3	NA	L
KacstDP.gene.S000083.65	KacstDP.mRNA.S000083.65	NA	L
KacstDP.gene.S000040.46	KacstDP.mRNA.S000040.46	NA	RLP
KacstDP.gene.S000224.1	KacstDP.mRNA.S000224.1	NA	L
KacstDP.gene.S001165.6	KacstDP.mRNA.S001165.6	NA	RLK
KacstDP.gene.S000184.30	KacstDP.mRNA.S000184.30	NA	RLK
KacstDP.gene.S017896.1	KacstDP.mRNA.S017896.1	NA	L
KacstDP.gene.S000150.4	KacstDP.mRNA.S000150.4	NA	L
KacstDP.gene.S000011.177	KacstDP.mRNA.S000011.177	NA	RLK
KacstDP.gene.S000132.34	KacstDP.mRNA.S000132.34	NA	RLK
KacstDP.gene.S000133.35	KacstDP.mRNA.S000133.35	NA	NL
KacstDP.gene.S007322.1	KacstDP.mRNA.S007322.1	NA	CNL
KacstDP.gene.S000082.34	KacstDP.mRNA.S000082.34	NA	NL
KacstDP.gene.S001439.5	KacstDP.mRNA.S001439.5	NA	CNL

KacstDP.gene.S000133.37	KacstDP.mRNA.S000133.37	NA	CNL
KacstDP.gene.S030460.1	KacstDP.mRNA.S030460.1	NA	L
KacstDP.gene.S054652.1	KacstDP.mRNA.S054652.1	NA	L
KacstDP.gene.S000056.9	KacstDP.mRNA.S000056.9	NA	RLP
KacstDP.gene.S000060.11	KacstDP.mRNA.S000060.11	NA	CNL
KacstDP.gene.S000060.13	KacstDP.mRNA.S000060.13	NA	CNL
KacstDP.gene.S000486.3	KacstDP.mRNA.S000486.3	NA	CNL
KacstDP.gene.S000073.48	KacstDP.mRNA.S000073.48	NA	RLP
KacstDP.gene.S000243.5	KacstDP.mRNA.S000243.5	NA	RLP
KacstDP.gene.S023096.1	KacstDP.mRNA.S023096.1	NA	L
KacstDP.gene.S000169.5	KacstDP.mRNA.S000169.5	NA	RLK
KacstDP.gene.S002431.1	KacstDP.mRNA.S002431.1	NA	RLP
KacstDP.gene.S027736.1	KacstDP.mRNA.S027736.1	NA	L
KacstDP.gene.S000222.18	KacstDP.mRNA.S000222.18.1	NA	RLP
KacstDP.gene.S000821.3	KacstDP.mRNA.S000821.3	NA	RLP
KacstDP.gene.S021363.1	KacstDP.mRNA.S021363.1	NA	RLP
KacstDP.gene.S000285.18	KacstDP.mRNA.S000285.18	NA	RLP
KacstDP.gene.S000075.11	KacstDP.mRNA.S000075.11	NA	RLP
KacstDP.gene.S000024.12	KacstDP.mRNA.S000024.12	NA	RLK
KacstDP.gene.S002141.1	KacstDP.mRNA.S002141.1	NA	RLK
KacstDP.gene.S011365.1	KacstDP.mRNA.S011365.1	NA	NL
KacstDP.gene.S000524.10	KacstDP.mRNA.S000524.10	NA	RLK
KacstDP.gene.S000115.62	KacstDP.mRNA.S000115.62	NA	RLP
KacstDP.gene.S000262.7	KacstDP.mRNA.S000262.7	NA	RLP
KacstDP.gene.S000061.65	KacstDP.mRNA.S000061.65	NA	RLK
KacstDP.gene.S001117.1	KacstDP.mRNA.S001117.1	NA	NL
KacstDP.gene.S001117.5	KacstDP.mRNA.S001117.5	NA	NL

KacstDP.gene.S002006.3	KacstDP.mRNA.S002006.3	NA	NL
KacstDP.gene.S000112.79	KacstDP.mRNA.S000112.79	NA	L
KacstDP.gene.S000130.52	KacstDP.mRNA.S000130.52	NA	NL
KacstDP.gene.S000445.7	KacstDP.mRNA.S000445.7	NA	RLK
KacstDP.gene.S000071.50	KacstDP.mRNA.S000071.50	NA	RLK
KacstDP.gene.S000291.8	KacstDP.mRNA.S000291.8	NA	RLK
KacstDP.gene.S000138.9	KacstDP.mRNA.S000138.9	NA	RLK
KacstDP.gene.S000005.15	KacstDP.mRNA.S000005.15	NA	RLK
KacstDP.gene.S000063.75	KacstDP.mRNA.S000063.75	NA	RLK
KacstDP.gene.S002085.2	KacstDP.mRNA.S002085.2	NA	CNL
KacstDP.gene.S016199.1	KacstDP.mRNA.S016199.1	NA	L
KacstDP.gene.S004849.1	KacstDP.mRNA.S004849.1	NA	RLP
KacstDP.gene.S000472.10	KacstDP.mRNA.S000472.10	NA	RLK
KacstDP.gene.S000002.294	KacstDP.mRNA.S000002.294	NA	RLK
KacstDP.gene.S007338.1	KacstDP.mRNA.S007338.1	NA	RLK
KacstDP.gene.S034319.1	KacstDP.mRNA.S034319.1	NA	L
KacstDP.gene.S000003.137	KacstDP.mRNA.S000003.137	NA	RLK
KacstDP.gene.S000399.4	KacstDP.mRNA.S000399.4	NA	RLK
KacstDP.gene.S000473.1	KacstDP.mRNA.S000473.1	NA	RLK
KacstDP.gene.S000473.4	KacstDP.mRNA.S000473.4	NA	RLK
KacstDP.gene.S000826.5	KacstDP.mRNA.S000826.5	NA	NL
KacstDP.gene.S000122.52	KacstDP.mRNA.S000122.52	NA	L
KacstDP.gene.S001339.3	KacstDP.mRNA.S001339.3	NA	RLP
KacstDP.gene.S000279.16	KacstDP.mRNA.S000279.16	NA	RLK
KacstDP.gene.S000099.46	KacstDP.mRNA.S000099.46	NA	RLK
KacstDP.gene.S000464.11	KacstDP.mRNA.S000464.11	NA	NL
KacstDP.gene.S000097.20	KacstDP.mRNA.S000097.20	NA	RLP

KacstDP.gene.S000445.10	KacstDP.mRNA.S000445.10	NA	RLK
KacstDP.gene.S000044.43	KacstDP.mRNA.S000044.43	NA	RLK
KacstDP.gene.S008848.1	KacstDP.mRNA.S008848.1	NA	RLP
KacstDP.gene.S010147.1	KacstDP.mRNA.S010147.1	NA	RLP
KacstDP.gene.S000293.23	KacstDP.mRNA.S000293.23	NA	RLK
KacstDP.gene.S000456.3	KacstDP.mRNA.S000456.3	NA	RLP
KacstDP.gene.S000823.5	KacstDP.mRNA.S000823.5	NA	RLP
KacstDP.gene.S000018.37	KacstDP.mRNA.S000018.37	NA	RLP
KacstDP.gene.S000371.7	KacstDP.mRNA.S000371.7	NA	RLP
KacstDP.gene.S000058.18	KacstDP.mRNA.S000058.18	NA	RLP
KacstDP.gene.S000539.1	KacstDP.mRNA.S000539.1	NA	RLK
KacstDP.gene.S000224.5	KacstDP.mRNA.S000224.5	NA	RLK
KacstDP.gene.S006332.1	KacstDP.mRNA.S006332.1	NA	RLP
KacstDP.gene.S000002.n50	KacstDP.mRNA.S000002.n56	NA	L
KacstDP.gene.S020864.1	KacstDP.mRNA.S020864.1	NA	NL
KacstDP.gene.S000033.11	KacstDP.mRNA.S000033.11	NA	NL
KacstDP.gene.S000208.32	KacstDP.mRNA.S000208.32	NA	CNL
KacstDP.gene.S001703.1	KacstDP.mRNA.S001703.1	NA	RLK
KacstDP.gene.S000007.100	KacstDP.mRNA.S000007.100.1	NA	NL
KacstDP.gene.S000007.100	KacstDP.mRNA.S000007.100	NA	NL
KacstDP.gene.S000204.6	KacstDP.mRNA.S000204.6	NA	RLP
KacstDP.gene.S000806.2	KacstDP.mRNA.S000806.2	NA	RLP
KacstDP.gene.S000664.2	KacstDP.mRNA.S000664.2	NA	RLK
KacstDP.gene.S000486.10	KacstDP.mRNA.S000486.10	NA	NL
KacstDP.gene.S000359.2	KacstDP.mRNA.S000359.2	NA	RLP
KacstDP.gene.S001169.3	KacstDP.mRNA.S001169.3	NA	RLK
KacstDP.gene.S000199.5	KacstDP.mRNA.S000199.5	NA	RLK

KacstDP.gene.S000014.12	KacstDP.mRNA.S000014.12	NA	RLK
KacstDP.gene.S015947.1	KacstDP.mRNA.S015947.1	NA	NL
KacstDP.gene.S040735.1	KacstDP.mRNA.S040735.1	NA	NL
KacstDP.gene.S000208.28	KacstDP.mRNA.S000208.28	NA	CNL
KacstDP.gene.S000012.46	KacstDP.mRNA.S000012.46	NA	RLK
KacstDP.gene.S000541.7	KacstDP.mRNA.S000541.7	NA	RLK
KacstDP.gene.S000199.3	KacstDP.mRNA.S000199.3	NA	L
KacstDP.gene.S000110.29	KacstDP.mRNA.S000110.29	NA	RLK
KacstDP.gene.S000096.22	KacstDP.mRNA.S000096.22	NA	RLK
KacstDP.gene.S000407.6	KacstDP.mRNA.S000407.6	NA	RLK
KacstDP.gene.S025145.1	KacstDP.mRNA.S025145.1	NA	L
KacstDP.gene.S006455.1	KacstDP.mRNA.S006455.1	NA	L
KacstDP.gene.S000096.38	KacstDP.mRNA.S000096.38	NA	RLK
KacstDP.gene.S000160.20	KacstDP.mRNA.S000160.20	NA	RLK
KacstDP.gene.S000073.49	KacstDP.mRNA.S000073.49	NA	L
KacstDP.gene.S000566.4	KacstDP.mRNA.S000566.4	NA	RLP
KacstDP.gene.S000806.1	KacstDP.mRNA.S000806.1	NA	RLP
KacstDP.gene.S000080.50	KacstDP.mRNA.S000080.50	NA	RLP
KacstDP.gene.S000273.11	KacstDP.mRNA.S000273.11	NA	RLK
KacstDP.gene.S000286.11	KacstDP.mRNA.S000286.11	NA	RLK
KacstDP.gene.S002782.1	KacstDP.mRNA.S002782.1	NA	NL
KacstDP.gene.S000032.83	KacstDP.mRNA.S000032.83	NA	RLK
KacstDP.gene.S000923.3	KacstDP.mRNA.S000923.3	NA	CNL
KacstDP.gene.S000074.54	KacstDP.mRNA.S000074.54	NA	RLK
KacstDP.gene.S000007.137	KacstDP.mRNA.S000007.137	NA	CNL
KacstDP.gene.S017381.1	KacstDP.mRNA.S017381.1	NA	RLK
KacstDP.gene.S000457.19	KacstDP.mRNA.S000457.19	NA	RLK

KacstDP.gene.S009861.1	KacstDP.mRNA.S009861.1	NA	RLP
KacstDP.gene.S000280.23	KacstDP.mRNA.S000280.23	NA	RLK
KacstDP.gene.S000280.24	KacstDP.mRNA.S000280.24	NA	RLK
KacstDP.gene.S000235.13	KacstDP.mRNA.S000235.13	NA	RLK
KacstDP.gene.S000006.16	KacstDP.mRNA.S000006.16	NA	L
KacstDP.gene.S001182.7	KacstDP.mRNA.S001182.7	NA	L
KacstDP.gene.S000199.10	KacstDP.mRNA.S000199.10	NA	RLP
KacstDP.gene.S000199.6	KacstDP.mRNA.S000199.6	NA	RLK
KacstDP.gene.S000350.20	KacstDP.mRNA.S000350.20	NA	RLK
KacstDP.gene.S000464.21	KacstDP.mRNA.S000464.21	NA	CNL
KacstDP.gene.S007380.1	KacstDP.mRNA.S007380.1	NA	CNL
KacstDP.gene.S000268.10	KacstDP.mRNA.S000268.10	NA	L
KacstDP.gene.S000735.6	KacstDP.mRNA.S000735.6	NA	NL
KacstDP.gene.S000397.11	KacstDP.mRNA.S000397.11	NA	RLK
KacstDP.gene.S020504.1	KacstDP.mRNA.S020504.1	NA	L
KacstDP.gene.S015641.1	KacstDP.mRNA.S015641.1	NA	RLK
KacstDP.gene.S000432.12	KacstDP.mRNA.S000432.12	NA	RLP
KacstDP.gene.S000252.30	KacstDP.mRNA.S000252.30	NA	L
KacstDP.gene.S000004.220	KacstDP.mRNA.S000004.220	NA	RLK
KacstDP.gene.S000733.6	KacstDP.mRNA.S000733.6	NA	RLK
KacstDP.gene.S000733.6	KacstDP.mRNA.S000733.6.1	NA	RLK
KacstDP.gene.S000274.17	KacstDP.mRNA.S000274.17	NA	RLK
KacstDP.gene.S011673.1	KacstDP.mRNA.S011673.1	NA	NL
KacstDP.gene.S001716.1	KacstDP.mRNA.S001716.1	NA	NL
KacstDP.gene.S001974.1	KacstDP.mRNA.S001974.1	NA	NL
KacstDP.gene.S001293.1	KacstDP.mRNA.S001293.1	NA	NL
KacstDP.gene.S013440.1	KacstDP.mRNA.S013440.1	NA	L

KacstDP.gene.S000733.6	KacstDP.mRNA.S000733.6.2	NA	RLK
KacstDP.gene.S000733.6	KacstDP.mRNA.S000733.6.2.1	NA	RLK
KacstDP.gene.S003877.1	KacstDP.mRNA.S003877.1	NA	L
KacstDP.gene.S000808.3	KacstDP.mRNA.S000808.3	NA	L
KacstDP.gene.S001620.2	KacstDP.mRNA.S001620.2	NA	NL
KacstDP.gene.S017604.1	KacstDP.mRNA.S017604.1	NA	L
KacstDP.gene.S000065.75	KacstDP.mRNA.S000065.75	NA	RLK
KacstDP.gene.S000073.69	KacstDP.mRNA.S000073.69	NA	L
KacstDP.gene.S000602.9	KacstDP.mRNA.S000602.9	NA	RLK
KacstDP.gene.S000486.4	KacstDP.mRNA.S000486.4	NA	RLK
KacstDP.gene.S000291.2	KacstDP.mRNA.S000291.2	NA	NL
KacstDP.gene.S000138.31	KacstDP.mRNA.S000138.31	NA	NL
KacstDP.gene.S002174.1	KacstDP.mRNA.S002174.1	NA	NL
KacstDP.gene.S001902.1	KacstDP.mRNA.S001902.1	NA	CNL
KacstDP.gene.S001902.3	KacstDP.mRNA.S001902.3	NA	NL
KacstDP.gene.S000359.1	KacstDP.mRNA.S000359.1	NA	RLP
KacstDP.gene.S007981.1	KacstDP.mRNA.S007981.1	NA	NL
KacstDP.gene.S000049.40	KacstDP.mRNA.S000049.40	NA	RLK
KacstDP.gene.S000619.5	KacstDP.mRNA.S000619.5	NA	RLK
KacstDP.gene.S000116.29	KacstDP.mRNA.S000116.29	NA	RLK
KacstDP.gene.S004663.1	KacstDP.mRNA.S004663.1	NA	RLK
KacstDP.gene.S000391.13	KacstDP.mRNA.S000391.13	NA	CNL
KacstDP.gene.S000395.24	KacstDP.mRNA.S000395.24	NA	RLK
KacstDP.gene.S036948.1	KacstDP.mRNA.S036948.1	NA	L
KacstDP.gene.S029340.1	KacstDP.mRNA.S029340.1	NA	NL
KacstDP.gene.S000019.62	KacstDP.mRNA.S000019.62	NA	RLK
KacstDP.gene.S000006.4	KacstDP.mRNA.S000006.4	NA	NL

KacstDP.gene.S000682.4	KacstDP.mRNA.S000682.4	NA	CNL
KacstDP.gene.S008858.1	KacstDP.mRNA.S008858.1	NA	RLP
KacstDP.gene.S000492.9	KacstDP.mRNA.S000492.9	NA	RLP
KacstDP.gene.S000005.93	KacstDP.mRNA.S000005.93	NA	RLP
KacstDP.gene.S000452.3	KacstDP.mRNA.S000452.3	NA	CNL
KacstDP.gene.S000391.19	KacstDP.mRNA.S000391.19	NA	NL
KacstDP.gene.S001601.3	KacstDP.mRNA.S001601.3	NA	NL
KacstDP.gene.S004708.1	KacstDP.mRNA.S004708.1	NA	NL
KacstDP.gene.S001015.6	KacstDP.mRNA.S001015.6	NA	L
KacstDP.gene.S000240.4	KacstDP.mRNA.S000240.4	NA	L
KacstDP.gene.S000085.15	KacstDP.mRNA.S000085.15	NA	RLK
KacstDP.gene.S017428.1	KacstDP.mRNA.S017428.1	NA	L
KacstDP.gene.S000488.9	KacstDP.mRNA.S000488.9	NA	NL
KacstDP.gene.S000669.1	KacstDP.mRNA.S000669.1	NA	NL
KacstDP.gene.S000003.153	KacstDP.mRNA.S000003.153	NA	NL
KacstDP.gene.S000561.15	KacstDP.mRNA.S000561.15	NA	NL
KacstDP.gene.S001304.3	KacstDP.mRNA.S001304.3	NA	NL
KacstDP.gene.S000216.8	KacstDP.mRNA.S000216.8	NA	RLP
KacstDP.gene.S001304.5	KacstDP.mRNA.S001304.5	NA	NL
KacstDP.gene.S000064.13	KacstDP.mRNA.S000064.13	NA	NL
KacstDP.gene.S000036.89	KacstDP.mRNA.S000036.89	NA	RLK
KacstDP.gene.S000079.75	KacstDP.mRNA.S000079.75	NA	RLK
KacstDP.gene.S000474.2	KacstDP.mRNA.S000474.2	NA	NL
KacstDP.gene.S000049.12	KacstDP.mRNA.S000049.12	NA	NL
KacstDP.gene.S006592.1	KacstDP.mRNA.S006592.1	NA	L
KacstDP.gene.S000730.5	KacstDP.mRNA.S000730.5	NA	RLK
KacstDP.gene.S000012.124	KacstDP.mRNA.S000012.124	NA	CNL

KacstDP.gene.S001114.7	KacstDP.mRNA.S001114.7	NA	NL
KacstDP.gene.S000254.19	KacstDP.mRNA.S000254.19	NA	NL
KacstDP.gene.S000095.7	KacstDP.mRNA.S000095.7	NA	NL
KacstDP.gene.S000049.10	KacstDP.mRNA.S000049.10	NA	NL
KacstDP.gene.S034307.1	KacstDP.mRNA.S034307.1	NA	NL
KacstDP.gene.S000826.2	KacstDP.mRNA.S000826.2	NA	NL
KacstDP.gene.S000004.175	KacstDP.mRNA.S000004.175	NA	NL
KacstDP.gene.S000026.12	KacstDP.mRNA.S000026.12	NA	NL
KacstDP.gene.S026504.1	KacstDP.mRNA.S026504.1	NA	NL
KacstDP.gene.S002082.1	KacstDP.mRNA.S002082.1	NA	NL
KacstDP.gene.S001420.1	KacstDP.mRNA.S001420.1	NA	NL
KacstDP.gene.S010979.1	KacstDP.mRNA.S010979.1	NA	NL
KacstDP.gene.S000094.40	KacstDP.mRNA.S000094.40	NA	NL
KacstDP.gene.S051619.1	KacstDP.mRNA.S051619.1	NA	NL
KacstDP.gene.S001303.3	KacstDP.mRNA.S001303.3	NA	NL
KacstDP.gene.S001875.2	KacstDP.mRNA.S001875.2	NA	NL
KacstDP.gene.S001303.4	KacstDP.mRNA.S001303.4	NA	NL
KacstDP.gene.S001875.3	KacstDP.mRNA.S001875.3	NA	NL
KacstDP.gene.S000488.8	KacstDP.mRNA.S000488.8	NA	NL
KacstDP.gene.S000033.12	KacstDP.mRNA.S000033.12	NA	NL
KacstDP.gene.S000007.99	KacstDP.mRNA.S000007.99	NA	NL
KacstDP.gene.S000255.31	KacstDP.mRNA.S000255.31	NA	NL
KacstDP.gene.S000003.193	KacstDP.mRNA.S000003.193	NA	NL
KacstDP.gene.S001303.5	KacstDP.mRNA.S001303.5	NA	NL
KacstDP.gene.S012723.1	KacstDP.mRNA.S012723.1	NA	NL
KacstDP.gene.S000513.3	KacstDP.mRNA.S000513.3	NA	CNL
KacstDP.gene.S001168.3	KacstDP.mRNA.S001168.3	NA	NL

KacstDP.gene.S013011.1	KacstDP.mRNA.S013011.1	NA	NL
KacstDP.gene.S000513.4	KacstDP.mRNA.S000513.4	NA	NL
KacstDP.gene.S001114.5	KacstDP.mRNA.S001114.5	NA	NL
KacstDP.gene.S000140.28	KacstDP.mRNA.S000140.28	NA	NL
KacstDP.gene.S000162.10	KacstDP.mRNA.S000162.10	NA	NL
KacstDP.gene.S000289.8	KacstDP.mRNA.S000289.8	NA	CNL
KacstDP.gene.S002110.3	KacstDP.mRNA.S002110.3	NA	NL
KacstDP.gene.S000521.14	KacstDP.mRNA.S000521.14	NA	NL
KacstDP.gene.S001164.1	KacstDP.mRNA.S001164.1	NA	NL
KacstDP.gene.S009287.1	KacstDP.mRNA.S009287.1	NA	NL
KacstDP.gene.S000013.192	KacstDP.mRNA.S000013.192	NA	NL
KacstDP.gene.S002428.1	KacstDP.mRNA.S002428.1	NA	NL
KacstDP.gene.S000289.24	KacstDP.mRNA.S000289.24	NA	CNL
KacstDP.gene.S008209.1	KacstDP.mRNA.S008209.1	NA	NL
KacstDP.gene.S000007.155	KacstDP.mRNA.S000007.155	NA	CNL
KacstDP.gene.S000290.22	KacstDP.mRNA.S000290.22	NA	NL
KacstDP.gene.S002178.1	KacstDP.mRNA.S002178.1	NA	NL
KacstDP.gene.S000375.7	KacstDP.mRNA.S000375.7	NA	NL
KacstDP.gene.S000826.3	KacstDP.mRNA.S000826.3	NA	NL
KacstDP.gene.S000890.2	KacstDP.mRNA.S000890.2	NA	CNL
